# Isolation, characterization and toxicological potential of *Alternaria-*mycotoxins (TeA, AOH and AME) in different *Alternaria* species from various regions of India

**DOI:** 10.1038/s41598-017-09138-9

**Published:** 2017-08-18

**Authors:** Mukesh Meena, Prashant Swapnil, R. S. Upadhyay

**Affiliations:** Department of Botany, Institute of Science, Banaras Hindu University, Varanasi, 221005 India

## Abstract

*Alternaria* species produce various sorts of toxic metabolites during their active growth and causes severe diseases in many plants by limiting their productivity. These toxic metabolites incorporate various mycotoxins comprising of dibenzo-α-pyrone and some tetramic acid derivatives. In this study, we have screened out total 48 isolates of *Alternaria* from different plants belonging to different locations in India, on the basis of their pathogenic nature. Pathogenicity testing of these 48 strains on susceptible tomato variety (CO-3) showed 27.08% of the strains were highly pathogenic, 35.41% moderately pathogenic and 37.5% were less pathogenic. Phylogenetic analysis showed the presence of at least eight evolutionary cluster of the pathogen. Toxins (TeA, AOH and AME) were isolated, purified on the basis of column chromatography and TLC, and further confirmed by the HPLC-UV chromatograms using standards. The final detection of toxins was done by the LC-MS/MS analysis by their mass/charge ratio. The present study develops an approach to classify the toxicogenic effect of each of the individual mycotoxins on tomato plant and focuses their differential susceptibility to develop disease symptoms. This study represents the report of the natural occurrence and distribution of *Alternaria* toxins in various plants from India.

## Introduction

*Alternaria* is one of the most common fungal genera found ubiquitously and comprises of species which may be saprophytic, endophytic or pathogenic in nature. The small spores of this pathogen are distributed everywhere where they can deteriorate food quality and quantity, and decrease their nutritive profile by producing some potent toxic metabolites and hence degrade the economic values of food products and other animal feedstuffs. As phytopathogens, they can cause severe problems in agriculture by reducing crop yield, thus causing considerable economic losses to farmers and food processing industries^1–3^. The phytotoxic effect first appears in leaves followed by their progressive contamination to fruits hence deteriorating the tomato fruits, affecting pulp quality and overall decreasing the fruit quantity and quality at harvesting stage hence decreasing the economic value of fruits. The diseased symptoms develops during pathogenesis is due to the phytotoxicity of fungal metabolites produced during their active growth^4^ and has been demonstrated through many preliminary studies^3, 5^.

Toxic metabolites secreted by *Alternaria* species can be categorized into three major structural categories^3, 5^ (i) Dibenzo-α-pyrone derivatives which are exemplified by alternariol (AOH), alternariol monomethyl ether (AME) and altenuene (ALT); (ii) Perylene derivatives which includes altertoxins (ATX-I, -II and -III) and (iii) Tetramic acid derivative which contain tenuazonic acid (TeA). The chemical composition of three mycotoxins commonly produced by *Alternaria* species are; (i) AOH (C_14_H_10_O_5_): 3,7,9-trihydroxy-1-methyl-6*H*-dibenzo(*b,d*)pyran-6-one; M.W. 258; (ii) AME (C_15_H_12_O_5_): 3,7-dihydroxy-9-methoxy-1-methyl-6*H*-dibenzo(*b,d*)pyran-6-one; M.W. 272; (iii) TeA (C_10_H_15_O_3_N): 3-acetyl-5-*sec*-butyl-4-hydroxy-3-pyrrolin-2-one; M.W. 197.

TeA, iso-tenuazonic acid, and their salts exhibit herbicidal activity with broad spectrum properties, quick killing, and high efficiency for plants^6^. TeA was also found in Canadian lentils, and in recently found in beer and cereal foods^7–10^. The addition of adjuvants improves the herbicidal activity of these compounds. Recently, many *in vitro* studies have reported that AOH causes DNA damage by inducing cell cycle arrest^11, 12^ which leads to mutations in living beings^13–15^. Furthermore, AOH also exhibits cytotoxic, foetotoxic, mutagenic and teratogenic effects that is responsible for the etiology of oesophageal cancer^16^. It has been showed that both AME and AOH have potential carcinogenic, genotoxic and cytotoxic activity in both microbial and mammalian cell system^16^. According to Graf *et al*.^17^ in case of *Alternaria alternata*, an external addition of alternariol restored the pathogenicity. Many other fungal genera such as *Stagonospora nodorum* ^18^ and *Phomopsis* isolates^19^ have also been found to produce AOH and AME. TeA is also produced by other species of fungi including, *Pyricularia oryzae* and *Phoma sorghina* ^3, 20–22^.

During the last decade, there is growing interest in isolation, purification and characterization of *Alternaria* toxins. *Alternaria* mycotoxins have been frequently isolated and reported in fruits and vegetables, such as tomatoes, citrus fruits, Japanese pears, prune nectar, red currant, carrots, barley, oats, olives, mandarins, melons, peppers, apples, raspberries, cranberries, grapes, sunflower seeds, oilseed rape meals, flax seeds, linseeds, pecans, melons, lentils, wheat and other grains^1–3, 5, 23–29^. Recently *Alternaria* mycotoxins have been analysed and determined using some of the advanced, highly developed and separation techniques such as thin-layer chromatography (TLC), high performance liquid chromatography (HPLC), high performance thin-layer chromatography (HPTLC), and gas chromatography (GC) techniques^30–33^. However, in all the above techniques HPLC is the most extensively used technique for the detection of *Alternaria* toxins^3, 25, 27, 34^. In the present time, LC-MS/MS is highly selective, sensitive, and accurate technique for mycotoxin determination in both biological^35, 36^ and food samples^37^.

Recent investigations have explored the availability of different mycotoxins isolated from *Alternaria* confined to different geographical regions of the world. However, very inadequate information is available for *Alternaria* pathogen recovered from Indian subcontinent. The broad spectrum pathogenicity of the isolates recovered from different locations is determined by various environmental parameters. Overall the differential host response in the presence of host defense mechanisms against pathogens determines the degree of susceptibility or resistance of host plants. The isolation, purification and characterization of different mycotoxins provide information regarding the severity of the pathogen and its toxic effects caused by the cumulative action of all these toxins. The individual action of single mycotoxin for disease development is incurred by its efficiency and the degree of damages. In this regard, the effect of each toxic component varies among different isolates. The elucidation of the functional pathway lies behind the biogenesis, action mechanism, signalling cascades involved and the relevant host mediated defense response in presence of these mycotoxins will assist pathogen controlling and disease development.

The present research work focuses on the isolation, identification and characterisation of different mycotoxins by various liquid chromatography (LC) techniques from different isolates of *Alternaria* species. The study also investigates the differential toxic effect of these isolates against tomato plant, and the efficiency and potential of each of the three mycotoxins TeA, AOH and AME in disease development.

## Results

### Morphological identification

**Figure 1:**
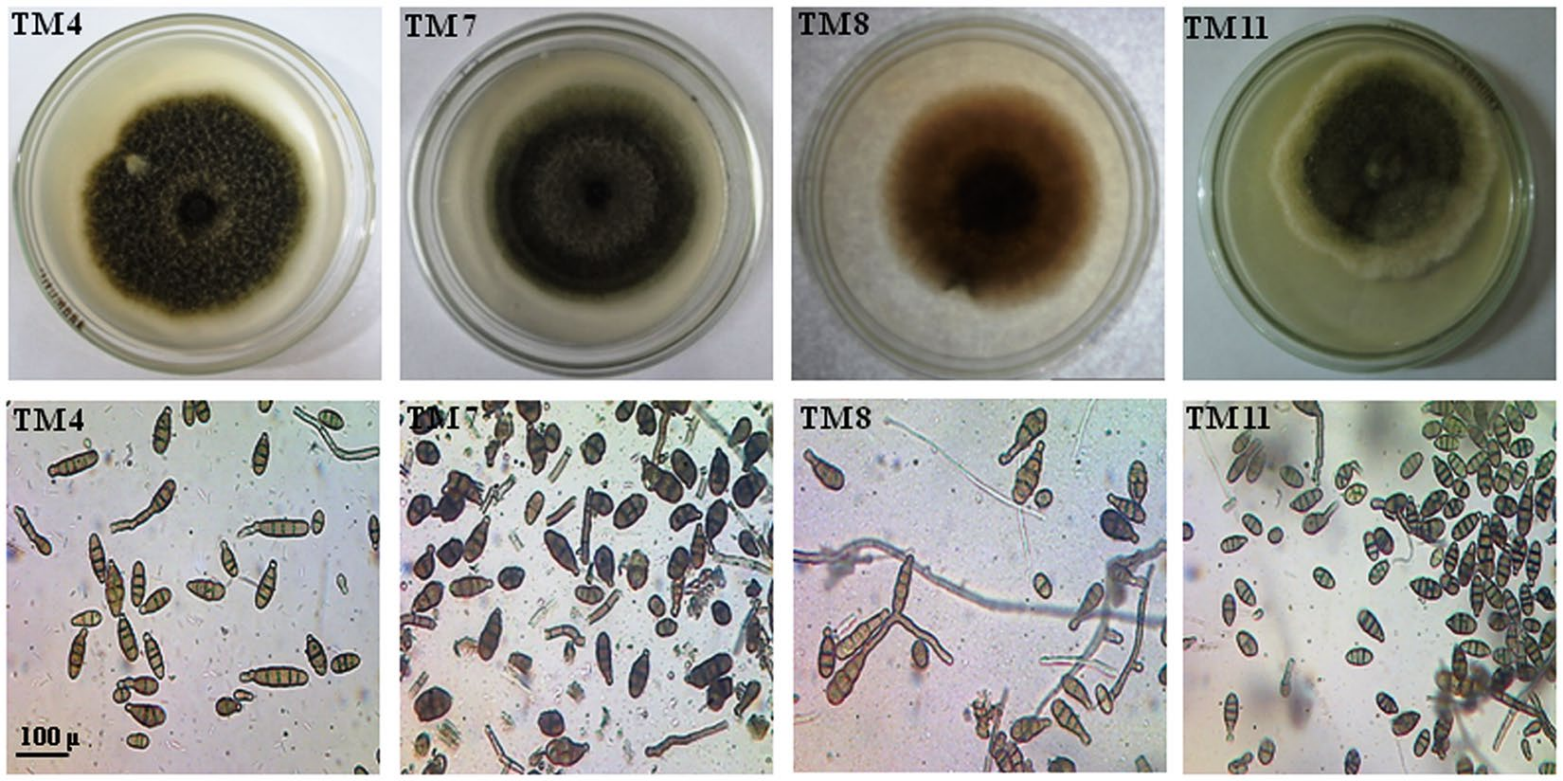
Morphological characteristics, growth pattern, colony morphology and microscopic examination of three potent toxic isolates of *Alternaria* species collected from different regions in India. All the *Alternaria* isolated were grown on PDA culture media and incubated at 28 °C for 12 h light/dark photoperiod. The pictures of the colonies were taken at 6^th^ day after incubation of pathogen. Note: The names of the *Alternaria* isolates were given as abbreviation of the different plants from which they were isolated (details described in Table 1).

A total of 60 isolates of *Alternaria* have been isolated from various regions in India. Out of them 48 isolates of *Alternaria* were selected in the present study on the basis of their high pathogenic nature. Microscopic examination was made to identify these 48 isolates on the basis of sporulation pattern on culture plates (Supplementary Fig. S1). Further, these isolates were confirmed on the basis of their morphological characteristics including length of primary conidiophores, branching patterns, origin of branching, conidial shapes, size and colour with ornamentation pattern. *Alternaria* species were identified by employing compound microscope at 40X magnification with following standard manuals^38, 39^. The isolates were identified as belonging to *Alternaria* genera on the basis of some morphological characteristics like conidial structure, presence or absence of septa, septation pattern as mentioned in relevant scientific literatures describing key morphological characteristics available for *Alternaria* (Fig. 1 and Table 1). The *Alternaria* isolates exhibited a high level of diversity in terms of culture and morphology. The colour of the colonies and the conidia grown on potato dextrose agar (PDA)  media showed some variations. The colour of isolated *Alternaria* species varies such as light green, black green, light grey, olive brown-green, brown, white cottony and some other, which was given in Table 1. The conidia were different shapes like as long obpyriform in shape with long beak, obclavate with rounded at the apex, long and small conidia with germinating tubes, some were brown with rounded apex, some were moderate in size etc. The isolates of *Alternaria* species showed significant morphological variability in respect of conidia length, conidia width and number of septa (transverse/longitudinal septa) (Table 2). The average conidial length, which varied from 18.76 to 54.77 μm, was highest in Rohtak, Haryana mustard leaf isolated strain (MT 3; KX139154) that is, 54.77 μm and lowest in Jaunpur, Uttar Pradesh tomato leaf isolated strain (TM 11) that is, 18.76 μm. Average conidial width, which varied from 4.10 to 7.90 μm, was highest in TM 11 isolates that is 7.90 μm and lowest in Jaunpur, Uttar Pradesh isolate parthenium leaf isolated strain (PR 2; KX139166) that is, 4.10 μm. The average number of transverse septa, which varied from 1.33 to 8.67, was highest in MT 3 isolates that is 8.67 and lowest in Varanasi, Uttar Pradesh tomato leaf isolated strain (TM 1; KX179477) that is, 1.33. While, the average number of longitudinal septa, which varied from 1.0 to 4.67, was highest in Satna, Madhya Pradesh mustard leaf isolated strain (MT 5; KX139155) that is 4.67 and lowest in TM 1 isolates that is, 1.0.

**Table 1:** Plant origin, pathogenicity, colony morphology and hyphal/conidial structures of the *Alternaria* species used in study.

S. No.	Origin (Cultivar)	Plant tissue	Collection site	Geographical data	Isolates* and Accession Numbers	Pathogenicity	Colony morphology on PDA plate	Hyphae and conidial structure
**1**.	Tomato (TM) (*Lycopesicon esculentum*)	Fruit	Varanasi, Uttar Pradesh	(25°28ʹN, 82°96ʹE)	TM 1 **(KX179477)**	+	White olive brown-green cottony	Septate hyphae with ovoid to obclavate conidia, transverse and longitudinal septations,
		Stem	Varanasi, Uttar Pradesh	(25°28ʹ N, 82°96ʹ E)	TM 2 **(KX118412)**	++	White light-green	Brown and ovoid conidia with elongated apical cell
			Sawai Madhopur, Rajasthan	(25°98ʹ N, 76°36ʹ E)	TM 3 **(KX179478)**	+	White dark-green	Brown conidia with chain formation, longitudinal septations
		Leaf	Varanasi, Uttar Pradesh	(25° 28ʹ N, 82° 96ʹ E)	TM 4 **(KX118413)**	+++	Dark green	Conidia brown, transverse septation and septate hyphae
			Mirzapur, Uttar Pradesh	(25°15ʹ N, 82°60ʹ E)	TM 5 **(KX118414)**	++	Light grey	Dark brown, small and ovoid to obclavate conidia, transverse and longitudinal septations, septate hyphae
			Varanasi, Uttar Pradesh	(25°28ʹ N, 82°96ʹ E)	TM 6 **(KX118415)**	+++	Light-green with whitish	Septate hyphae, conidia light brown, short conical beak at the tip, surface smooth and septate
			Chandauli, Uttar Pradesh	(25°27ʹ N, 83°27ʹ E)	TM 7 **(KX118416)**	+++	Dark brown	Septate hyphae, conidia ovoid, several vertical and transverse septa
			Varanasi, Uttar Pradesh	(25° 28ʹ N, 82° 96ʹ E)	TM 8 **(KX118417)**	+++	Green with white margin	Ovoid to obclavate conidia, some are small and smooth, transverse septation
			Bharatpur, Rajasthan	(27°22ʹ N, 77°48ʹ E)	TM 9 **(KX118418)**	++	Green and woolly	Septate hyphae, brown, club shaped conidia, chain transverse and longitudinal septate, smooth surface
			Satna, Madhya Pradesh	(24°16ʹ N, 80°83ʹ E)	TM 10 **(KX118419)**	+++	Dark green	Long conidia, club shaped with septation, hyphae septate
			Jaunpur, Uttar Pradesh	(25°73ʹ N, 82°68ʹ E)	TM 11 **(KX118420)**	+++	Dark green with compact colony	Conidia are small, ovoid, and septate, hyphae also septate
**2**.	Brinjal (BJ) (*Solanum melongena*)	Leaf	Agra, Uttar Pradesh	(27°18ʹ N, 78°02ʹ E)	BJ 1 **(KX179479)**	+	Dark green compact	Light grey, ovoid to obclavate conidia, transverse and longitudinal septations
			Varanasi, Uttar Pradesh	(25°28ʹ N, 82°96ʹ E)	BJ 2 **(KX179480)**	+	White green cottony	Light grey, ovoid conidia, transverse and longitudinal septations
			Jaunpur Uttar Pradesh	(25°73ʹ N, 82°68ʹ E)	BJ 3 **(KX179481)**	+	Dark green colony with white margins	Light grey, obclavate conidia, some are very small, transverse and longitudinal septations
			Kota, Rajasthan	(25°18ʹ N, 75°83ʹ E)	BJ 4 **(KX179482)**	+	Green compact with cottony growth	Light gray, ovoid conidia, both (transverse and longitudinal) septation are present
			Satna, Madhya Pradesh	(24°16ʹ N, 80°83ʹ E)	BJ 5 **(KX179483)**	+	Green colony	Septate hyphae, beakless conidia, transverse and longitudinal septations
			Rohtak, Haryana	(28°89ʹ N, 76°57ʹ E)	BJ 6 **(KX179484)**	+	Green with white cottony	Brown conidia, beak at the tip of conidia, formation of chain of conidia, longitudinal and transverse septations
**3**.	Mustard (MT) (*Brassica oleracea*)	Leaf	Mirzapur, Uttar Pradesh	(25°15ʹ N, 82°60ʹ E)	MT 1 **(KX139152)**	++	Brown-white colony	Long conidia, many transverse septa, sharp beak at the tip of conidia
			Bharatpur, Rajasthan	(27°22ʹ N, 77°48ʹ E)	MT 2 **(KX139153)**	++	Grey brown woolly colony	Dark brown conidia with chain formation, transverse and longitudinal septations, beak at the tip
			Rohtak, Haryana	(28°89ʹ N, 76°57ʹ E)	MT 3 **(KX139154)**	++	Gray whitish compact colony	Brown, Long conidia, transverse septations and sharp beak at the tip of conidia
			Varanasi, Uttar Pradesh	(25°28ʹ N, 82°96ʹ E)	MT 4 **(KX118421)**	+++	Light green and whitish growth	Hyphae septate, obclavate conidia, and transverse septation
			Satna, Madhya Pradesh	(24°16ʹ N, 80°83ʹ E)	MT 5 **(KX139155)**	++	White cottony	Brown, Long and small conidia, transverse septations and sharp beak at the tip of conidia
			Sawai Madhopur, Rajasthan	(25°98ʹ N, 76°36ʹ E)	MT 6 **(KX139156)**	++	Green in the center and white at the margins	Hyphae branched and septate, gray conidia, ovoid to obclavate with sepations
			Varanasi, Uttar Pradesh	(25°28ʹ N, 82°96ʹ E)	MT 7 **(KX118425)**	+++	Green and white at the margin	Hyphae septate, conidia moderate long, brown, septate and chain formation
			Jaunpur, Uttar Pradesh	(25°73ʹ N, 82°68ʹ E)	MT 8 **(KX118426)**	+++	Light green, white at the margin	Hyphae branched and septate, conidia septate, rounded at the apex
**4**.	Potato (PT) (*Solenum tuberosum*)	Leaf	Hyderabad, Andhra Pradesh	(17°37ʹ N, 78°48ʹ E)	PT 1 **(KX139150)**	++	Dark black green	Branched and septate hyphae, conidia small oval shaped and septate
			Varanasi, Uttar Pradesh	(25°28ʹ N, 82°96ʹ E)	PT 2 **(KX139151)**	+++	Light green with concentric ring	Septate hyphae, gray conidia and septation, rounded at the apex
			Varanasi, Uttar Pradesh	(25°28ʹ N, 82°96ʹ E)	PT 3 **(KX118427)**	+++	Dark green	Septate hyphae, gray, small, long conidia with septation
**5**.	Cauliflower (CF) (*Brassica oleracea* var. *botrytis*)	Leaf	Varanasi, Uttar Pradesh	(25°28ʹ N, 82°96ʹ E)	CF 1 **(KX118422)**	+++	Olive brown light-green	Brown, taranverse and longitudinal septations
			Jaunpur, Uttar Pradesh	(25°73ʹ N, 82°68ʹ E)	CF 2 **(KX118423)**	++	Cottony compact	Dark gray conidia, small in size, transverse and longitudinal septa
			Hyderabad, Andhra Pradesh	(17°37ʹ N, 78°48ʹ E)	CF 3 **(KX118424)**	+++	Gray white	Dark brown conidia, small, long and septate, small conidia are beakless
**6**.	Pea (PE) (*Pisum sativm*)	Leaf	Varanasi, Uttar Pradesh	(25°28ʹ N, 82°96ʹ E)	PE 1 **(KX139157)**	++	Green and white at the margin	Septate hyphae, brown and septate, germinating tube also present
			Bharatpur, Rajasthan	(27°22ʹ N, 77°48ʹ E)	PE 2 **(KX179485)**	+	Light green and cottony	Gray and septate conidia, small and long conidia
		Pod	Sawai Madhopur, Rajasthan	(25°98ʹ N, 76°36ʹ E)	PE 3 **(KX139158)**	+	White woolly colony	Conidia are obclavate, septate and rounded at the apex
**7**.	Cabbage (CA) (*Brassica oleracea* var. *capitata*)	Leaf	Allahabad, Uttar Pradesh	(25°45ʹ N, 81°85ʹ E)	CA 1 **(KX139159)**	++	Light-green compact	Light brown conidia with chain formation, septate hyphae, taranverse and longitudinal septations
			Varanasi, Uttar Pradesh	(25°28ʹ N, 82°96ʹ E)	CA 2 **(KX139160)**	++	Dark green with concentric ring	Dark gray, small, conidia, transverse and longitudinal septation are present
**8**.	Spinach (SP) (*Spinacia oleracea*)	Leaf	Varanasi, Uttar Pradesh	(25°28ʹ N, 82°96ʹ E)	SP 1 **(KX179486)**	+	Dark brown	Dark brown, small, ovoid conidia, transverse and longitudinal septation
			Sawai Madhopur Rajasthan	(25°98ʹ N, 76°36ʹ E)	SP 2 **(KX179487)**	+	Light brown	Light gray conidia, rounded at the tip and both longitudinal and transverse septa are present
**9**.	Onion (ON) (*Allium cepa*)	Leaf	Varanasi, Uttar Pradesh	(25°28ʹ N, 82°96ʹ E)	ON 1 **(KX139161)**	+	Dark green with white margins	Septate hyphae, small conidia, transverse and longitudinal septation
			Bharatpur, Rajasthan	(27°22ʹ N, 77°48ʹ E)	ON 2 **(KX139162)**	+	Green and white	Septate hyphae, gray and light brown conidia with septations
**10**.	Cicer (CR) (*Cicer arientinum*)	Leaf	Varanasi, Uttar Pradesh	(25°28ʹ N, 82°96ʹ E)	CR 1 **(KX179488)**	+	Green	Septate hyphae, germinate in the chain formation, brown with septation
			Sawai Madhopur, Rajasthan	(25°98ʹ N, 76°36ʹ E)	CR 2 **(KX179489)**	+	Green and white at margin	Dark gray, small and moderate long with septa
**11**.	Eichhornia (EC) (*Echhornia crassipes*)	Leaf	Varanasi, Uttar Pradesh	(25°28ʹ N, 82°96ʹ E)	EC 1 **(KX139163)**	++	Dark green	Septate hyphae with elongated conidia, transverse and longitudinal septations
			Satna, Madhya Pradesh	(24°16ʹ N, 80°83ʹ E)	EC 2 **(KX139164)**	++	Green compact with white at margins	Gray, long and small conidia with germinating tube, hyphae septate, transverse sepate
**12**.	Lantana (LT) (*Lentana camara*)	Leaf	Varanasi, Uttar Pradesh	(25°28ʹ N, 82°96ʹ E)	LT 1 **(KX179490)**	+	Light green	Brown, small, septate, and oval shaped conidia
			Bharatpur, Rajasthan	(27°22ʹ N, 77°48ʹ E)	LT 2 **(KX179491)**	+	White cottony	Gray, septate, rounded at the apex
**13**.	Parthenium (PR) (*Parthenium hysterophorus*)	Leaf	Varanasi, Uttar Pradesh	(25°28ʹ N, 82°96ʹ E)	PR 1 **(KX139165)**	++	White-Green cottony	Light olive brown, elongated and septations with septate hyphae
			Jaunpur, Uttar Pradesh	(25°73ʹ N, 82°68ʹ E)	PR 2 **(KX139166)**	++	Green with woolly growth	Light brown, septate hyphae, transverse and longitudinal septa, tip rounded

**Note: +++** Highly pathogenic (60–100%); ++ Moderate pathogenic (30–60%); +Less pathogenic (10–30%) (Pathogenicity tests were performed on susceptible tomato (CO-3) plants varity)

*The names of the *Alternaria* isolates were given as abbreviation of the different plants from which they were isolated

All the morphological characters such as culture appearance on PDA plate, mycelia colour, and conidia appearance were observed and data were collected.

**Table 2:** Measurement of different morphological structures of isolated *Alternaria* species.

S. No.	Isolates	Average conidial Length (μm)	Average conidial Breadth (μm)	L:W ratio	Average number of transverse septa/longitudinal septa
1.	TM 1	24.32	5.20	4.68	1.33/1.0
2.	TM 2	28.40	6.10	4.66	1.67/1.33
3.	TM 3	25.20	6.40	3.94	3.67/1.33
4.	TM 4	30.98	5.50	5.63	4.00/1.67
5.	TM 5	20.20	7.80	2.59	2.33/2.33
6.	TM 6	32.28	6.20	5.21	3.33/1.67
7.	TM 7	22.36	7.80	2.87	2.67/1.67
8.	TM 8	34.64	5.40	6.41	3.33/2.67
9.	TM 9	38.98	5.80	6.72	4.33/1.33
10.	TM 10	40.16	6.60	6.08	3.67/1.67
11.	TM 11	18.76	7.90	2.37	4.67/2.33
12.	BJ 1	20.88	6.10	3.42	3.67/2.67
13.	BJ 2	21.35	6.60	3.23	2.67/2.33
14.	BJ 3	27.65	7.70	3.59	3.33/2.67
15.	BJ 4	29.11	6.40	4.55	3.67/1.67
16.	BJ 5	28.39	7.30	3.89	3.33/2.0
17.	BJ 6	27.48	5.40	5.09	2.67/1.33
18.	MT 1	47.99	8.70	5.52	7.67/4.33
19.	MT 2	51.62	8.90	5.80	8.33/4.67
20.	MT 3	54.77	8.60	6.37	8.67/5.33
21.	MT 4	38.39	6.30	6.09	6.33/3.33
22.	MT 5	50.35	7.70	6.54	7.67/4.67
23.	MT 6	33.87	5.80	5.84	4.33/2.33
24.	MT 7	37.99	7.50	5.07	4.67/2.67
25.	MT 8	32.56	4.20	7.75	3.33/1.67
26.	PT 1	27.35	5.40	5.06	2.67/1.33
27.	PT 2	31.36	5.60	5.60	1.67/1.0
28.	PT 3	28.16	5.40	5.21	3.33/1.67
29.	CF 1	25.08	7.50	3.34	2.0/1.33
30.	CF 2	22.98	7.60	3.02	2.33/1.67
31.	CF 3	21.64	6.50	3.33	2.67/1.67
32.	PE 1	24.04	5.40	4.45	2.33/1.0
33.	PE 2	23.76	4.80	4.95	2.0/1.33
34.	PE 3	24.88	5.10	4.88	2.33/1.67
35.	CA 1	24.77	5.40	4.59	2.63/2.0
36.	CA 2	25.87	5.50	4.70	2.67/1.67
37.	SP 1	26.35	5.60	4.71	2.0/1.0
38.	SP 2	27.39	4.40	6.23	3.33/1.33
39.	ON 1	23.99	5.60	4.28	2.67/1.67
40.	ON 2	25.11	6.70	3.75	2.33/1.33
41.	CR 1	37.62	7.30	5.15	4.33/2.33
42.	CR 2	35.99	6.70	5.37	3.67/2.0
43.	EC 1	34.04	6.50	5.24	4.33/2.0
44.	EC 2	33.87	5.10	6.64	4.67/1.67
45.	LT 1	26.76	4.20	6.37	2.0/1.0
46.	LT 2	35.65	6.30	5.66	2.0/1.67
47.	PR 1	36.48	6.70	5.44	3.67/1.33
48.	PR 2	28.87	4.10	7.04	2.67/1.33

Note: The size and shape of conidia length and width (L:W) was determined using ocular and stage micrometer. Numbers of septa (transverse/longitudinal septa) were also recorded.

Finally, it was revealed that the smallest size of conidia and lowest number of septa was seen in TM 11, PR 2 and TM 1 isolates, respectively. Microscopic examination of conidia at 40X magnification revealed variability in conidia size and could be categorized into two groups that, is small (<40 μm) and long (>40 μm) which is not depend on their geographical origin.

### Pathogenicity test

All the isolates were tested for their pathogenicity on tomato, mustard, brinjal and potato plants following Koch’s postulates. It was observed that a greater degree of variation exist between host plants. However, the maximum pathogenic effect was recorded in case of tomato (*Lycopersicon esculentum*). The symptoms of the disease as observed in selected host plants were characterized by black sunken necrotic lesions having typical concentric rings increasing gradually and covering maximum area of the leaves when compared to untreated control samples. A comparison was made between all the sample host plants and it was interpreted that the pathogen causes maximum damage to tomato plants as it covers almost 75–80% area of the whole leaf affected within 4–5 days followed by mustard, potato and lastly brinjal indicating the choice for host specificity for *Alternaria* pathogen. The pathogenic effect was found to be prevalent and significant in case of tomato plants hence, the tomato plant was chosen for further study. The *Alternaria* isolates examined had differences in the disease severity on test tomato (CO-3) variety. Based on the mean disease severity (MDS), the virulence of each isolate was recorded as low (MDS: 10–30%), moderate (MDS: 30–60%) or high (MDS: 60–100%). The isolates were thus categorized into 3 groups viz., highly pathogenic (13 isolates), moderately pathogenic (17 isolates), less pathogenic (18 isolates) based on the symptomatological variations in the test tomato variety (Table 1). The uninoculated tomato plants leaves showed no symptoms. All *Alternaria* isolates were successfully reisolated from disease-affected plants, thereby completing Koch’s postulates.

### ITS sequence analysis

The ITS region was successfully amplified from DNA from all *Alternaria* isolates in the study by the fungal-specific universal primer pairs ITS1 (forward) and ITS4 (reverse). The lengths of sequences as determined by capillary electrophoresis ranged from 500 bp to 600 bp (Supplementary Fig. S2). The obtained rDNA sequences were analyzed using NCBI-BLAST. The BLAST analysis of the ITS rDNA sequence data, supported the morphological identification, whereby the closest match (99–100% similarity) in the NCBI GenBank database was found to be with different *Alternaria* species. The ITS rDNA sequences of the 48 isolates have been deposited in the NCBI GenBank database (GenBank Accession numbers were given in Table 1). The BLAST results confirmed the different species of *Alternaria* and the alignment of the sequences was done using CLC Sequence Viewer 6.8.2. (Supplementary Fig. S2).

**Figure 2:**
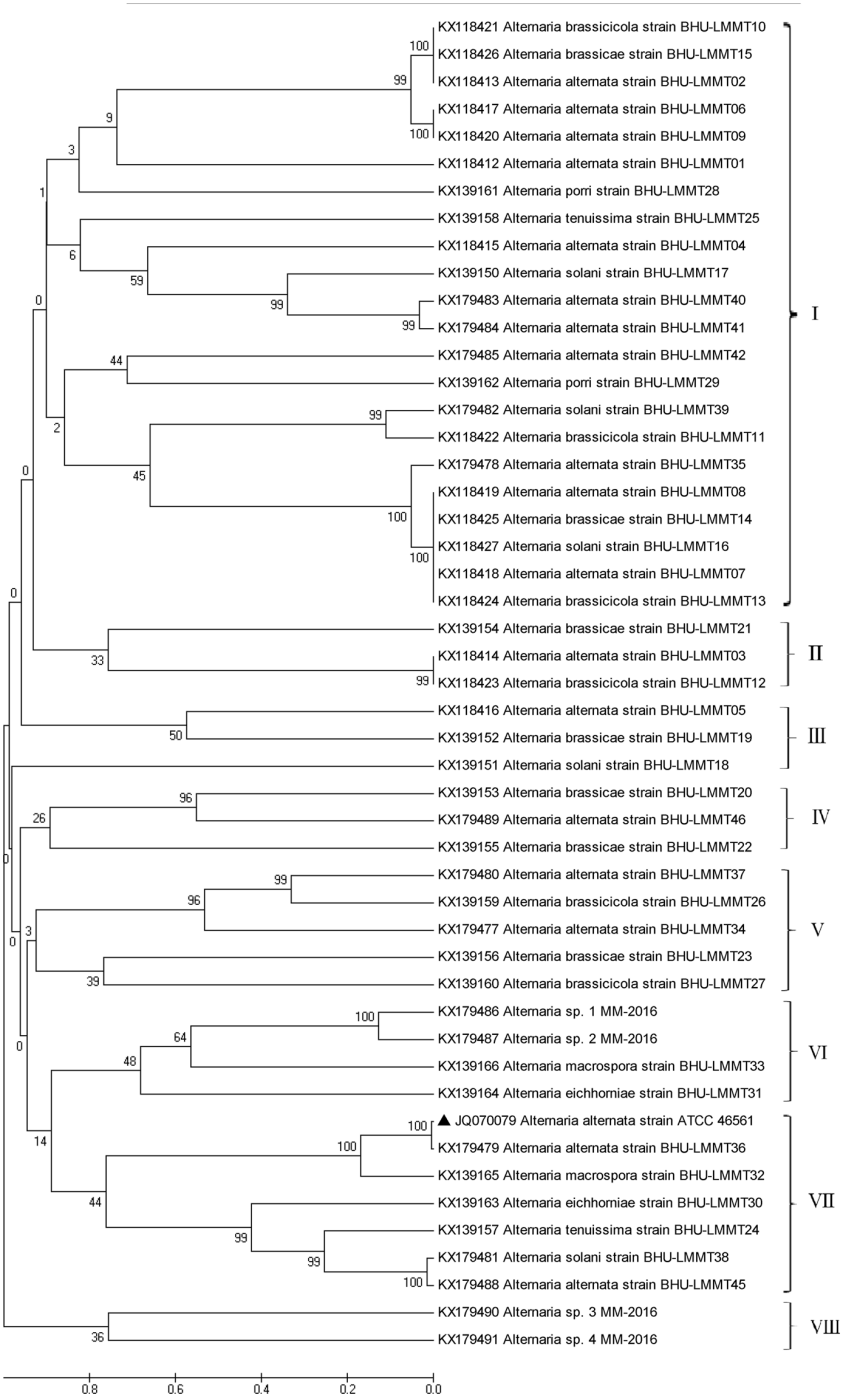
Phylogenetic relationship based on 18S rDNA sequences of different isolated *Alternaria* species. DNA sequences from the NCBI nucleotide database were aligned using the Clustal W program in MEGA 5.0, and constructed using the Neighbour-Joining method with 500 bootstrap replicates. The scale bar indicates the number of differences in nucleotide substitutions per sequences.

A dendrogram was constructed on the basis of ITS sequences using Clustal W and MEGA 5.0 software by Neighbour-Joining method (Fig. 2). The phylogenetic tree of genus *Alternaria* investigated in this study was clearly clustered into eight groups. The cluster I is the major group which contains *Alternaria alternata* as a dominant species (Fig. 2). Cluster III showed three species *Alternaria brassicae* strain BHU-LMMT21, *Alternaria alternata* strain BHU-LMMT03, and *Alternaria brassicicola* strain BHU-LMMT12 which were similar to each other. Since *Alternaria alternata* was found in all clusters in phylogenetic tree which showed polyphyletic nature of *Alternaria alternata*. Cluster VIII have two species of *Alternaria* i.e., *Alternaria* sp. 3 MM-2016 and *Alternaria* sp. 4 MM-2016, which was not supported by maximum bootstrap value it means theses two isolates (*Alternaria* sp. 3 MM-2016 and *Alternaria* sp. 4 MM-2016) might be new species.

### Thin layer chromatography analysis of *Alternaria* toxins

**Figure 3:**
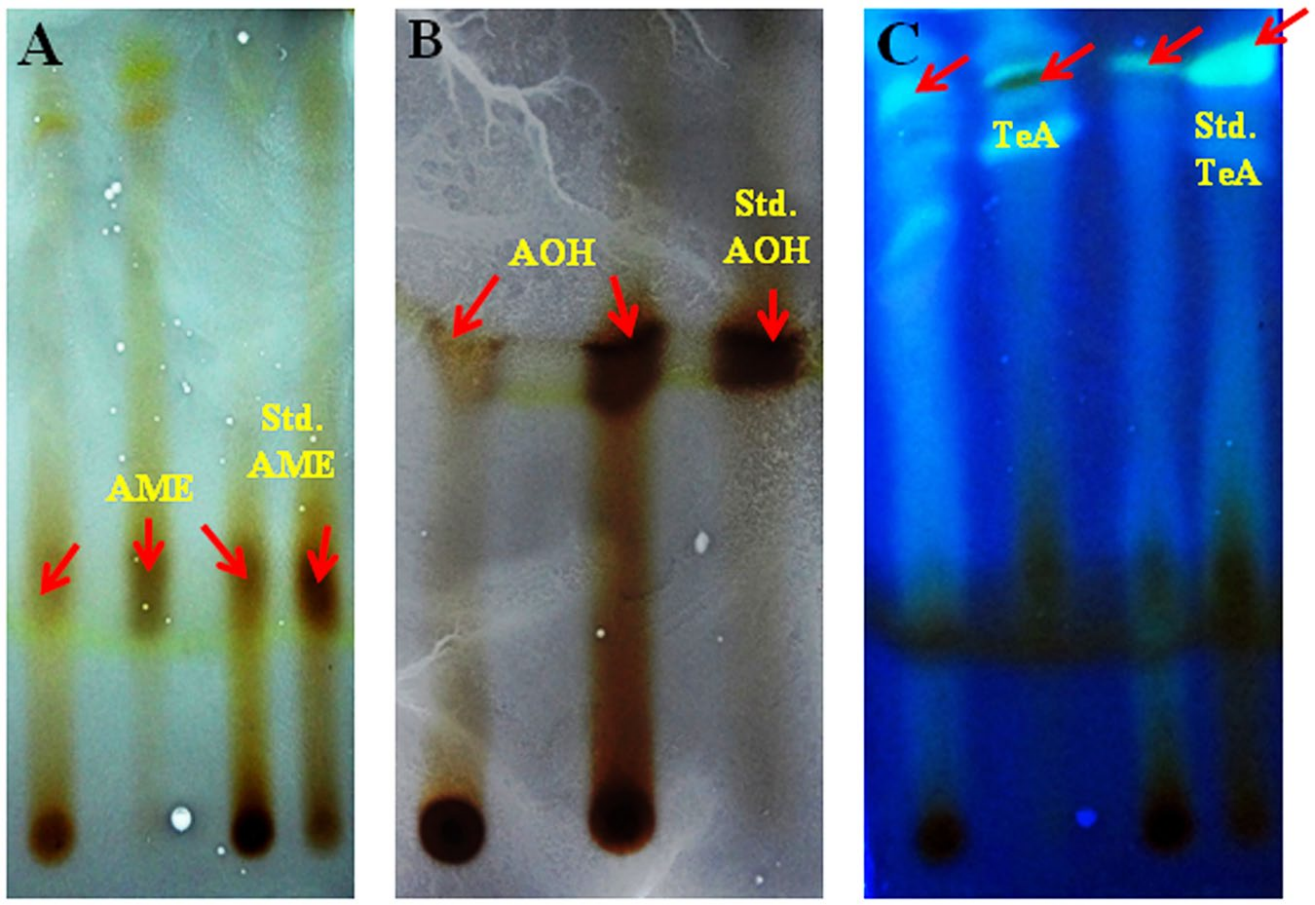
TLC analysis of different metabolites of *Alternaria* displaying different spots on TLC plates. (**A**) showing the spots of alternariol monomethyl ether (AME), (**B**) showing the spots of alternariol (AOH), and (**C**) showing the spots of tenuazonic acid (TeA), on the basis of their R_f_ values. The standards of these toxins were also run on similar plates for comparisons of the R_f_ values. The spots were visualized by spraying ferric chloride (FeCl_3_)  solution or under UV-light.

Thin layer chromatography (TLC) analysis was done for qualitative determination and characterization of mycotoxins recovered from culture filtrates of different isolates. A standard solution from identified toxin was used and underwent for TLC in order to determine the relevant spot which is well determined for particular toxin on the basis of their R_f_ values using different solvent systems (R_f_ values may differ in different solvents for particular metabolite). The culture filtrates from different isolates were also run for TLC to determine their respective spots similarly based on their R_f_ values (Fig. 3). The R_f_ values obtained from standard solution were then compared with the R_f_ values evaluated for separate metabolites. The preliminary identification was done on the basis of matched R_f_ values and which were further confirmed by the peaks corresponding to related standard compounds obtained through HPLC chromatogram. The R_f_ values determined for toxic metabolites from culture filtrates of different isolates as compared to standard TeA, AOH and AME on TLC using various solvent systems was presented in Table 3.

**Table 3:** Comparisons of R_f_ value of the phytotoxins isolated from *Alternaria* species with standards of tenuazonic acid, alternariol, alternariol monomethyl ether by TLC

R_f_ values	Chloroform: methanol (80:20)	Benzene: acetone: acetic acid (60:35:5)	Chloroform: methanol (95:5)	Ethyl acetate: benzene (95:5)
Standard TeA	0.63 ± 0.02	0.56 ± 0.03	0.42 ± 0.04	0.23 ± 0.05
Isolated toxins	0.62 ± 0.03	0.55 ± 0.01	0.41 ± 0.07	0.22 ± 0.06
Standard AOH	0.66 ± 0.09	0.59 ± 0.04	0.49 ± 0.03	0.31 ± 0.01
Isolated toxins	0.67 ± 0.04	0.60 ± 0.02	0.48 ± 0.04	0.32 ± 0.02
Standard AME	0.82 ± 0.01	0.78 ± 0.05	0.61 ± 0.09	0.39 ± 0.04
Isolated toxins	0.81 ± 0.06	0.77 ± 0.04	0.60 ± 0.02	0.38 ± 0.05

Note: R_f_ value is the ratio of the distance travelled by the substance and the distance travelled by the solvent (The spots were developed by spraying the plates with 0.2% FeCl_3_ in ethanol and some are detected in UV-light at 365 nm).

### High performance liquid chromatography analysis of *Alternaria* toxins

**Figure 4:**
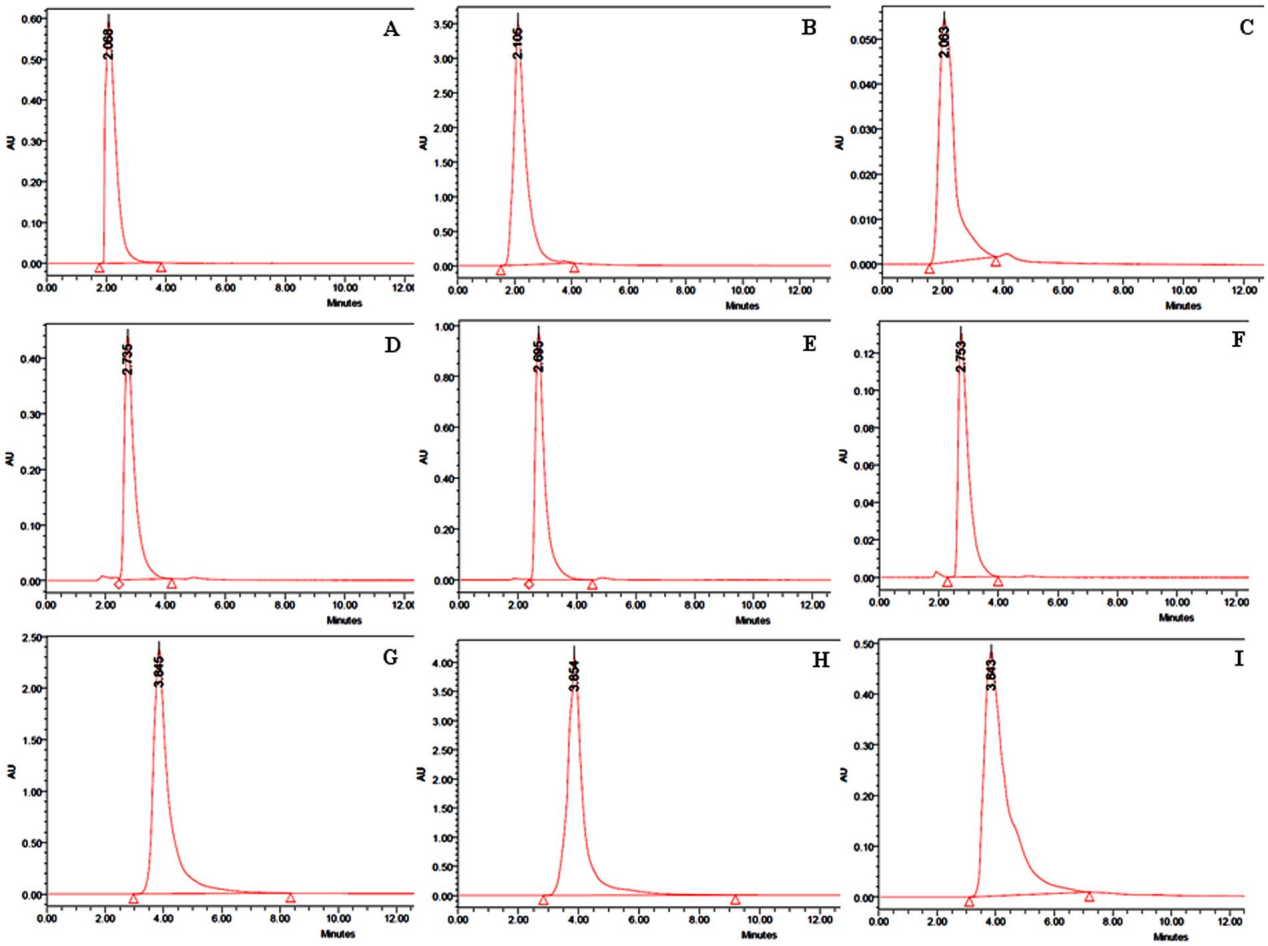
HPLC chromatogram representing the differential concentrations (maximum and minimum level) reported from toxigenic isolates of *Alternaria*. (**A**) standard chromatogram for TeA, (**B**) maximum conc. of TeA as recorded form isolate TM 4, (**C**) minimum conc. of TeA as recorded from isolate PE 1, (**D**) standard chromatogram for AOH, (**E**) maximum conc. of AOH from isolate TM 4, (**F**) maximum conc. of AOH from isolate ON 1, (**G**) standard chromatogram for AME, (**H**) maximum conc. of AME from isolate TM 4, and  (**I**) minimum conc. of AME from BJ 6. Note: The names of the *Alternaria* isolates were given as abbreviation of the different plants from which they were isolated (details described in Table 1).

The HPLC analysis of the standard metabolites TeA, AOH and AME showed specific retention time (RT) which were 2.07, 2.73, and 3.85 min, respectively (Fig. 4). The spots of different metabolites whose RT values were found similar with the RT values of the standards clearly demonstrated that the metabolites were same as the standards (Supplementary Fig. S4). The concentrations of the metabolites were determined on the basis of peak area. The UV absorbance spectra of isolated TeA, AOH and AME are shown in Supplementary Fig. S4B,C,E,F,H and I respectively. The absorption peak at 239.6 nm and 278.7 nm (TeA), 256.1 nm, 288.2 nm and 337.0 nm (AOH) and 240.8 nm, 283.4 nm and 327.5 nm (AME) are clearly observed. These results were found to be similar with UV-absorbance spectra of standards TeA, AOH and AME are shown in Supplementary Fig. S4A,D and G, respectively. This indicated that the TeA, AOH and AME were present into the analyzed samples.

The linear relationship between the detector response and different concentrations of toxins was obtained for all three *Alternaria* toxins (coefficient *r* ^2^ > 0.99 for all standard curves) (Supplementary Fig. S5 and Supplementary Table S1). The limits of detection (LOD) and limits of quantification (LOQ) for all each toxins of *Alternaria* were described in Supplementary Table S1. LODs and LOQs for each toxin were calculated as 28.42 and 86.13 μg/ml for TeA, 28.03 and 84.94 μg/ml for AOH and 12.66 and 38.36 μg/ml for AME.

A total of 48 *Alternaria* strains were analyzed for mycotoxin production. All of tested strains were able to produce at least one mycotoxin. All these strains produced TeA (75%), AOH (81.25%) and AME (95.83%) (Table 4). Out of 48 isolates, TM 4 (tomato leaf) isolate was found to have maximum concentration (TeA, 80.60 µg/ml; AOH, 125.28 µg/ml; AME, 106.45 µg/ml) of all the three metabolites. Three isolates viz. PE 1 (0.06 µg/ml), CR 2 (1.84 µg/ml) and BJ 5 (1.97 µg/ml) showed minimum concentration of TeA, AOH, and AME respectively.

**Table 4:** Table showed the concentrations of three different toxins tenuazonic acid (TeA), alternariol (AOH), and alternariol monomethyl ether (AME) from *Alternaria* isolates

Isolates	Concentrations of the toxins produced by different strains of *Alternaria* (µg/ml)								
	Tenuazonic acid	% RSD	% Recovery	Alternariol	% RSD	% Recovery	Alternariol monomethyl ether	% RSD	% Recovery
TM 1	2.52 ± 0.99	39.29	25	4.26 ± 0.99	23.24	42	9.96 ± 1.24	12.45	72
TM 2	8.69 ± 1.34	15.42	86	18.04 ± 3.88	21.51	36	24.44 ± 3.45	14.12	48
TM 3	2.24 ± 0.43	19.20	22	3.12 ± 0.67	21.47	31	9.59 ± 1.23	12.83	80
TM 4	80.60 ± 6.54	8.11	80	125.28 ± 10.99	8.77	63	106.45 ± 10.43	9.80	69
TM 5	6.34 ± 1.65	26.03	63	14.64 ± 2.96	20.22	67	36.48 ± 3.10	8.50	74
TM 6	9.53 ± 1.98	20.78	95	21.04 ± 3.95	18.77	42	29.77 ± 2.96	9.94	65
TM 7	51.17 ± 5.65	11.04	51	75.60 ± 8.94	11.83	76	104.60 ± 10.34	9.89	66
TM 8	47.46 ± 5.06	10.66	47	74.38 ± 8.34	11.21	80	88.73 ± 8.98	10.12	46
TM 9	6.91 ± 1.43	20.69	69	18.04 ± 3.56	19.73	56	28.17 ± 2.15	7.63	56
TM 10	40.32 ± 4.65	11.53	40	68.60 ± 7.54	10.99	62	72.15 ± 7.24	10.03	62
TM 11	62.96 ± 6.21	9.86	62	82.64 ± 9.53	11.53	80	101.16 ± 10.00	9.89	81
BJ 1	1.66 ± 0.12	7.23	16	ND	—	—	4.47 ± 0.45	10.07	44
BJ 2	ND	—	—	ND	—	—	3.62 ± 0.34	9.39	36
BJ 3	ND	—	—	4.98 ± 0.99	19.88	45	4.52 ± 0.47	10.40	45
BJ 4	1.32 ± 0.02	1.52	13	ND	—	—	3.67 ± 0.37	10.08	38
BJ 5	0.80 ± 0.01	1.25	80	7.84 ± 1.21	15.43	56	1.97 ± 0.01	0.51	49
BJ 6	ND	—	—	2.54 ± 0.02	0.79	39	1.92 ± 0.01	0.52	47
MT 1	8.69 ± 1.23	14.15	86	ND	—	—	43.74 ± 4.23	9.67	60
MT 2	3.24 ± 1.02	31.48	32	8.16 ± 1.54	18.87	61	11.93 ± 1.19	9.97	54
MT 3	2.74 ± 0.21	7.66	27	6.42 ± 1.23	19.16	39	10.75 ± 1.42	13.21	21
MT 4	33.49 ± 3.43	10.24	66	64.34 ± 6.78	10.54	29	71.47 ± 7.34	10.27	64
MT 5	2.78 ± 0.45	16.19	27	6.99 ± 1.05	15.02	44	14.53 ± 1.82	12.53	22
MT 6	3.88 ± 1.01	26.03	38	7.94 ± 1.45	18.26	67	18.16 ± 1.82	10.02	55
MT 7	30.85 ± 3.23	10.47	60	58.22 ± 5.67	9.74	34	69.52 ± 6.23	8.96	78
MT 8	20.75 ± 2.98	14.36	40	47.76 ± 4.56	9.55	18	63.05 ± 6.09	9.66	74
PT 1	2.58 ± 0.43	16.67	25	4.87 ± 1.24	25.46	55	5.19 ± 0.56	10.79	20
PT 2	14.98 ± 1.43	9.55	28	26.43 ± 4.52	17.10	46	46.18 ± 4.12	8.92	63
PT 3	18.56 ± 2.65	14.28	37	49.76 ± 5.67	11.39	33	59.733 ± 5.92	9.91	71
CF 1	12.19 ± 2.43	19.93	24	25.22 ± 3.45	13.68	39	44.78 ± 3.92	8.75	56
CF 2	4.20 ± 0.88	20.95	42	ND	—	—	3.43 ± 3.45	100.58	35
CF 3	16.38 ± 2.67	16.30	32	37.70 ± 4.23	11.22	34	47.23 ± 4.65	9.85	72
PE 1	0.06 ± 0.01	16.67	6	ND	—	—	2.87 ± 0.08	2.79	29
PE 2	ND	—	—	3.42 ± 0.76	22.22	35	ND	—	—
PE 3	1.20 ± 0.00	68.33	12	5.73 ± 0.78	13.61	26	3.32 ± 0.03	0.90	66
CA 1	5.33 ± 0.10	1.88	53	12.95 ± 1.27	9.81	19	18.56 ± 1.72	9.27	41
CA 2	4.24 ± 0.10	2.36	74	10.62 ± 1.05	9.89	21	18.33 ± 1.71	9.33	57
SP 1	ND	—	—	ND	—	—	8.65 ± 0.82	9.48	62
SP 2	ND	—	—	6.75 ± 0.78	11.56	27	12.65 ± 1.00	7.91	54
ON 1	ND	—	—	2.43 ± 0.74	30.45	15	9.87 ± 0.98	9.93	71
ON 2	ND	—	—	ND	—	—	13.27 ± 1.88	14.17	42
CR 1	ND	—	—	3.23 ± 0.03	0.93	34	7.25 ± 0.72	9.93	72
CR 2	ND	—	—	1.84 ± 0.02	1.09	18	6.62 ± 0.56	8.46	66
EC 1	5.69 ± 0.10	1.76	—	12.31 ± 1.32	10.72	34	19.50 ± 1.76	9.03	55
EC 2	5.87 ± 0.02	0.34	58	12.35 ± 1.56	12.63	38	19.75 ± 1.97	9.97	44
LT 1	ND	—	—	15.24 ± 1.98	12.99	33	ND	—	—
LT 2	ND	—	—	13.23 ± 2.09	15.80	45	5.63 ± 5.24	93.07	56
PR 1	5.87 ± 0.02	0.34	58	ND	—	—	23.12 ± 0.02	0.09	68
PR 2	6.01 ± 0.03	3.56	61	14.29 ± 2.96	20.71	55	23.99 ± 2.98	12.42	65

Note: ND - Not detected. Mean values ± standard deviation of all the experiments consisting of three replicates each.

### LC-MS/MS analysis of isolated toxins

**Figure 5:**
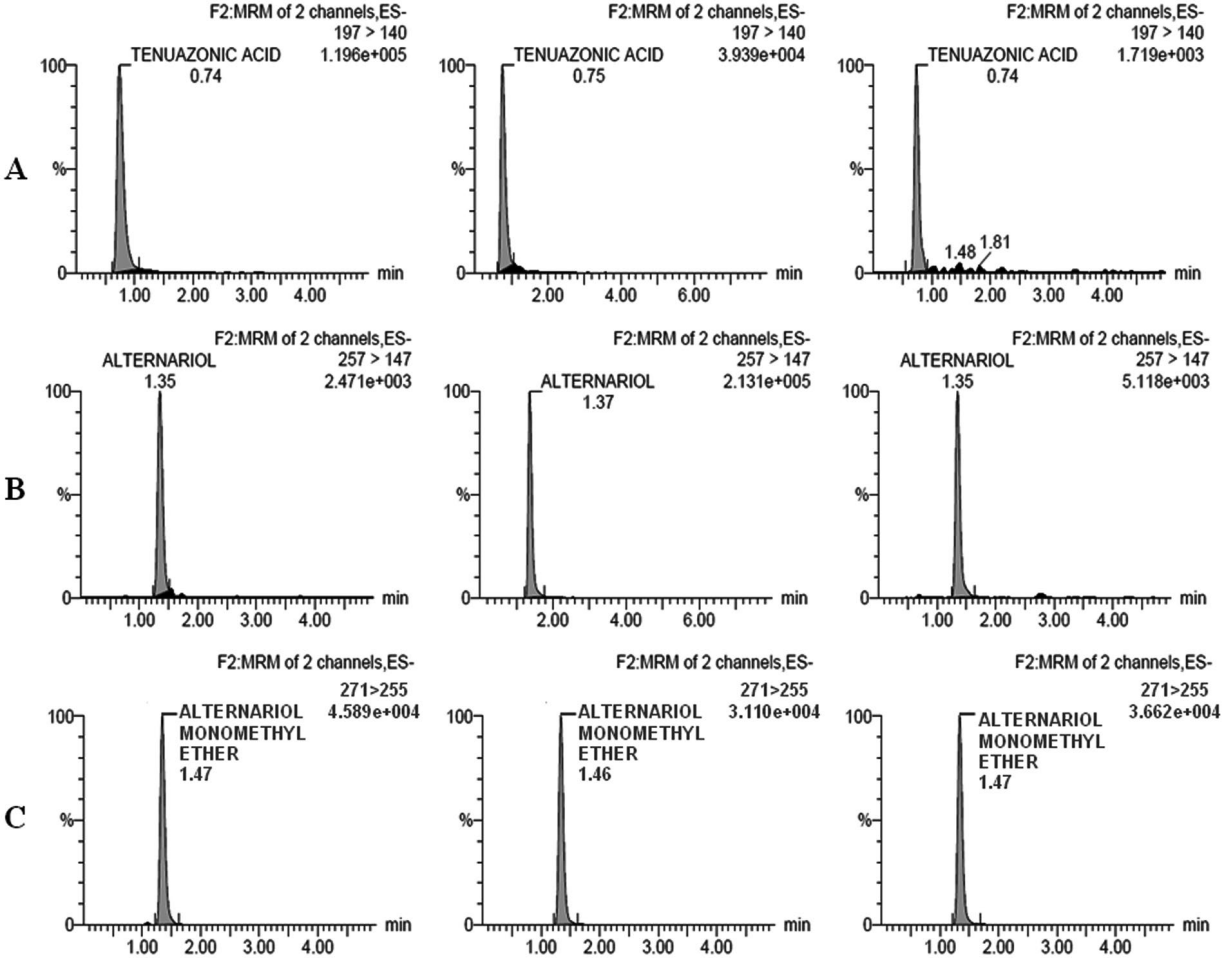
LC-MS/MS analysis (using the Accucore RP-MS 100 × 3, ACQ-TQD, QBP 1152) of TeA, AOH and AME in different isolates of *Alternaria* species which showed higher amount of these toxins. (**A**) Chromatograms of TeA, Collision energies for TeA (20 eV) and multiple reaction monitoring (MRM) transitions (ES+198/125 and ES−197/140). (**B**) Chromatograms of AOH, Collision energies for AOH (30 eV) and multiple reaction monitoring (MRM) transitions (ES+259/185 and ES−257/147). **(C)** Chromatograms of AME, Collision energies for AME (32 eV) and multiple reaction monitoring (MRM) transitions (ES+271/255 and ES−271/228).

LC-MS/MS analyses were done by the method of target compound analysis. The results showed confirmation of *Alternaria* toxins on the basis of mass/charge ratio, which were different for all three toxins. The *Alternaria* toxins details were given in Table 5 which detected in LC-MS/MS results. LC-MS/MS analysis of TeA, AOH and AME showed retention time (RT) 0.74–0.75, 1.35–1.37, and 1.46–1.47, respectively. The chromatogram peaks confirm the presence of TeA, AOH and AME toxins in the isolated samples (Fig. 5A,B,C).

**Table 5:** MS/MS ion transitions, settings and ion ratio on Accucore RP-MS 100 × 3, 2.6 µm, ACQ-TQD-QBB1152 instrument.

Compounds (MRM Data)	Detector time segment (min)	Product ions (m/z)	Cone/tube lens voltage (V)	Collision energies (eV)	Dwell time (sec)	Delay time (sec)	Wavelength (nm)	Pressure limit (psi)
ES+								
AME	1.00–1.50	271.0/255.0	20	25	0.048	Auto	200–450	0–15000
AOH	1.00–2.00	259.0/185.0	30	30	0.048	Auto	200–450	0–15000
TeA	1.00–2.00	198.0/125.0	30	20	0.048	Auto	200–450	0–15000
ES−								
AME	1.00–1.50	271.0/288.0	20	32	0.048	Auto	200–450	0–15000
AOH	1.00–2.00	257.0/147.0	30	30	0.048	Auto	200–450	0–15000
TeA	1.00–2.00	197.0/140.0	30	20	0.048	Auto	200–450	0–15000

Note: Ion mode: ESI; Ion polarity: positive and negative; AME, alternariol monomethyl ether; AOH, alternariol; TeA, tenuazonic acid.

### Comparisons of the amount of mycotoxins (TeA, AOH, and AME) in isolated *Alternaria* species

**Figure 6:**
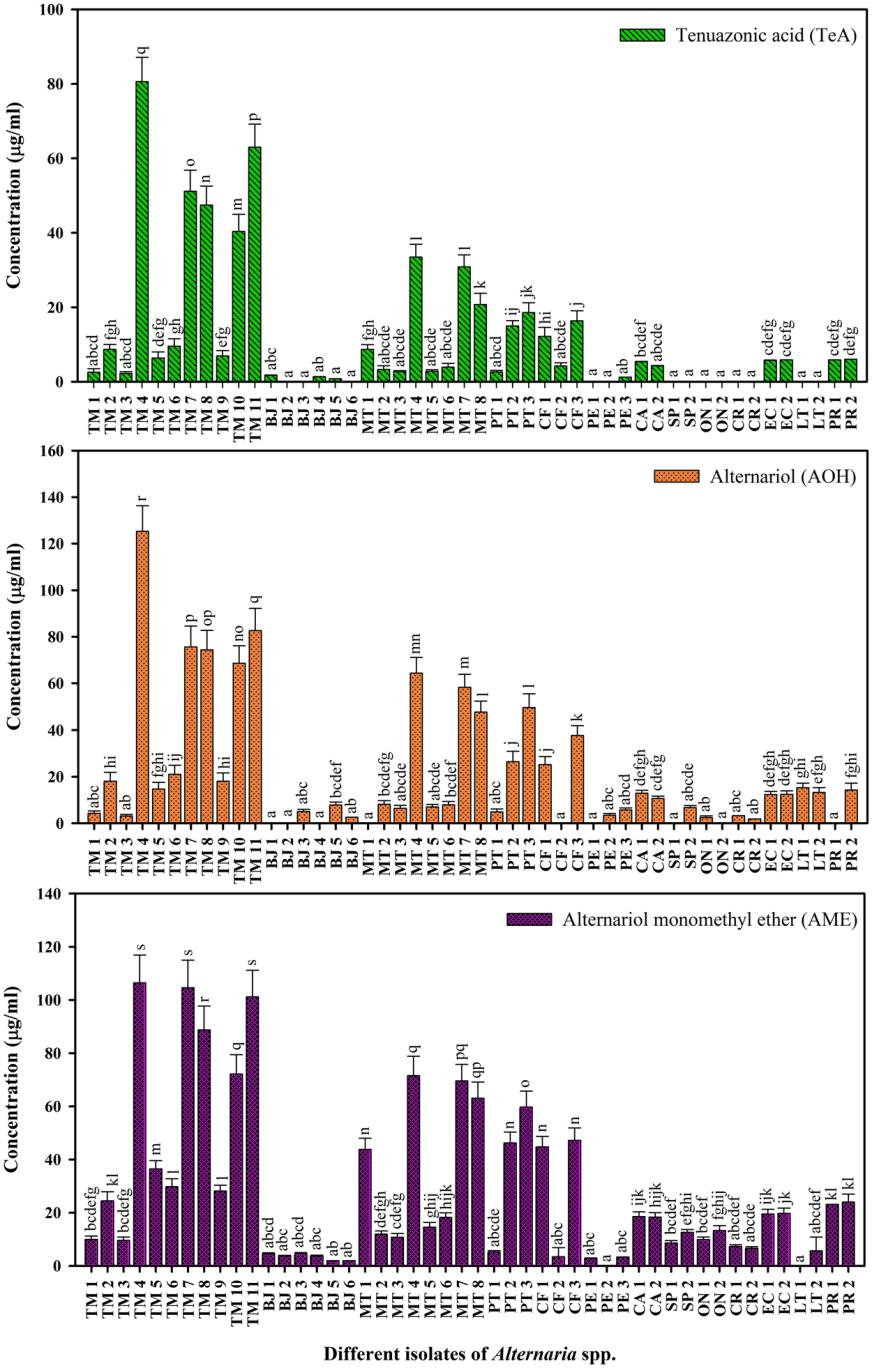
Graphical representation of different mycotoxins (TeA, AOH and AME) and their varying concentrations from 48 selected pathogenic isolates of *Alternaria*. Results are expressed in mean of three replicates and vertical bars show the ± SD of the mean.

The comparisons of three metabolites (TeA, AOH, and AME) from 48 pathogenic isolates of *Alternaria* species were statistically analysed by Duncan’s multiple range test at *P* ≤ 0.05 was shown in (Fig. 6). The result clearly indicates that the isolate TM 4 (tomato leaf) significantly produces high concentration of all the three toxins as compared to other isolates. TeA was not found in some *Alternaria* isolates such as BJ 3, BJ 6, SP 2, ON 1, CR 1, CR 2, LT 1, and LT 2. In the same way, AOH was also not found in other *Alternaria* isolates such as BJ 1, BJ 4, MT 1, CF 2, PE 1, and PR 1, while AME was absent in LT 1. TeA and AOH both toxins were not found in BJ 2, SP 1 and ON 2, whereas TeA and AME both toxins were also not found in PE 2 (Fig. 6).

### Assessment of efficiency of toxicological effects of different mycotoxins (TeA, AOH and AME)

**Figure 7:**
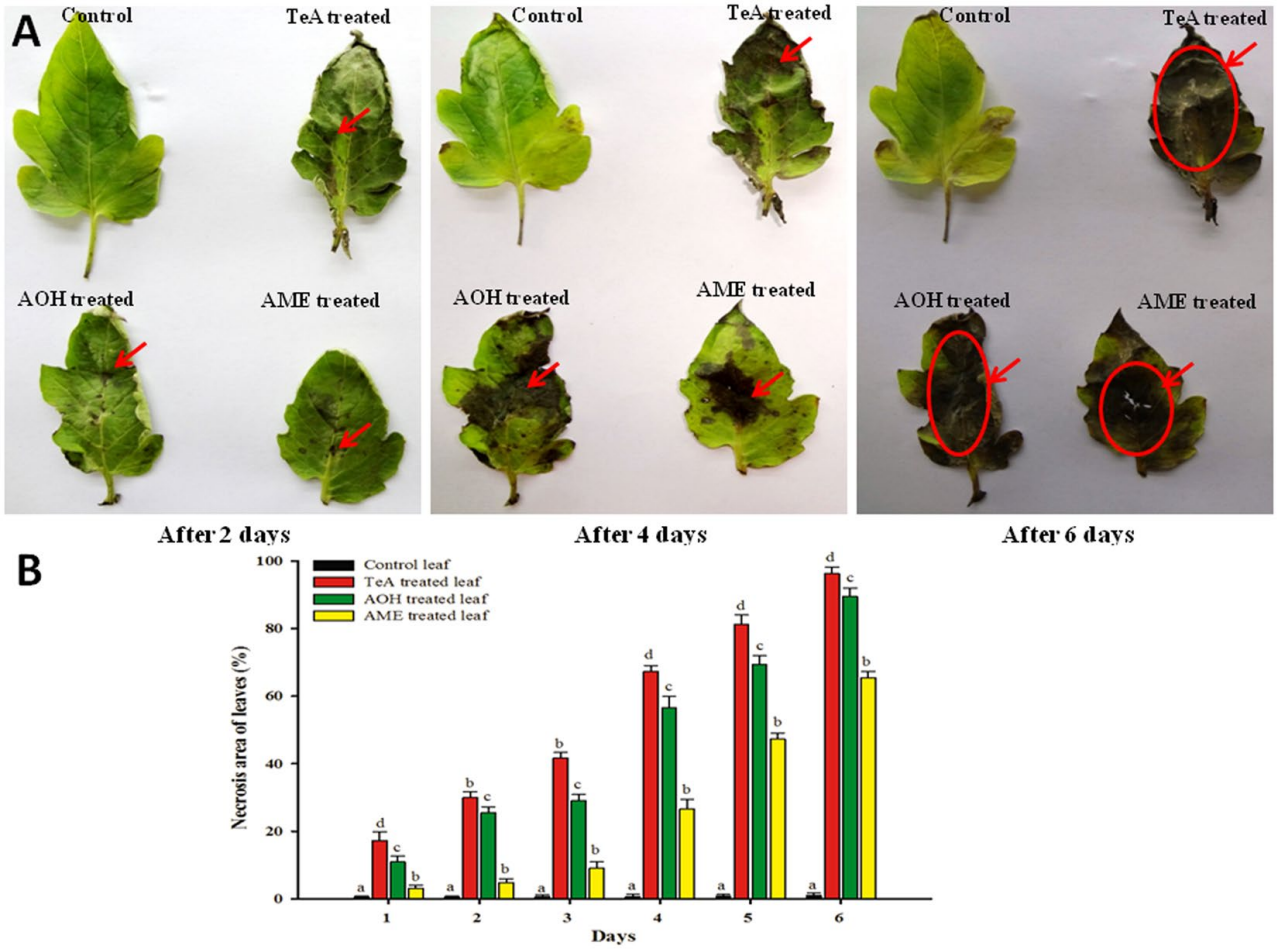
Comparision of toxic potency of TeA, AOH and AME on leaves of tomato plants. (**A**) Necrotic spots (diseased area) developed after 2, 4 and 6 days of mycotoxins inoculum treatment. (**B**) Graphical representation of percent diseased area (necrotic regions) and days after treatment of mycotoxins (results are expressed in mean of three replicates and vertical bars show the ± SD of the mean). Note: The arrows indicate the necrotic symptoms of diseased area.

The toxic effect induced by each of the three mycotoxins differs among different isolates and a remarkable difference has been observed in the efficiency of separate mycotoxins in causing cell damages and cell death at varying time interval. Three toxins namely TeA, AOH and AME were given separate treatment on different leaf samples of tomato plants. The necrotic region developed clearly indicates the extent of damages caused by these toxins. The extent of tissue damages was measured or expressed in the form of percentage area covered under necrotic regions during the time interval. The results obtained through this study indicates that TeA caused maximum damages and contributes maximum percentage to cell damages at all intervals of time followed by AOH and lastly AME when compared to control leaf samples having almost no tissue damages. The graphical representation as showed in Fig. 7 represents the effect of different mycotoxins at different interval of time. TeA covers maximum area of cell death from all interval of time from day 1 (30.0%) to day 6 (96.36%) followed by AOH covering an area (25.45%) on day 1 to (89.44%) day 6 and least toxic effect was imposed by AME having percentage coverage area (4.84%) on day 1 to (65.45%) day 6. Therefore, it can be easily interpreted that the potential of TeA among different toxins secreted by *Alternaria* is higher in developing necrotic spots hence cell death.

### Cell death determination

**Figure 8:**
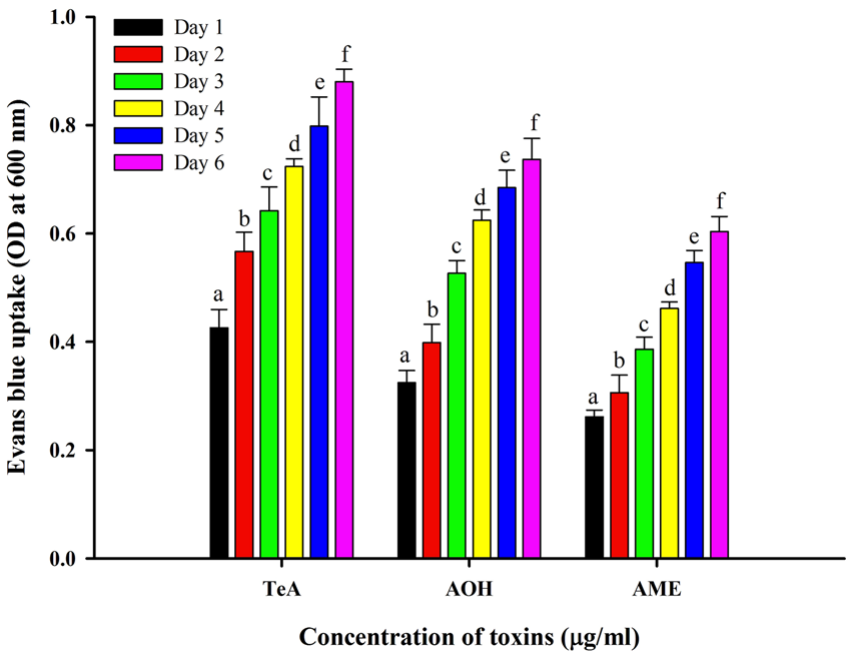
Cell death assay by uptake of Evans blue stain in the leaves of tomato plant treated with *Alternaria* toxins (AME, AOH and TeA) at different days intervals. The results are expressed as mean of three replicates and the vertical bars showed the ± SD of the mean. Different letters indicate that the values are significantly different from each other (*P* ≤ 0.05).

Further, cell death and cell damages were also determined by Evans blue uptake assay. The cell death was observed to increase with the time interval after given the toxins treatment (TeA, AOH and AME) of leaves. The results showed that more cell death was observed in TeA treated leaves (0.88 Evans blue uptake) followed by AOH (0.74) and AME (0.60) treated leaves (Fig. 8). The maximum cell death observed at 6 day in all the treatment. The highest cell death was found in TeA treated leaves samples (2.06-fold higher) at the day 6 as compared to day 1. Similarly, AOH, AME treated leaves found (2.27-fold and 2.30-fold higher, respectively) at the day 6 as compared to day 1.

## Discussion

*Alternaria* species are known as major plant pathogens and belongs to a diverse and ubiquitous group of fungi. In the present study, remarkable differences in morphological characteristics have been found such as colony morphology, conidial septation pattern and, shape and size of conidia among the different isolates of *Alternaria* collected from different regions in India. These morphological variations may be caused by alteration in temperature and their geographical regions^40, 41^. The fungal pathogens were isolated from ideal environmental conditions including moist weather conditions with temperature ranging between 6–26 °C and soil pH 3–6.5. As the most favourable condition supporting the growth of *Alternaria* includes an optimal temperature ranging from 25–32 °C and at pH 4–5^42, 43^. The present studies investigated the efficiency of different isolates of *Alternaria* species and their differential potential to infect different hosts in terms of affected leaf area. The maximum pathogenicity was observed in tomato plants in comparison to other host plants. However, quality and quantity of different mycotoxins are the key factor for the detection of pathogenicity among different isolates.

During their active growth and reproduction a wide array of toxic metabolites, including mycotoxins bearing different groups and complexity like dibenzopyrones, tetramic acids, lactones, quinones and cyclic peptides have been identified in *Alternaria* species^3, 25, 44^. It has been reported that besides these secondary metabolites some other chemical entities can be used for species identification^45, 46^. Of these various metabolites mycotoxins are of particular importance causing acute toxicity by damaging cell components of actively growing cells during the course of symptom development in their hosts^47, 48^. It has been demonstrated through several studies the reliability of these fungal metabolites in metabolic profiling for remarking differences and characterizing different pathogenic groups. The implication of these metabolites and characterizing these isolates through several analytical techniques like HPLC, MALDI-TOF MS and TLC has been experimentally reported^49–51^. Out of several mycotoxins reported from *Alternaria* the characterization of two potent toxins, AOH and AME have been evaluated on the basis of their retention factor values in presence of different solvents using TLC^52^. We have demonstrated and confirmed the presence of three mycotoxins including TeA, AOH and AME through TLC (based on R_f_ values) and HPLC (based on retention time). Additionally the HPLC studies assist in quantitative determination of these metabolites using standard, available for these mycotoxins. The results showed the presence of higher dosages of these mycotoxins in 13 isolates out of total 48 isolates studied and have maximum pathogenic (60–100%) potential when compared to those isolates having moderate (30–60%) or low pathogenic (10–30%) (Table 1).

TeA was isolated from *Alternaria longiceps*, *Alternaria kikuchiana*, *Alternaria mali* and *Pyricularia oryzae* as a phytotoxin and from *Alternaria* *alternata*, *Alternaria tenuissima* and *Phoma* sorghina as a mycotoxin^22, 28, 44^. Several studies demonstrated that various isolates of *Alternaria* species were detected TeA and reported its wide-spread occurrence^46, 51, 53^. Thus, it was thought to be a characteristic metabolite of this genus and not a pathogen-specific toxin. Several other workers also observed the production of TeA from *Alternaria* species isolated from different host plants^6^. It is considered to be having highest toxicity amongst the mycotoxins produced by *Alternaria* ^31, 54^. Our results also revealed that out of 48 strains of *Alternaria* species isolated from different vegetables, crops, and weed plants and 36 isolates produced TeA in maximum amount (Table 4), other than AOH and AME, which are host specific toxins. EFSA^55^ has also reported that TeA has  highest contamination frequency in tomato products. AOH, AME are cytotoxic and show synergetic effects^23^. *A. alternata* toxins influenced carrot seed germination negatively and TeA processes negative effect on carrot seed germination^56^.

We have also demonstrated the level of pathogenicity of different isolates recovered from different plant samples and their efficiency for mycotoxin production in tomato plant. The result indicates the level of three different mycotoxins and their concentration represented in µg/ml, produced during their active growth. The statistical investigation also reveals the effect of *Alternaria* mycotoxins and their susceptibility on the host plant. The most drastic effect of pathogenicity of fungal genus has been found to be maximum in tomato as the potential concentration of all mycotoxins. It has been found that maximum isolates recovered from different tomato plants showed different toxic levels of all the mycotoxins when compared to their different hosts. The isolate TM 4 showed maximum effective concentration for all the three mycotoxins viz., TeA, AOH and AME. The other isolates recovered from different host have low level for all the mycotoxins. The study clearly demarcates the mycotoxic effect of *Alternaria* over tomato leaves and their potential for causing disease. The difference and variation found for different mycotoxins is due to defense mechanism responded by plants or to some extent their resistance against the pathogen and disease.

## Conclusion

The present study reports the molecular characterization, pathogenicity and toxigenicity of *Alternaria alternata* isolates from different regions of India and their relationship with other formae specilaes within the species of *Alternaria*. This study concludes that the fungal genus *Alternaria* encompasses severe pathogenic species causing diseases to various economically important horticultural crops and vegetables in Indian subcontinent. The study demonstrates the more prevalent pathogenic effect of *Alternaria* isolates over tomato when compared to other host plants and directly correlates the specificity of the pathogen for its host with regard to infection and disease development. The study also indicates that analyzing the genetic variability among *Alternaria* strains would be of great importance in plant breeding for disease resistance and can be used by plant breeders. The present study confirms that the more pathogenic isolates of *Alternaria* TM 4 (isolated from tomato leaf) secretes maximum amount of mycotoxins as compared to other isolates, which deteriorates the host plant and reduces the quality and significance of tomato plants. The isolated mycotoxins including TeA, AOH and AME from infected plants explain the role of these mycotoxins in producing necrosis in plants and appearance of diseased condition. However more research is needed to be done in this field to determine the effect of these toxins on animal model for future developments and well-being.

## Material and Methods

### Collection and isolation of *Alternaria* species

The infected parts such as leaves, fruits and stems of diseased plants from different regions in India, were collected and brought in the laboratory. The leaf samples were surface sterilized with 0.5% sodium hypochlorite solution, washed thoroughly with sterile distilled water (SDW) for several times and were placed on potato dextrose agar (PDA) culture medium in Petri dishes for 3–4 days. The PDA plates containing infected leaf pieces were incubated at 28 °C for 12 h light/dark photoperiod for 6–10 days^57^. To avoid bacterial contamination, streptomycin was supplemented in the medium. The conidia were produced single-sporulated to obtain pure colonies, which were placed onto sterilized filter paper.

### Pathogenicity Test

To determine the formae specials, virulence analysis of the isolates was carried out on tomato cultivars susceptible to *Alternaria*. A total of 60 isolates of *Alternaria* were tested for pathogenicity. Tomato seeds were scarified in sodium hypochlorite, rinsed in tap water, and then air dried. Plants were grown separately in pots. Physiological conditions such as temperature and humidity for plant growth were maintained at 28 °C to 32 °C and 40 to 60% relative humidity, respectively. Optimum inoculums concentration was maintained (2 × 10^6^ spores/ml) and sprayed on leaf area. Plants under experiment were maintained in dew chambers for 8 h at 25 °C. These plants were regularly monitored for 2–3 days for infection severity and disease development with respect to control leaf that was uninoculated. Symptoms started to be visible 1 day after spraying the spores inoculations. Disease severity was assessed from 1 day of inoculation up to 6 days. Data were statistically analyzed by using analysis of variance (ANOVA) and Duncan’s test (*P* ≤ 0.05).

### DNA extraction and identification of pathogen

The pathogen was initially identified on the basis of morphological characteristics including size and shape, and structure of conidia, and further confirmed by ITS amplification using universal primers ITS1 (5′-TCCGTAGGTGAACCTGCGG-3′) and ITS4 (5′-TCCTCCGCTTATTGATATGC-3′) amplifying ITS regions and 5.8S genes encoding for fungal species. DNA extraction was carried out as per the method suggested by Doyle and Doyle^58^. Lyophilized mycellar mat of 0.5 g was grinded in a mortar and pestle using 10 ml of CTAB extraction buffer and then incubated at 65 °C in water bath for 30 min. The sample was then mixed with an equal volume of chilled chloroform/isoamyl alcohol and gently mixed followed by centrifugation at 10,000 rpm for 10 min at 4 °C. The supernatant thus obtained was mixed with equal volume of isopropanol and left it for 2 h at 4 °C. The sample was again centrifuged at 10000 rpm for 10 min at 4 °C temperature. The pellet was then rinsed with 70% ethanol and air dried for 4 h in order to remove the traces of alcohol. Amplification ITS rDNA reaction were performed in 25 μl reaction mixture containing 2.5 μl 10X reaction buffer, 5 μl of each deoxyribonucleotide triphosphate (dNTP), and 1.0 μl each of ITS and 5.8 S region universal forward primer (ITS1) and reverse primer (ITS4), 0.3 μl of Taq DNA-polymerase, 10–100 ng DNA, and 2.5 μl MgCl_2_. The optimized thermal profile of PCR were initial denaturation at 95 °C for 3 min, denaturation at 95 °C for 30 sec, annealing at 70 °C for 30 sec and Final extension at 72 °C for 1 min with additional 40 cycles. The amplification were confirmed on 1% agarose gels in 0.5X TBE buffer, run parallel to standard DNA molecular weight marker and visualized under UV-transilluminator.

### ITS Sequence analysis

The obtained ITS rDNA regions of selected isolates were further cut down and purified using QIAquick PCR purification kit (QIAGEN, Germany), according to the manufacturer’s instructions. The purified products were finally sent to SciGenome Cochin, Kerala, India for sequencing. The sequences were compared to those in GenBank (http://www.ncbi.nlm.nih.gov/) using NCBI BLAST. The BLAST analysis was performed with full length ITS sequences as queries to reveal relationships to published sequences. Highest homology and total score were noted for further analysis. The sequences obtained in the present study were submitted to GenBank. The ITS sequences of *Alternaria* strains from other formae speciale was downloaded from the NCBI GenBank database and were used in the phylogenetic analyses as reference sequences. All the DNA sequences were aligned with the program Clustal W included in BioEdit sequence alignment editor^59, 60^. The resulting multiple-alignment file was used for phylogenetic analyses which were performed using MEGA 5.0 with Neighbor-Joining method allowing 500 bootstrap replicates^61^.

### Extraction of the toxins from *Alternaria* species

Extractions of the toxic metabolites were carried out according to the method of Andersen *et al*.^62^ with some modifications. The extractions of these phytotoxin were carried out on Potato Dextrose Broth (PDB) medium by using 20-day-old cultures. Three agar plugs (3 mm) were cut from the centre of each *Alternaria* colony and inoculated in 200 ml PDB medium. Colonies of the pathogen were cut with the help of cork borer (5 mm) and then colonies were inoculated in the PDB medium. Twenty day old cultures were filtered through filter paper by the vacuum filter machine. Added equal volume of methanol into the culture filtrates, mixed it properly and kept at 4 °C for 24 h. Thereafter, the filtrate was precipitated and was evaporated the methanol to dryness in a rotary vacuum concentrator (IKA^®^ RV 10) at 43 °C. An equal volume of ethyl acetate was added to the extracted filtrate, mixed properly in separatory funnel. Two phases were obtained, one was organic phase and another was aqueous phase. The aqueous layer was separated and extracted with ethyl acetate. The ethyl acetate extract was concentrated at 44 °C in vacuum evaporator and dissolved in methanol.

### Purification and separation of *Alternaria* phytotoxin via column chromatography

Purification and separation of compounds were performed using methodology of Devi *et al*.^63^ with slight modifications. Column chromatography (CC) was undertaken in a glass column (700 mm × 30 mm) and silica gel (100–120 mesh size Merk) was chosen for stationary phase. The mobile phase consisted of pure solvent or different solvents depending upon requirement of conditions. Column was loaded with crude complex extracted from isolates of *Alternaria* species. For separation of toxic metabolites mobile phase consisted of chloroform: methanol (80:20 and 95:05), benzene: acetone: acetic acid (60:35:05) ratio was used for separating compound and gradient elution was followed. Different fractions eluted from CC were separated by thin layer chromatography (TLC) and confirmed by the HPLC analysis.

### Thin layer chromatography (TLC) analysis of the phytotoxins

Thin layer chromatography (TLC) was used to identify the various phytotoxins produced by large as well as small spores of identified *Alternaria* species. TLC was performed by using the method of Andersen *et al*.^64^. In this method, 4.5 g silica gel G254 (13% CaSO_4_ ½ H_2_O as a binder) was added with 25 ml double distilled water and stirred by glass rod until slurry of silica gel was formed. After which the slurry was applied on glass plate gently and placed at safe place for air drying. Different mobile phases were used for the separation of various phytotoxins in the ratio of chloroform: methanol (80:20; v/v), benzene: acetone: acetic acid (60:35:5; v/v), chloroform: methanol (95:5; v/v), ethyl acetate: benzene (95:5; v/v). These solvent mixtures were then placed in desiccators and were left for 30 min to saturate the environment inside it. Before performing TLC, the glass plates coated with silica gel were charged by putting it in oven at 60 °C for 10 min. After charging, 5 µl samples were spotted at different point 2 cm away from base. After spotting the samples, the TLC plates were dried inside a desicator for few minutes. The resulting spots were developed by exposing the TLC plates to 0.2% ethanolic ferric chloride/or visualized under UV-light at 365 nm. TLC plates were then air-dried overnight, after which the R_f_ values were calculated. The spots of different metabolites whose R_f_ values were found similar with the R_f_ values of standards were scratched out, dissolved in HPLC grade methanol and used for HPLC analysis.

### HPLC-UV Analysis

#### Preparation of the standard

TeA (cat No: T1952), AOH (cat No: A4675) and AME (cat No: A4678) were purchased from Biogenuix (LKT laboratories, Inc., New Delhi, India) and used in crystallised form to prepare the standard. The stock solution (1000 µg ml^−1^) and a separate working solution of (10 µg ml^−1^) of toxins were prepared in HPLC grade methanol and kept at −20 °C for further use. These toxins were used as standards for HPLC calibration and for other additional experiments by diluting the prepared working solutions.

### HPLC-UV analysis conditions

For HPLC analysis the samples were chromatographically separated using a base deactivated (250 mm long × 4.6 mm, 5.0 µm particle size) C18 Waters Spherisorb, ODS2 column (product No: PSS831915, USA) which was connected to the guard column, Waters series system (Waters, Waters Corporation, Milford, USA) having UV-VIS detector (2998 PDA) and Waters 600E system controller. The 2998 PDA detector set at 254 nm as the integration wavelength. The samples were injected using a 10 μl loop of Waters 717plus autosampler (Waters Corporation, Milford, USA). The column and guard column were thermostatically controlled at 28 °C. The flow rate was 0.70 ml/min and mobile phase consisted of 75% HPLC grade methanol (solvent A), 25% of an aqueous solution (solvent B) of 0.1 M phosphate buffer [Na_2_HPO_4_ (1 M) 7.9 ml + NaH_2_PO_4_ (1 M) 92.1 ml] added 900 ml DW for 1 liter and pH 5.8 maintained by phosphoric acid. The instrument was run in a linear isocratic mode and the detection was monitored at the range of 200–400 nm. The reliability of the HPLC-method for analysis of AME, TeA and AOH was validated through limit of detection (LOD) and limit of quantification (LOQ).

### LC-MS/MS analysis

For further confirmation of the *Alternaria* toxins (AME, AOH and TeA), a chromatography – tandem mass spectrometric (LC-MS/MS) method has been carried out with slight modifications of Tölgyesi *et al*.^65^. The method involves a solid-liquid extraction with methanol and a subsequent derivatization for TeA, AOH and AME. Then, the samples were purified with solid-phase extraction on polymeric based cartridges, and finally, toxins were separated by LC-MS/MS. For LC-MS/MS analysis sample were prepared in step-wise process.

### Reagents, solvents and preparation of the standard

Dried-down analytical calibrants of AME, AOH and TeA were purchased from Biogenuix (LKT laboratories, Inc., New Delhi, India). Standards were reconstituted with 1.0 ml methanol to obtain 0.1 mg ml^−1^ stock solutions. All the stock solutions were kept at 4 °C. 2,4-dinitrophenylhydrazine (DNPH) and undecanal were purchased from Sigma-Aldrich. The derivatisation reagent (0.58% DNPH in HCl solution) was prepared as described by Siegel *et al*.^7^. The stop reagent was 5% (v/v) undecanal in methanol. The derivatised TeA, AOH and AME standard solutions (1.91 μg/ml, 2.54 μg/ml, and 2.71 μg/ml methanol, respectively) were prepared by mixing 1 ml of the 10 μg/ml methanolic TeA, AOH and AME solutions with 1 ml DNPH solution. The mixture was left overnight and processed as written in the sample extraction and SPE clean-up sections. The final volume was adjusted to 10 ml with methanol. These solutions were used to optimise the LC-MS/MS conditions for the analysis. A total of 50 mM ammonium formate buffer was prepared in water and its pH adjusted to 3.0 with formic acid. Methanol and acetonitrile were LC-MS grade obtained from Sigma-Aldrich. Ethyl acetate, n-hexane, dichloromethane, formic acid and ammonium formate were HPLC grade and purchased from Merck (Darmstadt, Germany). The Kinetex C-18 UPLC LG 500 column (3 × 100 mm, 2.6 μm), Strata SPE cartridges (6 ml, 200 mg) and regenerated cellulose (RC) syringe filters (15 mm, 0.45 μm) were obtained from Phenomenex (Utrecht, the Netherland). The Supelco Ascentis Express C-18, cyano (ES-CN) and phenyl-hexyl HPLC columns (2.1 × 100 mm, 2.7 μm) were purchased from Sigma- Aldrich. Standards toxins samples, used for method development, were purchased from Biogenuix (LKT laboratories, Inc., New Delhi, India). The samples were stored at −20 °C until subjected to analysis.

### Sample extraction

Crude metabolites extraction samples were purified by column chromatography method and the fraction were eluted and dissolved in methanol. 50 ml of sample from each fraction was mixed into 50 ml polypropylene (PP) centrifuge tubes which were then sealed. The samples were vortex-mixed for 5 sec and horizontally shaken on a CAT S50 shaker at 600 min^−1^ speed for 45 min at ambient temperature. Then, the tubes were centrifuged at 5,000 rpm for 10 min at 20 °C and the upper layer was collected in a new 50 ml PP centrifuge tube. 100 μl derivatization reagent (0.596% DNPH in 2 mol/lit HCl) was added to the sample and vortex-mixed for 5 sec. The sample was left to be derivatized for 1 h at ambient temperature. Afterwards, 500 μl stop reagent 5% (v/v) undecanal in methanol was added and vortex-mixed for 5 sec. The sample was left to stand for 30 min and then diluted in the PP tube up to 35 ml with 50 mM ammonium formate buffer (pH 4, adjusted with formic acid). The sample is centrifuged at 5,000 rpm for 10 min at 20 °C and subjected to solid-phase extraction clean-up.

### Solid-phase extraction (SPE) clean-up

Strata-XL (200 mg, 6 ml, 100 μm) cartridges were conditioned with 6 ml methanol followed by 6 ml water and 6 ml 50 mM formate buffer. 75 ml reservoirs were connected onto the cartridges and samples were loaded into the reservoirs. Then, the samples were passed drop wise. Afterwards, SPE columns are washed with 6 ml methanol–water (15/85, v/v) and subsequently with 6 ml n-hexane. The cartridges were vacuum dried for 5 min before eluting the samples into glass tubes with 5 ml methanol. The samples were evaporated to dryness at 45 °C under a gentle stream of nitrogen and they were re-dissolved in 250 μl methanol by vortex-mixing for 20 sec. As a final step, the samples were filtered through regenerated cellulose filters into HPLC vials.

### Instrumentation and equipment

The method development was carried out using an Ascentis Express C-18 (2.1 × 100 mm, 2.7 μm) UPLC LG 500 nm system (Accucore RP-MS 100 × 3.0 MM, 2.6UM, ACQ-TQD-QBB1152, Waters acuity PDA detector, Waters Corporation, Milford, MA, USA) coupled to a MassLynx triple quadrupole MS detector (Waters, Milford, MA, USA). Data acquisition and evaluation were performed with MassLynx version 4.0. The final method was also transferred to a Thermo ACQ-TQD Quantum Ultra LC-MS/MS system (Thermo Finnigan, San Jose, CA, USA) that involved a Waters acquity QSM binary pump (SN- L10QSM943A), a Waters acquity fin autosampler (SN-M10SDI443M), a column thermostat and a TQD Quantum Ultra triple quadrupole MS detector. Target column temperature and target sample temperature were 30 °C and 10 °C, respectively. Data acquisition and evaluation were performed using Xcalibur software 2.0.7. SP1. Both systems were equipped with an electrospray interface (ESI) in which negative ionisation alone was used during acquisition. Nitrogen was used as drying and collision gas. The ion source parameters are summarised in Supplementary Table S2. Further, the method transferability was investigated with an LC-MS/MS system that consisted of an Agilent 1100 HPLC coupled to an AB Sciex 4000 triple quadrupole MS (Framingham, MA, USA).

### Instrument conditions

The *Alternaria* toxins are separated on an Ascentis Express C-18 (2.1 × 100 mm, 2.7 μm) UPLC column equipped with a 2.1 mm C-18 pre-column using linear gradient elution. Four solvents (solvents A, B, C and D) were mixed by the binary pump. The solvent A contained; acetonitrile (ACN) + water (5:95), solvent B contained; ACN: 5% isopropyl alcohol (IPA), solvent C contained; 100% methanol and solvent D contained; pure ammonium acetate. The flow rate was 0.5 ml/min. The mobile phase of the solvents in initial time was 0.0% A, 30% B, 30% C and 40% D. At the final time (5 min) the solvents were 0.0% A, 30% B, 30% C and 40% D. A sufficient washing step in the gradient programme was necessary to remove the accumulated lipophilic matrix solutes. The total analysis time was 5 min. The column thermostat maintained the temperature at 30 °C with the injection volume was 1.0 μl. The autosampler was operated at 20 °C.

The UPLC LG 500 nm system was coupled to a MS/MS detector (Micromass Quattro Ultima PT) via an electrospray interface ﻿(ESI) that operates in negative mode. The optimized ESI settings were as follows: source temperature 120 °C, desolvation temperature 350 °C, drying gas flow 650 L/Hr, cone gas flow 30 L/Hr and capillary voltage 3.50 kV. Nitrogen is used as drying and collision gas (2.67 × 10^−6^ bar). Multiply monitoring reaction (MRM) mode was applied in the MS during the detection and two ion transitions were scanned for each target toxin. The MRM mode was applied in the MS/MS detector and two ion transitions (quantifier and qualifier) were recorded for each target compound. The selected ion transitions with the optimised voltages (cone or tube lens), collision energies (CE) and dwell times are summarised in table (Supplementary Table S2).

### Assessment of toxicological potential of different *Alternaria* mycotoxins (TeA, AOH and AME)

Measurement of extent of cell death as induced by different mycotoxins were determined by detached leaf inoculation method. For this, fresh leaf samples were detached from the green house grown plants and properly washed for 3–4 min in running tap water, sterilized in 1.0% (0.01 g/ml) sodium hypochlorite for about 1 min and then surface wiping of 70% ethanol, and finally aseptically rinsed thoroughly with sterile distilled water. Finally, the leaves were placed on moistened filter paper and punctured by a sterile needle on the lower surface. The toxins were dissolved in sterile deionized water at concentration of 100 μg/ml. Droplets (100 µl) of each of the three toxins were injected to the wounded leaves with a fine needle (Dispovan, 1 ml). A control sample was adjusted by injecting sterile distilled water. The treated leaf samples were maintained inside moist chamber (27 ± 0.5 °C temp. and 60% relative humidity) under green house conditions (14 h light and 10 h dark cycle at 27 °C). A regular observation (every 24 h for 6 days) was made in order to find out any changes relevant to toxicological effects as developed in injected samples with respect to control samples. The experimental set up was maintained in three replicates and the percentage affected leaf area due to toxic induced cell death were measured by (Systronic leaf area meter 211) and calculated by the formula as given below.

$$ \% \,{\rm{diseased}}\,{\rm{leaf}}\,{\rm{area}}={\rm{diseased}}\,{\rm{leaf}}\,{\rm{area}}/{\rm{total}}\,{\rm{leaf}}\,{\rm{area}}\times 100$$

### Determination of cell death by Evans blue uptake assay

The loss of cell viability (cell death) was evaluated using Evans blue staining method (Baker and Mock, 1994). The tomato leaves were treated with the same concentration (250 μg/ml) of all three toxins. Treated leaves were stained with 0.25% (v/v) aqueous solution of Evans blue for 15 min. After washing with distilled water for 30 min, the leaves were excised and soaked with 500 µl of N, N-dimethylformamide for 1 h at room temperature. Optical density of the released Evans blue was measured spectrophotometrically at 600 nm.

### Statistical analysis

Statistical analysis was performed by using IBM SPSS Statistics ver. 20 software via analysis of variance (one-way ANOVA) followed by Duncan’s multiple range test at the *P* ≤ 0.05 significance level. Data were expressed as mean ± standard deviation (SD) of at least three replicates of each metabolite. Statistical data analyses were analyzed in selected 48 isolates of *Alternaria* species which were pathogenic in nature.

## Electronic supplementary material

SUPPLEMENTARY INFO

Supplementary Figure S3
