# Supplementary material for: Isolation, characterization and toxicological potential of Alternaria-mycotoxins (TeA, AOH and AME) in different Alternaria species from various regions of India
Source: Sci Rep. 2017 Aug 18;7:8777. doi: 10.1038/s41598-017-09138-9 (PMC5562829; doi:10.1038/s41598-017-09138-9)
Supplement: Supplementary file 1 — SUPPLEMENTARY INFO [file 41598_2017_9138_MOESM1_ESM.pdf]

Isolation, characterization and toxicological potential of Alternaria-mycotoxins (TeA, AOH and AOH) in different Alternaria species from various regions of India

Mukesh Meena*, Prashant Swapnil and R. S. Upadhyay

Department of Botany, Institute of Science, Banaras Hindu University, Varanasi 221005, India

***Corresponding Author**:
Mukesh Meena,
Department of Botany,
Institute of Science,
Banaras Hindu University,
Varanasi 221 005, India
Email: mukeshmeenabhu@gmail.com

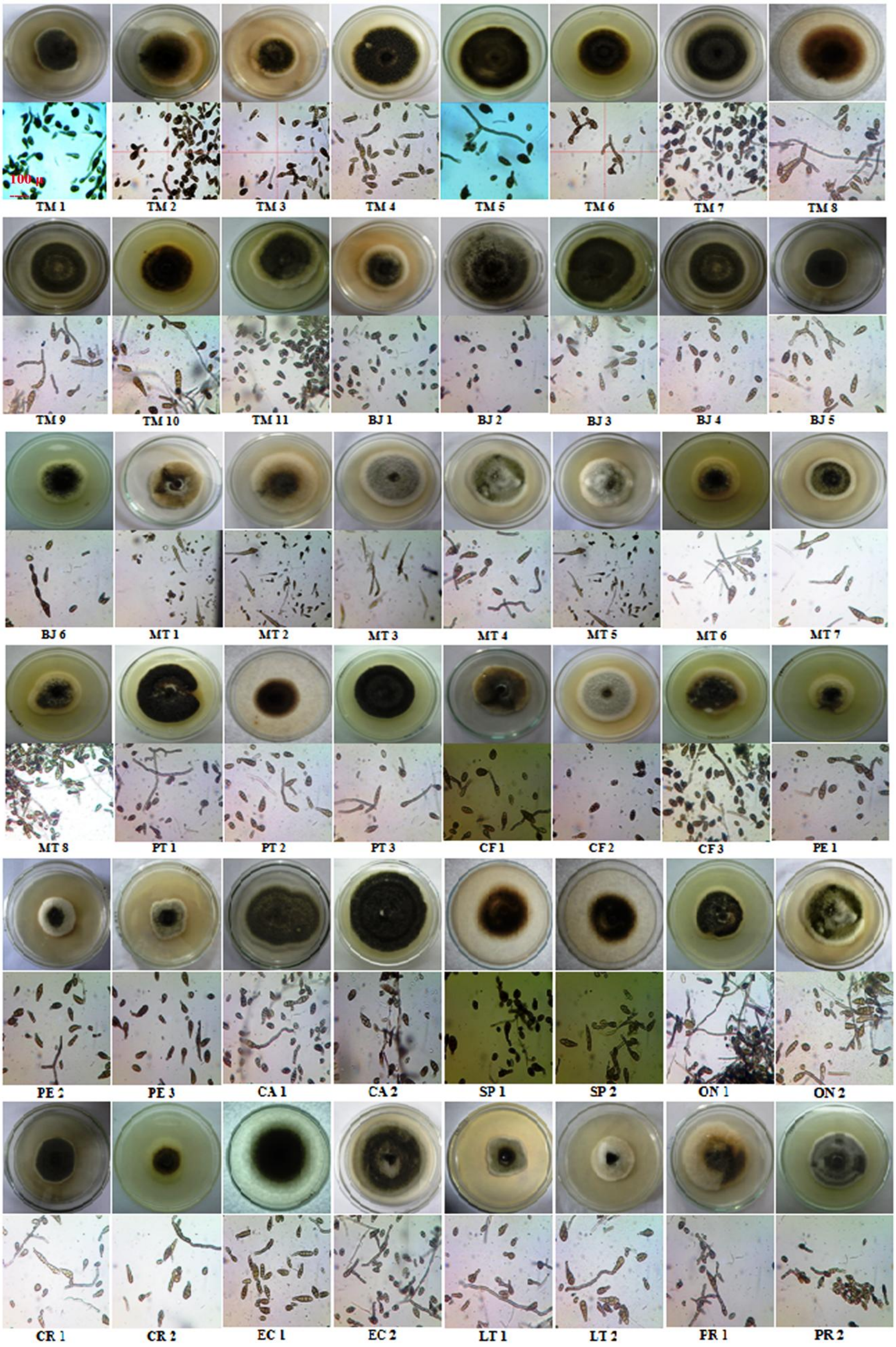

Supplementary Figure S1. Different isolates of *Alternaria* pathogen grown on PDA plate and its conidial morphology isolated from vegetables, crops, and weeds from different regions of India; Scale bar represents 100 µ.

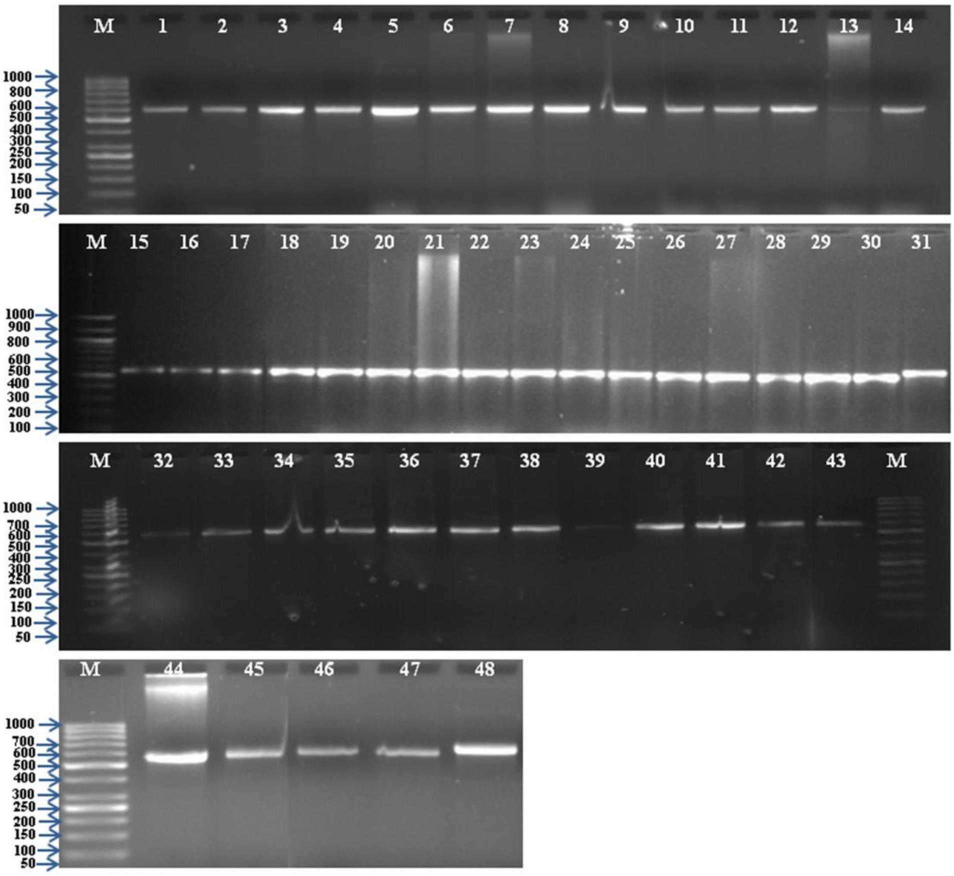

Supplementary Figure S2. Gel electrophoresis of PCR products with primers forward and reverse (ITS1/ITS4) of DNA from fungal isolates. Picture shows the amplified product of all 48 isolates of *Alternaria* species. The amplified products were found between 500-600 bp.

Supplementary Figure S3. Multiple sequence alignment of all the *Alternaria* spp. isolates showing conservation of residues as revealed through CLC Sequence Viewer 6.8.2.

(Supplementary Figure S3 given in separate PDF Image)

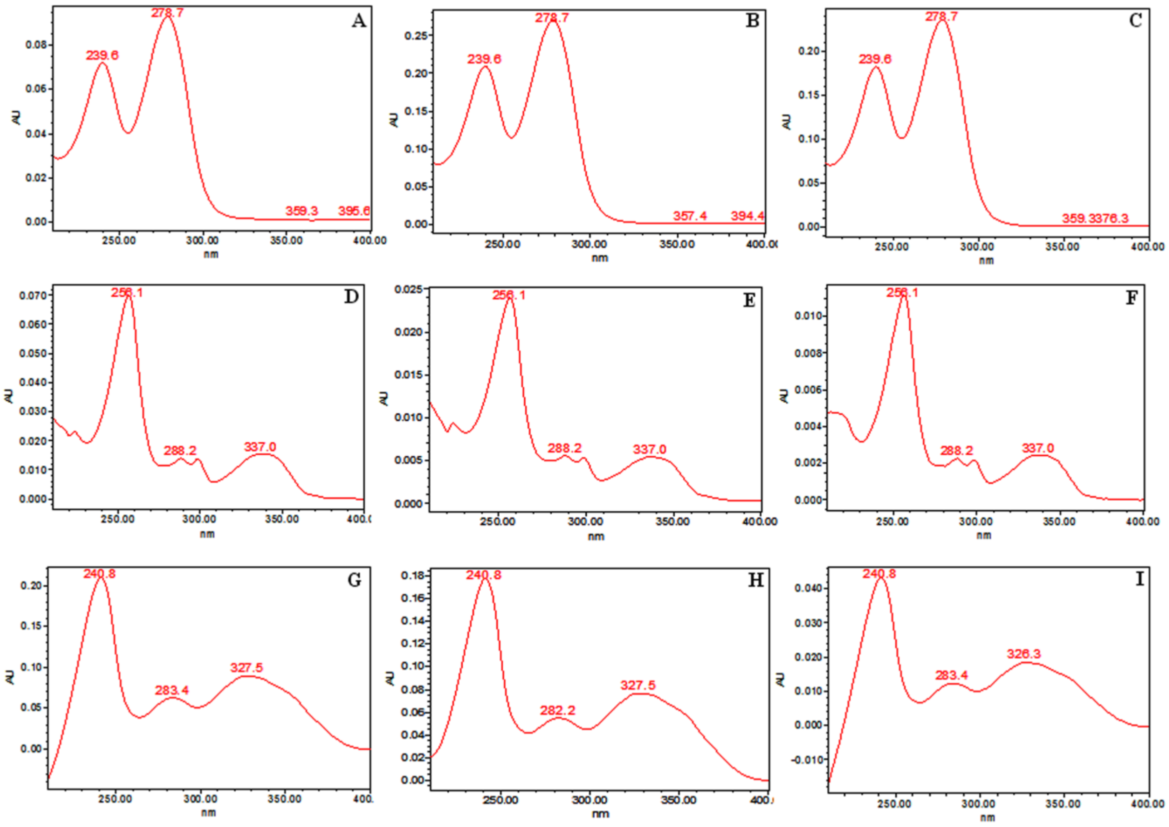

Supplementary Figure S4. Comparison of UV spectra of standard metabolites and isolated fungal metabolites from HPLC connected with diode array detector. **(A)** absorption peaks of standard TeA at 239.6 nm and 278.7 nm **(D)** absorption peaks of standard AOH at 256.1 nm, 288.2 nm and 337.0 nm **(G)** absorption peaks of standard AME at 240.8 nm, 283.4 nm and 327.5 nm **(B)** absorbance peaks of TeA (maximum conc.) as recorded form isolate TM 4 **(C)** absorbance peaks of TeA (minimum conc.) as recorded form isolate PE 1 **(E)** absorbance peaks of AOH (maximum conc.) form isolate TM 4 **(F)** absorbance peaks of AOH (minimum conc.) from isolate ON 1 **(H)** absorbance peaks of AME (maximum conc.) from isolate TM 4 **(I)** absorbance peaks of AME (minimum conc.) from BJ 6

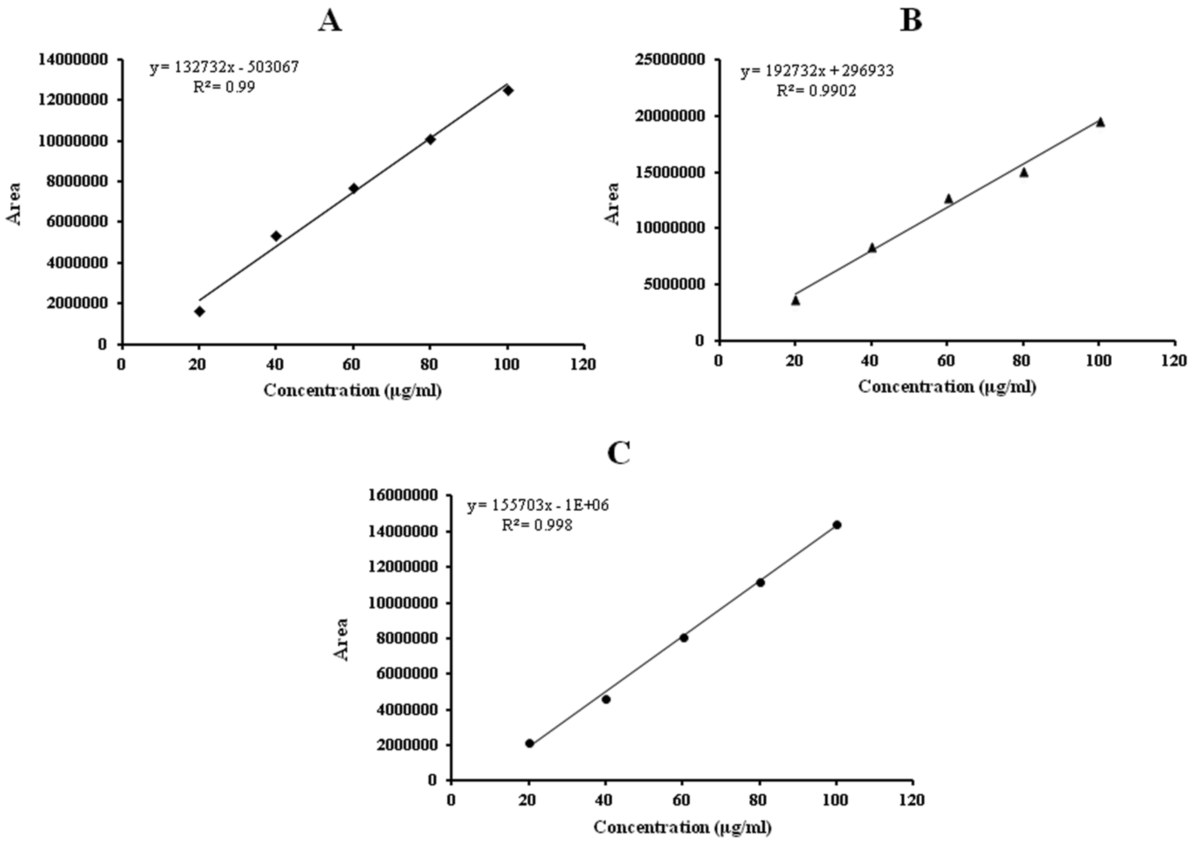

Supplementary Figure S5. Calibration curve of (A) tenuazonic acid, (B) alternariol, and (C) alternariol monomethyl ether standards.

Supplementary Table S1. LOD, LOQ, coefficient r^2 and calibration curve equation of each toxin (TeA, AOH, and AME) of *Alternaria*.

Toxins	LOD (μg/ml)	LOQ (μg/ml)	coefficient r^2	equation
TeA	28.42	86.13	0.99	Y=132732x-503067
AOH	28.03	84.94	0.9902	Y=192732x+296933
AME	12.66	38.36	0.998	Y=155703x-1E+06

Supplementary Table S2. Ion source setting on Accucore RP-MS 100 X 3, 2.6 μm, ACQ-TQD-QBB1152 instrument.

**Settings**	**ES+**		**ES-**	
Source temperature (°C)	120	118	120	118
Desolvation temperature (°C)	350	346	350	346
Cone gas flow (L/Hr)	30	30	30	30
Desolvation gas flow (L/Hr)	650	650	650	650
Capillary voltage (KV)	3.50	3.49	3.50	3.49
Collision gas flow (mL/Min)	0.18	0.14	0.10	0.14
Ion energy 1	1.00		1.00	
Ion energy 2	3.00		1.00	
MS mode entrance	50.00		50.00	
MS mode collision energy	5.00		3.00	
MS mode exit	50.00		50.00	
MS/MS mode entrance	2.00		2.00	
MS/MS mode collision energy	15.00		25.00	
MS/MS mode exit	2.00		2.00	
HM RF Lens (V)	(+) 0.0		(-) 0.0	
LM 1 resolution	12.00		12.00	
HM 1 resolution	12.00		12.00	
LM 2 resolution	15.00		15.00	
HM 2 resolution	15.00		15.00	
Multiplier	-504.50		-504.50	

Note: Ion mode: ESI; Ion polarity: Positive and negative
(MS method: C:\MassLynx\INTERNAL2011.pro\ACQUDB\SAIF9086.EXP)
