# Supplementary material for: Isolation, characterization and toxicological potential of Alternaria-mycotoxins (TeA, AOH and AME) in different Alternaria species from various regions of India
Source: Sci Rep. 2017 Aug 18;7:8777. doi: 10.1038/s41598-017-09138-9 (PMC5562829; doi:10.1038/s41598-017-09138-9)
Supplement: Supplementary file 2 — Supplementary Figure S3 [file 41598_2017_9138_MOESM2_ESM.pdf]

		20		40		
KX118413.1_Alternaria_alternata_strain_BHU-LMMT02	----------	----------	----------	----------	----------	-
KX118415.1_Alternaria_alternata_strain_BHU-LMMT04	----------	----------	----------	----------	----------	-
KX118418.1_Alternaria_alternata_strain_BHU-LMMT07	----------	----------	----------	----------	----------	-
KX118419.1_Alternaria_alternata_strain_BHU-LMMT08	----------	----------	----------	----------	----------	-
KX118421.1_Alternaria_brassicicola_strain_BHU-LMMT10	----------	----------	----------	----------	----------	-
KX118425.1_Alternaria_brassicae_strain_BHU-LMMT14	----------	----------	----------	----------	----------	-
KX118426.1_Alternaria_brassicae_strain_BHU-LMMT15	----------	----------	----------	----------	----------	-
KX118427.1_Alternaria_solani_strain_BHU-LMMT16	----------	----------	----------	----------	----------	-
KX118424.1_Alternaria_brassicicola_strain_BHU-LMMT13	----------	----------	----------	----------	----------	-
KX118414.1_Alternaria_alternata_strain_BHU-LMMT03	----------	----------	----------	----------	----------	-
KX118423.1_Alternaria_brassicicola_strain_BHU-LMMT12	----------	----------	----------	----------	----------	-
KX118412.1_Alternaria_alternata_strain_BHU-LMMT01	----------	----------	----------	----------	----------	-
KX118417.1_Alternaria_alternata_strain_BHU-LMMT06	----------	----------	----------	----------	----------	-
KX118420.1_Alternaria_alternata_strain_BHU-LMMT09	----------	----------	----------	----------	----------	-
KX118422.1_Alternaria_brassicicola_strain_BHU-LMMT11	----------	----------	----------	----------	----------	-
KX179478.1_Alternaria_alternata_strain_BHU-LMMT35	----------	----------	----------	----------	----------	-
KX118416.1_Alternaria_alternata_strain_BHU-LMMT05	----------	----------	----------	----------	----------	-
KX179483.1_Alternaria_alternata_strain_BHU-LMMT40	----------	----------	----------	----------	----------	-
KX179484.1_Alternaria_alternata_strain_BHU-LMMT41	----------	----------	----------	----------	----------	-
KX179485.1_Alternaria_alternata_strain_BHU-LMMT42	----------	----------	----------	----------	----------	-
KX179482.1_Alternaria_solani_strain_BHU-LMMT39	----------	----------	----------	----------	----------	-
KX139158.1_Alternaria_tenuissima_strain_BHU-LMMT25	----------	----------	----------	----------	----------	-
JQ070079.1	----------	----------	----------	----------	-----SEQUE	5
KX179480.1_Alternaria_alternata_strain_BHU-LMMT37	----------	----------	----------	----------	----------	-
KX179479.1_Alternaria_alternata_strain_BHU-LMMT36	----------	----------	----------	----------	----------	-
KX139153.1_Alternaria_brassicae_strain_BHU-LMMT20	----------	----------	----------	-----TTTTC	CGTAGGTGAA	15
KX179489.1_Alternaria_alternata_strain_BHU-LMMT46	----------	----------	----------	------CTTC	CGTAGGGTNA	14
KX139163.1_Alternaria_eichhorniae_strain_BHU-LMMT30	----------	----------	----------	----------	----------	-
KX179488.1_Alternaria_alternata_strain_BHU-LMMT45	----------	----------	----------	----------	----------	-
KX179481.1_Alternaria_solani_strain_BHU-LMMT38	----------	----------	----------	----------	----------	-
KX179487.1_Alternaria_sp._2_MM-2016	----------	----------	----------	----------	-------GGG	3
KX139164.1_Alternaria_eichhorniae_strain_BHU-LMMT31	----------	----------	----------	----------	----------	-
KX179490.1_Alternaria_sp._3_MM-2016	----------	----------	AAAGTGCGAC	AAGGTACTCC	GTAGGGTGAA	30
KX179477.1_Alternaria_alternata_strain_BHU-LMMT34	----------	----------	----------	----------	-----AACGC	5
KX179486.1_Alternaria_sp._1__MM-2016	----------	----------	----------	----------	---------G	1
KX179491.1_Alternaria_sp._4_MM-2016	----------	---GGGAAGT	AAAAGTCGTA	ACAAGGTCTC	CGTAGGTGAA	37
KX139154.1_Alternaria_brassicae_strain_BHU-LMMT21	---------T	AGGTGGAAGT	AAAAGTCGTA	ACAAGGTCTC	CGTAGGTGAA	41
KX139152.1_Alternaria_brassicae_strain_BHU-LMMT19	----------	----------	----------	----------	----------	-
KX139165.1_Alternaria_macrospora_strain_BHU-LMMT32	----------	----------	----------	----------	----------	-
KX139166.1_Alternaria_macrospora_strain_BHU-LMMT33	----------	----------	----------	----------	----------	-
KX139150.1_Alternaria_solani_strain_BHU-LMMT17	----------	----------	----------	----------	----------	-
KX139151.1_Alternaria_solani_strain_BHU-LMMT18	TTGGTTCATT	TAGAGGAAGT	AAAAGTCGTA	ACAAGGTCTC	CGTAGGTGAA	50
KX139162.1_Alternaria_porri_strain_BHU-LMMT29	TTGGT-CATT	TAGAGGAAGT	AAAAGTCGTA	ACAAGGTCTC	CGTAGGTGAA	49
KX139159.1_Alternaria_brassicicola_strain_BHU-LMMT26	----------	----------	----------	----------	----------	-
KX139160.1_Alternaria_brassicicola_strain_BHU-LMMT27	----------	----------	----------	----------	--------AT	2
KX139157.1_Alternaria_tenuissima_strain_BHU-LMMT24	----------	----------	----------	----------	----------	-
KX139161.1_Alternaria_porri_strain_BHU-LMMT28	----------	----------	----------	----CCTCTC	CGTAGGTGAA	16
KX139155.1_Alternaria_brassicae_strain_BHU-LMMT22	----------	----------	----------	----------	----------	-
KX139156.1_Alternaria_brassicae_strain_BHU-LMMT23	----------	----------	----------	----------	----------	-
Consensus	----------	----------	----------	----------	----------	
Conservation (100% – 0%)						

Create PDF files without this message by purchasing novaPDF printer (http://www.novapdf.com)

	60		80		100	
KX118413.1_Alternaria_alternata_strain_BHU-LMMT02	----------	----------	----------	------GGCT	GGA----CCT	10
KX118415.1_Alternaria_alternata_strain_BHU-LMMT04	----------	----------	----------	---GCGGGCT	GGA----CCT	13
KX118418.1_Alternaria_alternata_strain_BHU-LMMT07	----------	----------	----------	-----GGGCT	GGA----CCT	11
KX118419.1_Alternaria_alternata_strain_BHU-LMMT08	----------	----------	----------	-----GGGCT	GGA----CCT	11
KX118421.1_Alternaria_brassicicola_strain_BHU-LMMT10	----------	----------	----------	------GGCT	GGA----CCT	10
KX118425.1_Alternaria_brassicae_strain_BHU-LMMT14	----------	----------	----------	-----GGGCT	GGA----CCT	11
KX118426.1_Alternaria_brassicae_strain_BHU-LMMT15	----------	----------	----------	------GGCT	GGA----CCT	10
KX118427.1_Alternaria_solani_strain_BHU-LMMT16	----------	----------	----------	-----GGGCT	GGA----CCT	11
KX118424.1_Alternaria_brassicicola_strain_BHU-LMMT13	----------	----------	----------	-----GGGCT	GGA----CCT	11
KX118414.1_Alternaria_alternata_strain_BHU-LMMT03	----------	----------	----------	----CGGGCT	GGA----CCT	12
KX118423.1_Alternaria_brassicicola_strain_BHU-LMMT12	----------	----------	----------	----CGGGCT	GGA----CCT	12
KX118412.1_Alternaria_alternata_strain_BHU-LMMT01	----------	----------	----------	------GGCT	GGA----CCT	10
KX118417.1_Alternaria_alternata_strain_BHU-LMMT06	----------	----------	----------	----CGGGCT	GGA----CCT	12
KX118420.1_Alternaria_alternata_strain_BHU-LMMT09	----------	----------	----------	----CGGGCT	GGA----CCT	12
KX118422.1_Alternaria_brassicicola_strain_BHU-LMMT11	----------	----------	----------	----CGGGCT	GGA----CCT	12
KX179478.1_Alternaria_alternata_strain_BHU-LMMT35	----------	----------	----------	---GGGGGCT	GGA----CCT	13
KX118416.1_Alternaria_alternata_strain_BHU-LMMT05	----------	----------	----------	----------	GGA----CCT	6
KX179483.1_Alternaria_alternata_strain_BHU-LMMT40	----------	----------	---C--TCAT	GAG-CGGGCT	GGA----CCT	20
KX179484.1_Alternaria_alternata_strain_BHU-LMMT41	----------	----------	-GGA--CCCT	GAG-CGGGCT	GGA----CCT	22
KX179485.1_Alternaria_alternata_strain_BHU-LMMT42	----------	---------G	GAAA--ATAT	GAGGCGGGCT	GGA----CCT	25
KX179482.1_Alternaria_solani_strain_BHU-LMMT39	----------	----------	---T--TAAT	GAG-CGGGCT	GGA----CCT	20
KX139158.1_Alternaria_tenuissima_strain_BHU-LMMT25	----------	-------AAA	TCAA--ATAT	GAGGCGGGCT	GGA----CCT	27
JQ070079.1	NCEGCGGAGG	GATCATTACA	CAAA--TATG	AAGGCGGGCT	GGA---ACCT	50
KX179480.1_Alternaria_alternata_strain_BHU-LMMT37	---GCGGAGG	GATCATTACA	CAAA--TATG	AAGGCGGGCT	GGA---ACCT	42
KX179479.1_Alternaria_alternata_strain_BHU-LMMT36	---GCGGAGG	GATCATTACA	CAAA--TATG	AAGGCGGGCT	GGA---ACCT	42
KX139153.1_Alternaria_brassicae_strain_BHU-LMMT20	CCTGCGGAGG	GATCATTACA	CAAA--TATG	AAGGCGGGCT	GGA---ACCT	60
KX179489.1_Alternaria_alternata_strain_BHU-LMMT46	CCTGCGGAGG	GATCATTACA	CAAA--TATG	AAGGCGGGCT	GGA---ACCT	59
KX139163.1_Alternaria_eichhorniae_strain_BHU-LMMT30	----------	-ATCATTACA	CAAA--TATG	AAGGCGGGCT	GGA---ATCT	34
KX179488.1_Alternaria_alternata_strain_BHU-LMMT45	----------	CCTCATTACA	CAAA--TATG	AAGGCGGGCT	GGA---ACCT	35
KX179481.1_Alternaria_solani_strain_BHU-LMMT38	--------GG	GTTTTGTGTC	ATGG--TATG	A-GGCGGGCT	GGA----CCT	35
KX179487.1_Alternaria_sp._2_MM-2016	CCCAAGTGCG	ACTC-TTACA	CAA---TATG	A-GGCGGGCT	GGA---ACCT	45
KX139164.1_Alternaria_eichhorniae_strain_BHU-LMMT31	----------	AATCATTACA	CAAA--TATG	AAGGCGGGCT	GGA---ACCT	35
KX179490.1_Alternaria_sp._3_MM-2016	CCTGCGGGGG	GATCATTACA	CAAA--TATG	AAGGCGGGCT	GGA---ACCT	75
KX179477.1_Alternaria_alternata_strain_BHU-LMMT34	CATTGCC-TG	CAAAGTCTTA	CCAA--TATG	-AGGCGGGCT	GGA----CCT	47
KX179486.1_Alternaria_sp._1__MM-2016	GTTTGCAATG	CGA-GTCTTA	CCAA--TATG	-AGGCGGGCT	GGA---ACCT	44
KX179491.1_Alternaria_sp._4_MM-2016	CCTGCGGAGG	GATCATTACA	CAAA--TATG	AAGGCGGGCT	GGA---ACCT	82
KX139154.1_Alternaria_brassicae_strain_BHU-LMMT21	CCTGCGGAGG	GATCATTACA	C-AA--TATG	AAGGCAGGCT	GAA---ATCT	85
KX139152.1_Alternaria_brassicae_strain_BHU-LMMT19	----------	----------	----------	----------	-AA---ATCT	6
KX139165.1_Alternaria_macrospora_strain_BHU-LMMT32	-------AGG	GATCATTACA	CAAA--TATG	AAGGCGGGCT	GGC---ACCT	38
KX139166.1_Alternaria_macrospora_strain_BHU-LMMT33	----------	-ATCATTACA	CAAA--TATG	AAGGCGGGCT	GGC---ACCT	34
KX139150.1_Alternaria_solani_strain_BHU-LMMT17	----------	----------	----------	----CGGGCT	GGC---ACCT	13
KX139151.1_Alternaria_solani_strain_BHU-LMMT18	CCTGCGGAGG	GATCATTACA	CAAAAATATG	AAGGCGGGCT	GGC---ACCT	97
KX139162.1_Alternaria_porri_strain_BHU-LMMT29	CCTGCGGAGG	GATCATTACA	CAAA--TATG	AAGGCGGGCT	GGC---ACCT	94
KX139159.1_Alternaria_brassicicola_strain_BHU-LMMT26	----------	-ATCATTACA	CAA---TATG	AAAGCGGGCT	GGACTCACCT	36
KX139160.1_Alternaria_brassicicola_strain_BHU-LMMT27	C-------GG	GATCATTACA	CAA---TATG	AAAGCGGGCT	GGACTCACCT	42
KX139157.1_Alternaria_tenuissima_strain_BHU-LMMT24	---TTGCGAG	GATCTT----	--AAAGTATG	-AGGCGGGCT	GGA----CCT	36
KX139161.1_Alternaria_porri_strain_BHU-LMMT28	CCTGCGGAAG	GATCATTACC	TAGAGTTGTA	------GGCT	----TTGCCT	56
KX139155.1_Alternaria_brassicae_strain_BHU-LMMT22	----------	----------	----------	----------	----------	-
KX139156.1_Alternaria_brassicae_strain_BHU-LMMT23	----------	----------	----------	----------	----------	-
Consensus	----------	----------	--A---TATG	-AGGCGGGCT	GGA----CCT	

Conservation

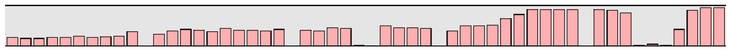

Create PDF files without this message by purchasing novaPDF printer (http://www.novapdf.com)

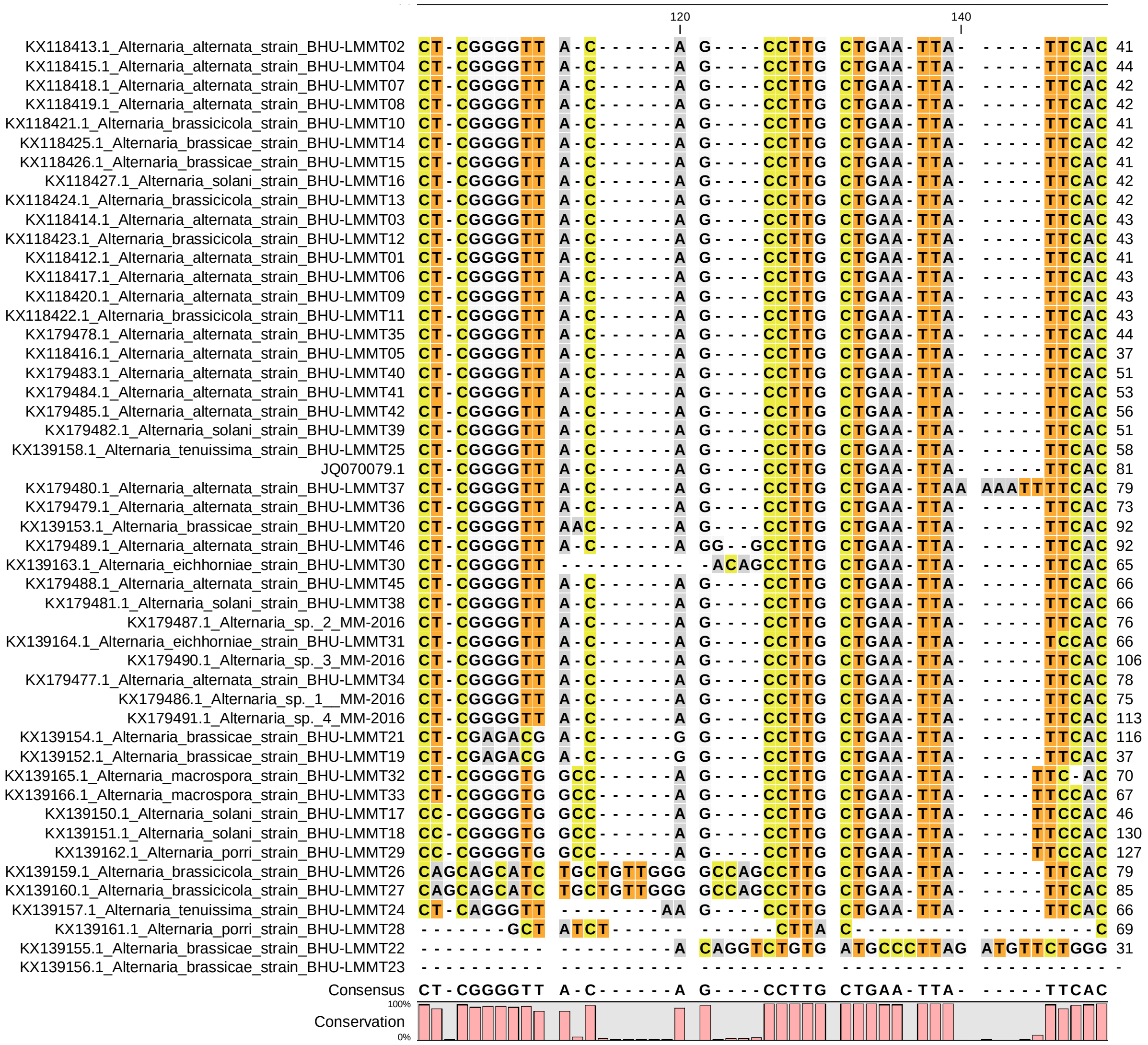

Create PDF files without this message by purchasing novaPDF printer (http://www.novapdf.com)

	160		180		200	
KX118413.1_Alternaria_alternata_strain_BHU-LMMT02	CCTTG--TCT	TTTG--CGTA	C------TTC	TTGTT--T-C	CTTGGTGGGT	78
KX118415.1_Alternaria_alternata_strain_BHU-LMMT04	CCTTG--TCT	TTTG--CGTA	C------TTC	TTGTT--T-C	CTTGGTGGGT	81
KX118418.1_Alternaria_alternata_strain_BHU-LMMT07	CCTTG--TCT	TTTG--CGTA	C------TTC	TTGTT--T-C	CTTGGTGGGT	79
KX118419.1_Alternaria_alternata_strain_BHU-LMMT08	CCTTG--TCT	TTTG--CGTA	C------TTC	TTGTT--T-C	CTTGGTGGGT	79
KX118421.1_Alternaria_brassicicola_strain_BHU-LMMT10	CCTTG--TCT	TTTG--CGTA	C------TTC	TTGTT--T-C	CTTGGTGGGT	78
KX118425.1_Alternaria_brassicae_strain_BHU-LMMT14	CCTTG--TCT	TTTG--CGTA	C------TTC	TTGTT--T-C	CTTGGTGGGT	79
KX118426.1_Alternaria_brassicae_strain_BHU-LMMT15	CCTTG--TCT	TTTG--CGTA	C------TTC	TTGTT--T-C	CTTGGTGGGT	78
KX118427.1_Alternaria_solani_strain_BHU-LMMT16	CCTTG--TCT	TTTG--CGTA	C------TTC	TTGTT--T-C	CTTGGTGGGT	79
KX118424.1_Alternaria_brassicicola_strain_BHU-LMMT13	CCTTG--TCT	TTTG--CGTA	C------TTC	TTGTT--T-C	CTTGGTGGGT	79
KX118414.1_Alternaria_alternata_strain_BHU-LMMT03	CCTTG--TCT	TTTG--CGTA	C------TTC	TTGTT--T-C	CTTGGTGGGT	80
KX118423.1_Alternaria_brassicicola_strain_BHU-LMMT12	CCTTG--TCT	TTTG--CGTA	C------TTC	TTGTT--T-C	CTTGGTGGGT	80
KX118412.1_Alternaria_alternata_strain_BHU-LMMT01	CCTTG--TCT	TTTG--CGTA	C------TTC	TTGTT--T-C	CTTGGTGGGT	78
KX118417.1_Alternaria_alternata_strain_BHU-LMMT06	CCTTG--TCT	TTTG--CGTA	C------TTC	TTGTT--T-C	CTTGGTGGGT	80
KX118420.1_Alternaria_alternata_strain_BHU-LMMT09	CCTTG--TCT	TTTG--CGTA	C------TTC	TTGTT--T-C	CTTGGTGGGT	80
KX118422.1_Alternaria_brassicicola_strain_BHU-LMMT11	CCTTG--TCT	TTTG--CGTA	CCCGGATTTC	TTGTT--T-C	CTTGGTGGGT	86
KX179478.1_Alternaria_alternata_strain_BHU-LMMT35	CCTTG--TCT	TTTG--CGTA	C------TTC	TTGTT--T-C	CTTGGTGGGT	81
KX118416.1_Alternaria_alternata_strain_BHU-LMMT05	CCTTG--TCT	TTTG--CGTA	C------TTC	TTGTT--T-C	CTTGGTGGGT	74
KX179483.1_Alternaria_alternata_strain_BHU-LMMT40	CCTTG--TCT	TTTGAACGTA	C------TTC	TTGTT--T-C	CTTGGTGGGT	90
KX179484.1_Alternaria_alternata_strain_BHU-LMMT41	CCTTG--TCT	TTTG--CGTA	C------TTC	TTGTT--T-C	CTTGGTGGGT	90
KX179485.1_Alternaria_alternata_strain_BHU-LMMT42	CCTTG--TCT	TTTG--CGTA	C------TTC	TTGTT--T-C	CTTGGTGGGT	93
KX179482.1_Alternaria_solani_strain_BHU-LMMT39	CCTTG--TCT	TTTG--CGTA	C------TTC	TTGTT--T-C	CTTGGTGGGT	88
KX139158.1_Alternaria_tenuissima_strain_BHU-LMMT25	CCTTG--TCT	TTTG--CGTA	C------TTC	TTGTT--T-C	CTTGGTGGGT	95
JQ070079.1	CCTTG--TCT	TTTG--CGTA	C------TTC	TTGTT--T-C	CTTGGTGGGT	118
KX179480.1_Alternaria_alternata_strain_BHU-LMMT37	CCTTG--TCT	TTTG--CGTA	C------TTC	TTGTT--T-C	CTTGGTGGGT	116
KX179479.1_Alternaria_alternata_strain_BHU-LMMT36	CCTTG--TCT	TTTG--CGTA	C------TTC	TTGTT--T-C	CTTGGTGGGT	110
KX139153.1_Alternaria_brassicae_strain_BHU-LMMT20	CCTTG--TCT	TTTG--CGTA	C------TTC	TTGTT--T-C	CTTGGTGGGT	129
KX179489.1_Alternaria_alternata_strain_BHU-LMMT46	CCTTG--TCT	TTTG--CGTA	C------TTC	TTGTT--T-C	CTTGGTGGGT	129
KX139163.1_Alternaria_eichhorniae_strain_BHU-LMMT30	CCTTGGGTCT	TTTG--CGTA	C------TTC	T-GTT--T-C	CTTGGTGGGT	103
KX179488.1_Alternaria_alternata_strain_BHU-LMMT45	CCTTG--TCT	TTTG--CGTA	C------TTC	TTGTT--T-C	CTTGGTGGGT	103
KX179481.1_Alternaria_solani_strain_BHU-LMMT38	CCTTG--TCT	TTTG--CGTA	C------TTC	TTGTT--T-C	CTTGGTGGGT	103
KX179487.1_Alternaria_sp._2_MM-2016	CCTTG--TCT	TTTG--CGTA	C------TTC	TTGTT--T-C	CTTGGTGGGT	113
KX139164.1_Alternaria_eichhorniae_strain_BHU-LMMT31	CCTTGT-TCT	TTTG--CGTA	C------TTC	TTGTT--T-C	CTTGGTGGGT	104
KX179490.1_Alternaria_sp._3_MM-2016	CCTTG--TCT	TTTG--CGTA	C------TTC	TTGTT--T-C	CTTGGTGGGT	143
KX179477.1_Alternaria_alternata_strain_BHU-LMMT34	CCTTG--TCT	TTTG--CGTA	C------TTC	TTGTT--T-C	CTTGGTGGGT	115
KX179486.1_Alternaria_sp._1__MM-2016	CCTTG--TCT	TTTG--CGTA	C------TTC	TTGTT--T-C	CTTGGTGGGT	112
KX179491.1_Alternaria_sp._4_MM-2016	CCTTG--TCT	TTTG--CGTA	C------TTC	TTGTT--T-C	CTTGGTGGGT	150
KX139154.1_Alternaria_brassicae_strain_BHU-LMMT21	CCGTG--TCT	TTTG--CGTA	C------TTC	TTGTT--T-C	CTTGGTGGGC	153
KX139152.1_Alternaria_brassicae_strain_BHU-LMMT19	CCGTG--TCT	TTTG--CGTA	C------TTC	TTGTT--T-C	CTTGGTGGGC	74
KX139165.1_Alternaria_macrospora_strain_BHU-LMMT32	CCG--TGTCT	TTTG--CGTA	C------TTC	TTGTT--T-C	CTTGGTGGGC	107
KX139166.1_Alternaria_macrospora_strain_BHU-LMMT33	CCGGGTGTCT	TTTG--CGTA	C------TTC	TTGTT--T-C	CTTGGTGGGC	106
KX139150.1_Alternaria_solani_strain_BHU-LMMT17	CCG--TGTCT	TTTG--CGTA	C------TTC	TTGTT--TTC	CTTGGTGGGC	84
KX139151.1_Alternaria_solani_strain_BHU-LMMT18	CCGTG--TCT	TTTG--CGTA	C------TTC	TTGTT--T-C	CTTGGTGGGC	167
KX139162.1_Alternaria_porri_strain_BHU-LMMT29	CCGTG--TCT	TTTG--GG--	C------T-C	TTGTT--T-C	CTTGGTGGGC	161
KX139159.1_Alternaria_brassicicola_strain_BHU-LMMT26	CCGTG--TCT	TTTG--CGTA	C------TTC	TTGTT--T-C	CTTGGTGGGC	116
KX139160.1_Alternaria_brassicicola_strain_BHU-LMMT27	CCGTG--TCT	TTTG--CGTA	C------TTC	TTGTT--T-C	CTTGGTGGGC	122
KX139157.1_Alternaria_tenuissima_strain_BHU-LMMT24	CCTTG--TCT	TTTG--CGTA	C------TTC	TTGTT--T-C	CTTGGTGGGT	103
KX139161.1_Alternaria_porri_strain_BHU-LMMT28	CCATG--TCT	TTTG--AGTA	C--------C	TTCGT--TTC	CTCGGCGGGT	105
KX139155.1_Alternaria_brassicae_strain_BHU-LMMT22	CCGCACGCGC	GCTACACTGA	CAGAGCCAAC	GAGTTCTTTC	TTTTCAACGA	81
KX139156.1_Alternaria_brassicae_strain_BHU-LMMT23	----------	----------	----------	----------	----------	-
Consensus	CCTTG--TCT	TTTG--CGTA	C------TTC	TTGTT--T-C	CTTGGTGGGT	

Conservation

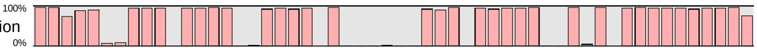

Create PDF files without this message by purchasing novaPDF printer (http://www.novapdf.com)

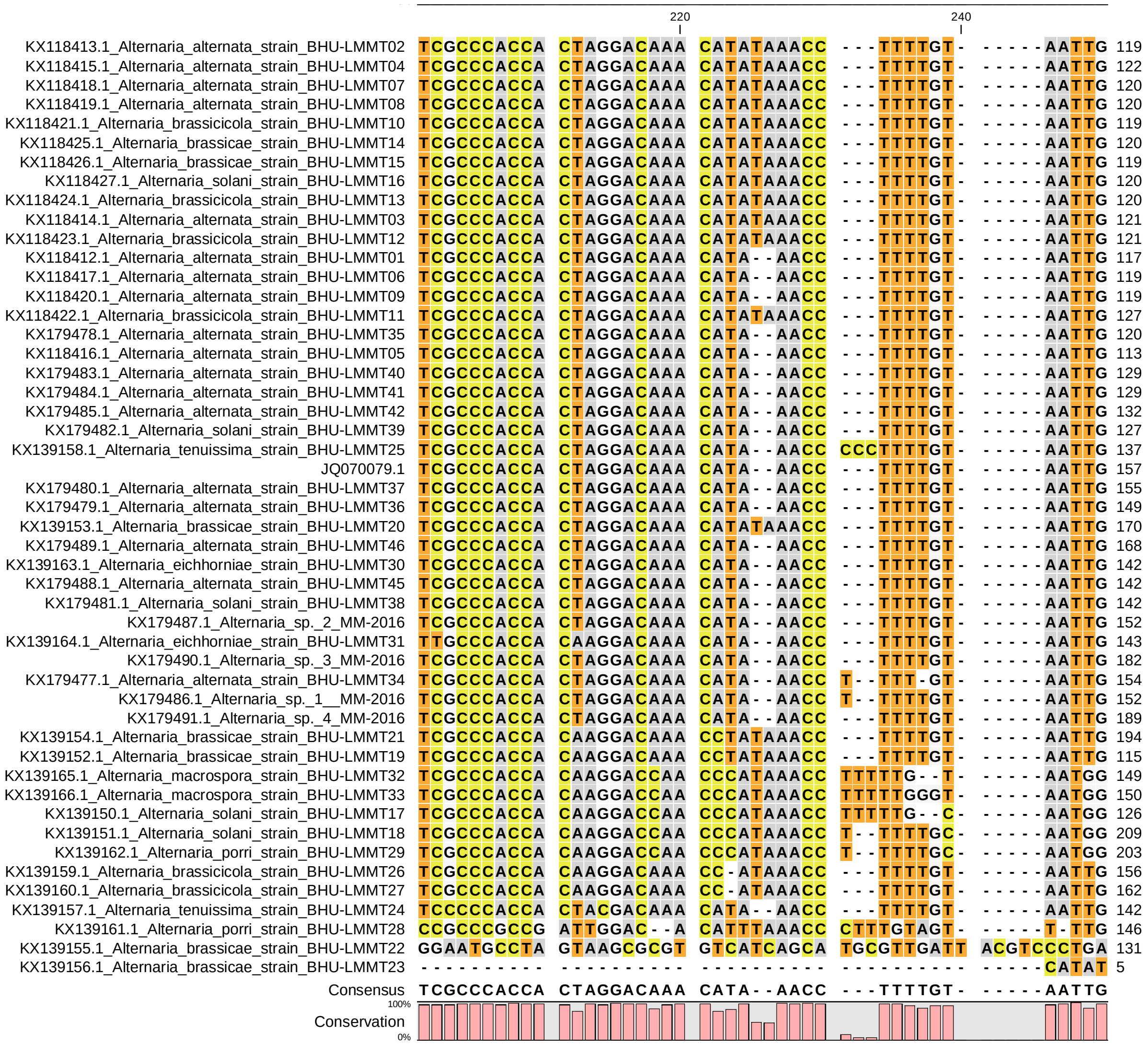

Create PDF files without this message by purchasing novaPDF printer (http://www.novapdf.com)

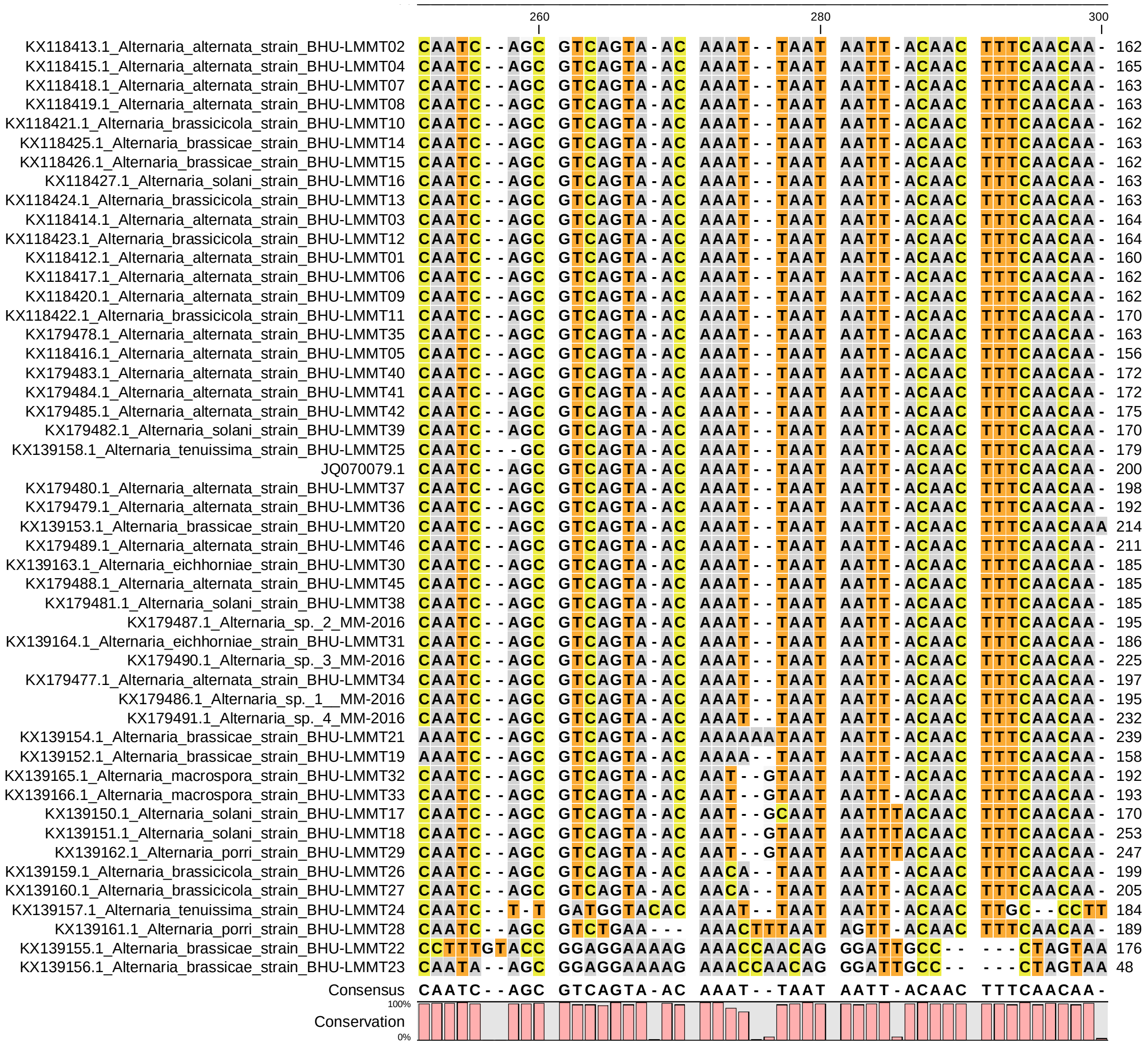

Create PDF files without this message by purchasing novaPDF printer (http://www.novapdf.com)

	320 340	
KX118413.1_Alternaria_alternata_strain_BHU-LMMT02	CGGA---TCT CTTGGTT-CT GGCATCGA-- -TGA--A-GA ACGCAGCGAA	202
KX118415.1_Alternaria_alternata_strain_BHU-LMMT04	CGGA---TCT CTTGGTT-CT GGCATCGA-- -TGA--A-GA ACGCAGCGAA	205
KX118418.1_Alternaria_alternata_strain_BHU-LMMT07	CGGA---TCT CTTGGTT-CT GGCATCGA-- -TGA--A-GA ACGCAGCGAA	203
KX118419.1_Alternaria_alternata_strain_BHU-LMMT08	CGGA---TCT CTTGGTT-CT GGCATCGA-- -TGA--A-GA ACGCAGCGAA	203
KX118421.1_Alternaria_brassicicola_strain_BHU-LMMT10	CGGA---TCT CTTGGTT-CT GGCATCGA-- -TGA--A-GA ACGCAGCGAA	202
KX118425.1_Alternaria_brassicae_strain_BHU-LMMT14	CGGA---TCT CTTGGTT-CT GGCATCGA-- -TGA--A-GA ACGCAGCGAA	203
KX118426.1_Alternaria_brassicae_strain_BHU-LMMT15	CGGA---TCT CTTGGTT-CT GGCATCGA-- -TGA--A-GA ACGCAGCGAA	202
KX118427.1_Alternaria_solani_strain_BHU-LMMT16	CGGA---TCT CTTGGTT-CT GGCATCGA-- -TGA--A-GA ACGCAGCGAA	203
KX118424.1_Alternaria_brassicicola_strain_BHU-LMMT13	CGGA---TCT CTTGGTT-CT GGCATCGA-- -TGA--A-GA ACGCAGCGAA	203
KX118414.1_Alternaria_alternata_strain_BHU-LMMT03	CGGA---TCT CTTGGTT-CT GGCATCGA-- -TGA--A-GA ACGCAGCGAA	204
KX118423.1_Alternaria_brassicicola_strain_BHU-LMMT12	CGGA---TCT CTTGGTT-CT GGCATCGA-- -TGA--A-GA ACGCAGCGAA	204
KX118412.1_Alternaria_alternata_strain_BHU-LMMT01	CGGA---TCT CTTGGTT-CT GGCATCGA-- -TGA--A-GA ACGCAGCGAA	200
KX118417.1_Alternaria_alternata_strain_BHU-LMMT06	CGGA---TCT CTTGGTT-CT GGCATCGA-- -TGA--A-GA ACGCAGCGAA	202
KX118420.1_Alternaria_alternata_strain_BHU-LMMT09	CGGA---TCT CTTGGTT-CT GGCATCGA-- -TGA--A-GA ACGCAGCGAA	202
KX118422.1_Alternaria_brassicicola_strain_BHU-LMMT11	CGGA---TCT CTTGGTT-CT GGCATCGA-- -TGA--A-GA ACGCAGCGAA	210
KX179478.1_Alternaria_alternata_strain_BHU-LMMT35	CGGA---TCT CTTGGTT-CT GGCATCGA-- -TGA--A-GA ACGCAGCGAA	203
KX118416.1_Alternaria_alternata_strain_BHU-LMMT05	CGGA---TCT CTTGGTT-CT GGCATCGA-- -TGA--A-GA ACGCAGCGAA	196
KX179483.1_Alternaria_alternata_strain_BHU-LMMT40	CGGA---TCT CTTGGTT-CT GGCATCGA-- -TGA--A-GA ACGCAGCGAA	212
KX179484.1_Alternaria_alternata_strain_BHU-LMMT41	CGGA---TCT CTTGGTT-CT GGCATCGA-- -TGA--A-GA ACGCAGCGAA	212
KX179485.1_Alternaria_alternata_strain_BHU-LMMT42	CGGA---TCT CTTGGTT-CT GGCATCGA-- -TGA--A-GA ACGCAGCGAA	215
KX179482.1_Alternaria_solani_strain_BHU-LMMT39	CGGA---TCT CTTGGTT-CT GGCATCGA-- -TGA--A-GA ACGCAGCGAA	210
KX139158.1_Alternaria_tenuissima_strain_BHU-LMMT25	CGGA---TCT CTTGGTT-CT GGCATCGA-- -TGA--A-GA ACGCAGCGAA	219
JQ070079.1	CGGA---TCT CTTGGTT-CT GGCATCGA-- -TGA--A-GA ACGCAGCGAA	240
KX179480.1_Alternaria_alternata_strain_BHU-LMMT37	CGGA---TCT CTTGGTT-CT GGCATCGA-- -TGA--A-GA ACGCAGCGAA	238
KX179479.1_Alternaria_alternata_strain_BHU-LMMT36	CGGA---TCT CTTGGTT-CT GGCATCGA-- -TGA--A-GA ACGCAGCGAA	232
KX139153.1_Alternaria_brassicae_strain_BHU-LMMT20	CGGA---TCT CTTGGTT-CT GGCATCGA-- -TGA--A-GA ACGCAGCGAA	254
KX179489.1_Alternaria_alternata_strain_BHU-LMMT46	CGGA---TCT CTTGGTT-CT GGCATCGA-- -TGA--A-GA ACGCAGCGAA	251
KX139163.1_Alternaria_eichhorniae_strain_BHU-LMMT30	CGGA---TCT CTTGGTT-CT GGCATCGA-- -TGACCA-GA ACGCAGCGAA	227
KX179488.1_Alternaria_alternata_strain_BHU-LMMT45	CGGA---TCT CTTGGTT-CT GGCATCGA-- -TGA--A-GA ACGCAGCGAA	225
KX179481.1_Alternaria_solani_strain_BHU-LMMT38	CGGA---TCT CTTGGTT-CT GGCATCGA-- -TGA--A-GA ACGCAGCGAA	225
KX179487.1_Alternaria_sp._2_MM-2016	CGGA---TCT CTTGGTT-CT GGCATCGA-- -TGA--A-GA ACGCAGCGAA	235
KX139164.1_Alternaria_eichhorniae_strain_BHU-LMMT31	CGGA---TCT CTTGGTT-CT GGCATCGA-- -TGA--A-GA ACGCAGCGAA	226
KX179490.1_Alternaria_sp._3_MM-2016	CGGA---TCT CTTGGTT-CT GGCATCGA-- -TGA--A-GA ACGCAGCGAA	265
KX179477.1_Alternaria_alternata_strain_BHU-LMMT34	CGGA---TCT CTTGGTTTCT GGCATCGA-- -TGA--A-GA ACGCAGCGAA	238
KX179486.1_Alternaria_sp._1__MM-2016	CGGA---TCT CTTGGTT-CT GGCATCGA-- -TGA--A-GA ACGCAGCGAA	235
KX179491.1_Alternaria_sp._4_MM-2016	CGGA---TCT CTTGGTT-CT GGCATCGA-- -TGA--A-GA ACGCAGCGAA	272
KX139154.1_Alternaria_brassicae_strain_BHU-LMMT21	CGGA---TCT CTTGGTT-CT GGCATCGA-- -TGA--A-GA ACGCAGCGAA	279
KX139152.1_Alternaria_brassicae_strain_BHU-LMMT19	CGGA---TCT CTTGGTT-CT GGCATCGA-- -TGA--A-GA ACGCAGCGAA	198
KX139165.1_Alternaria_macrospora_strain_BHU-LMMT32	CGGA---TCT CTTGGTT-CT GGCATCGA-- -TGA--A-GA ACGCAGCGAA	232
KX139166.1_Alternaria_macrospora_strain_BHU-LMMT33	CGGA---TCT CTTGGTT-CT GGCATCGA-- -TGA--A-GA ACGCAGCGAA	233
KX139150.1_Alternaria_solani_strain_BHU-LMMT17	CGGA---TCT CTTGGTT-CT GGCATCGA-- -TGA--A-GA ACGCAGCGAA	210
KX139151.1_Alternaria_solani_strain_BHU-LMMT18	CGGA---TCT CTTGGTT-CT GGCATCGA-- -TGAAGA-AA ACGCAGCGAA	295
KX139162.1_Alternaria_porri_strain_BHU-LMMT29	CGGA---TCT CTTGGTT-CT GGCATCGA-- -TGAAGA-A- -CGCAGCGAA	287
KX139159.1_Alternaria_brassicicola_strain_BHU-LMMT26	CGGA---TCT CTTGGTT-CT GGCATCGA-- -TGA--A-GA ACGCAGCGAA	239
KX139160.1_Alternaria_brassicicola_strain_BHU-LMMT27	CGGA---TCT CTTGGTT-CT GGCATCGA-- -TGA--A-GA ACGCAGCGAA	245
KX139157.1_Alternaria_tenuissima_strain_BHU-LMMT24	TGGA---TCT CTTGGGT-CT GGCTGCGA-- -TTA--A-AA ACACACCAAA	224
KX139161.1_Alternaria_porri_strain_BHU-LMMT28	CGGA---TCT CTTGGTT-CT GGCATCGA-- -TGA--A-GA ACGCAGCGAA	229
KX139155.1_Alternaria_brassicae_strain_BHU-LMMT22	CGGCGAGTGA AGCGGCAACA GC--TCAAAT TTGAAATCTG GCTCTTTTAG	224
KX139156.1_Alternaria_brassicae_strain_BHU-LMMT23	CGGCGAGTGA AGCGGCAACA GCCCTCAAAT TTGAAATCTG GCTCTTTTAG	98
Consensus	CGGA---TCT CTTGGTT-CT GGCATCGA-- -TGA--A-GA ACGCAGCGAA	

Conservation

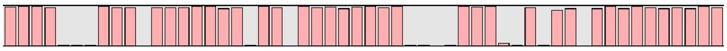

Create PDF files without this message by purchasing novaPDF printer (http://www.novapdf.com)

Sequence	360		380		400	
KX118413.1_Alternaria_alternata_strain_BHU-LMMT02	A--TGCGATA	AGTAGTGTGA	ATTGCAGAAT	TCAGTGAATC	ATCGAA-TCT	249
KX118415.1_Alternaria_alternata_strain_BHU-LMMT04	A--TGCGATA	AGTAGTGTGA	ATTGCAGAAT	TCAGTGAATC	ATCGAA-TCT	252
KX118418.1_Alternaria_alternata_strain_BHU-LMMT07	A--TGCGATA	AGTAGTGTGA	ATTGCAGAAT	TCAGTGAATC	ATCGAA-TCT	250
KX118419.1_Alternaria_alternata_strain_BHU-LMMT08	A--TGCGATA	AGTAGTGTGA	ATTGCAGAAT	TCAGTGAATC	ATCGAA-TCT	250
KX118421.1_Alternaria_brassicicola_strain_BHU-LMMT10	A--TGCGATA	AGTAGTGTGA	ATTGCAGAAT	TCAGTGAATC	ATCGAA-TCT	249
KX118425.1_Alternaria_brassicae_strain_BHU-LMMT14	A--TGCGATA	AGTAGTGTGA	ATTGCAGAAT	TCAGTGAATC	ATCGAA-TCT	250
KX118426.1_Alternaria_brassicae_strain_BHU-LMMT15	A--TGCGATA	AGTAGTGTGA	ATTGCAGAAT	TCAGTGAATC	ATCGAA-TCT	249
KX118427.1_Alternaria_solani_strain_BHU-LMMT16	A--TGCGATA	AGTAGTGTGA	ATTGCAGAAT	TCAGTGAATC	ATCGAA-TCT	250
KX118424.1_Alternaria_brassicicola_strain_BHU-LMMT13	A--TGCGATA	AGTAGTGTGA	ATTGCAGAAT	TCAGTGAATC	ATCGAA-TCT	250
KX118414.1_Alternaria_alternata_strain_BHU-LMMT03	A--TGCGATA	AGTAGTGTGA	ATTGCAGAAT	TCAGTGAATC	ATCGAA-TCT	251
KX118423.1_Alternaria_brassicicola_strain_BHU-LMMT12	A--TGCGATA	AGTAGTGTGA	ATTGCAGAAT	TCAGTGAATC	ATCGAA-TCT	251
KX118412.1_Alternaria_alternata_strain_BHU-LMMT01	A--TGCGATA	AGTAGTGTGA	ATTGCAGAAT	TCAGTGAATC	ATCGAA-TCT	247
KX118417.1_Alternaria_alternata_strain_BHU-LMMT06	A--TGCGATA	AGTAGTGTGA	ATTGCAGAAT	TCAGTGAATC	ATCGAA-TCT	249
KX118420.1_Alternaria_alternata_strain_BHU-LMMT09	A--TGCGATA	AGTAGTGTGA	ATTGCAGAAT	TCAGTGAATC	ATCGAA-TCT	249
KX118422.1_Alternaria_brassicicola_strain_BHU-LMMT11	A--TGCGATA	AGTAGTGTGA	ATTGCAGAAT	TCAGTGAATC	ATCGAA-TCT	257
KX179478.1_Alternaria_alternata_strain_BHU-LMMT35	A--TGCGATA	AGTAGTGTGA	ATTGCAGAAT	TCAGTGAATC	ATCGAA-TCT	250
KX118416.1_Alternaria_alternata_strain_BHU-LMMT05	A--TGCGATA	AGTAGTGTGA	ATTGCAGAAT	TCAGTGAATC	ATCGAA-TCT	243
KX179483.1_Alternaria_alternata_strain_BHU-LMMT40	A--TGCGATA	AGTAGTGTGA	ATTGCAGAAT	TCAGTGAATC	ATCGAA-TCT	259
KX179484.1_Alternaria_alternata_strain_BHU-LMMT41	A--TGCGATA	AGTAGTGTGA	ATTGCAGAAT	TCAGTGAATC	ATCGAA-TCT	259
KX179485.1_Alternaria_alternata_strain_BHU-LMMT42	A--TGCGATA	AGTAGTGTGA	ATTGCAGAAT	TCAGTGAATC	ATCGAA-TCT	262
KX179482.1_Alternaria_solani_strain_BHU-LMMT39	A--TGCGATA	AGTAGTGTGA	ATTGCAGAAT	TCAGTGAATC	ATCGAA-TCT	257
KX139158.1_Alternaria_tenuissima_strain_BHU-LMMT25	A--TGCGATA	AGTAGTGTGA	ATTGCAGAAT	TCAGTGAATC	ATCGAA-TCT	266
JQ070079.1	A--TGCGATA	AGTAGTGTGA	ATTGCAGAAT	TCAGTGAATC	ATCGAA-TCT	287
KX179480.1_Alternaria_alternata_strain_BHU-LMMT37	A--TGCGATA	AGTAGTGTGA	ATTGCAGAAT	TCAGTGAATC	ATCGAA-TCT	285
KX179479.1_Alternaria_alternata_strain_BHU-LMMT36	A--TGCGATA	AGTAGTGTGA	ATTGCAGAAT	TCAGTGAATC	ATCGAA-TCT	279
KX139153.1_Alternaria_brassicae_strain_BHU-LMMT20	A--TGCGATA	AGTAGTGTGA	ATTGCAGAAT	TCAGTGAATC	ATCGAA-TCT	301
KX179489.1_Alternaria_alternata_strain_BHU-LMMT46	A--TGCGATA	AGTAGTGTGA	ATTGCAGAAT	TCAGTGAATC	ATCGAA-TCT	298
KX139163.1_Alternaria_eichhorniae_strain_BHU-LMMT30	A--TGCGATA	AGTAGTGTGA	ATTGCAGAAT	TCAGTGAATC	ATCGAA-TCT	274
KX179488.1_Alternaria_alternata_strain_BHU-LMMT45	A--TGCGATA	AGTAGTGTGA	ATTGCAGAAT	TCAGTGAATC	ATCGAA-TCT	272
KX179481.1_Alternaria_solani_strain_BHU-LMMT38	A--TGCGATA	AGTAGTGTGA	ATTGCAGAAT	TCAGTGAATC	ATCGAA-TCT	272
KX179487.1_Alternaria_sp._2_MM-2016	A--TGCGATA	AGTAGTGTGA	ATTGCAGAAT	TCAGTGAATC	ATCGAA-TCT	282
KX139164.1_Alternaria_eichhorniae_strain_BHU-LMMT31	A--TGCGATA	AGTAGTGTGA	ATTGCAGAAT	TCAGTGAATC	ATCGAA-TCT	273
KX179490.1_Alternaria_sp._3_MM-2016	A--TGCGATA	AGTAGTGTGA	ATTGCAGAAT	TCAGTGAATC	ATCGAA-TCT	312
KX179477.1_Alternaria_alternata_strain_BHU-LMMT34	A--TGCGATA	AGTAGTGTGA	ATTGCAGAAT	TCAGTGAATC	ATCGAAATCT	286
KX179486.1_Alternaria_sp._1__MM-2016	A--TGCGATA	AGTAGTGTGA	ATTGCAGAAT	TCAGTGAATC	ATCGAA-TCT	282
KX179491.1_Alternaria_sp._4_MM-2016	A--TGCGATA	AGTAGTGTGA	ATTGCAGAAT	TCAGTGAATC	ATCGAA-TCT	319
KX139154.1_Alternaria_brassicae_strain_BHU-LMMT21	A--TGCGATA	AGTAGTGTGA	ATTGCAGAAT	TCAGTGAATC	ATCGAA-TCT	326
KX139152.1_Alternaria_brassicae_strain_BHU-LMMT19	A--TGCGATA	AGTAGTGTGA	ATTGCAGAAT	TCAGTGAATC	ATCGAA-TCT	245
KX139165.1_Alternaria_macrospora_strain_BHU-LMMT32	A--TGCGATA	AGTAGTGTGA	ATTGCAGAAT	TCAGTGAATC	ATCGAA-TCT	279
KX139166.1_Alternaria_macrospora_strain_BHU-LMMT33	A--TGCGATA	AGTAGTGTGA	ATTGCAGAAT	TCAGTGAATC	ATCGAA-TCT	280
KX139150.1_Alternaria_solani_strain_BHU-LMMT17	AGGTGCGATA	AGTAGTGTGA	ATTGCAGAAT	TCAGTGAATC	ATCGAA-TCT	259
KX139151.1_Alternaria_solani_strain_BHU-LMMT18	A--TGCGATA	AGTAGTGTGA	ATTGCAGAAT	TCAGTGAATC	ATCGAA-TCT	342
KX139162.1_Alternaria_porri_strain_BHU-LMMT29	A--TGCGATA	AGTAGTGTGA	ATTGCAGAAT	TCAGTGAATC	ATCGAA-TCT	334
KX139159.1_Alternaria_brassicicola_strain_BHU-LMMT26	A--TGCGATA	AGTAGTGTGA	ATTGCAGAAT	TCAGTGAATC	ATCGAA-TCT	286
KX139160.1_Alternaria_brassicicola_strain_BHU-LMMT27	A--TGCGATA	AGTAGTGTGA	ATTGCAGAAT	TCAGTGAATC	ATCGAA-TCT	292
KX139157.1_Alternaria_tenuissima_strain_BHU-LMMT24	A--TGCGATA	AGTTCTGTGA	ATTGCAGAAT	TCT-CGAATC	ATCGAA-TCT	270
KX139161.1_Alternaria_porri_strain_BHU-LMMT28	A--TGCGATA	AGTAGTGTGA	ATTGCAGAAT	TCAGTGAATC	ATCGAA-TCT	276
KX139155.1_Alternaria_brassicae_strain_BHU-LMMT22	AG-TCCGA--	-GT--TGTAA	TTTGCAGAGG	GCG------C	TTTGG---CT	259
KX139156.1_Alternaria_brassicae_strain_BHU-LMMT23	AG-TCCGA--	-GT--TGTAA	TTTGCAGAGG	GCG------C	TTTGG---CT	133
Consensus	A--TGCGATA	AGTAGTGTGA	ATTGCAGAAT	TCAGTGAATC	ATCGAA-TCT	

Conservation (100% – 0%)

Create PDF files without this message by purchasing novaPDF printer (http://www.novapdf.com)

```
                                                              420                 440
                                                                |                   |
KX118413.1_Alternaria_alternata_strain_BHU-LMMT02          TTGAA--CG- --CA----CA TT-GCGCCCT TTGGTATTC- CAAAGGG-CA 287
KX118415.1_Alternaria_alternata_strain_BHU-LMMT04          TTGAA--CG- --CA----CA TT-GCGCCCT TTGGTATTC- CAAAGGG-CA 290
KX118418.1_Alternaria_alternata_strain_BHU-LMMT07          TTGAA--CG- --CA----CA TT-GCGCCCT TTGGTATTC- CAAAGGG-CA 288
KX118419.1_Alternaria_alternata_strain_BHU-LMMT08          TTGAA--CG- --CA----CA TT-GCGCCCT TTGGTATTC- CAAAGGG-CA 288
KX118421.1_Alternaria_brassicicola_strain_BHU-LMMT10       TTGAA--CG- --CA----CA TT-GCGCCCT TTGGTATTC- CAAAGGG-CA 287
KX118425.1_Alternaria_brassicae_strain_BHU-LMMT14          TTGAA--CG- --CA----CA TT-GCGCCCT TTGGTATTC- CAAAGGG-CA 288
KX118426.1_Alternaria_brassicae_strain_BHU-LMMT15          TTGAA--CG- --CA----CA TT-GCGCCCT TTGGTATTC- CAAAGGG-CA 287
KX118427.1_Alternaria_solani_strain_BHU-LMMT16             TTGAA--CG- --CA----CA TT-GCGCCCT TTGGTATTC- CAAAGGG-CA 288
KX118424.1_Alternaria_brassicicola_strain_BHU-LMMT13       TTGAA--CG- --CA----CA TT-GCGCCCT TTGGTATTC- CAAAGGG-CA 288
KX118414.1_Alternaria_alternata_strain_BHU-LMMT03          TTGAA--CG- --CA----CA TT-GCGCCCT TTGGTATTC- CAAAGGG-CA 289
KX118423.1_Alternaria_brassicicola_strain_BHU-LMMT12       TTGAA--CG- --CA----CA TT-GCGCCCT TTGGTATTC- CAAAGGG-CA 289
KX118412.1_Alternaria_alternata_strain_BHU-LMMT01          TTGAA--CG- --CA----CA TT-GCGCCCT TTGGTATTC- CAAAGGG-CA 285
KX118417.1_Alternaria_alternata_strain_BHU-LMMT06          TTGAA--CG- --CA----CA TT-GCGCCCT TTGGTATTC- CAAAGGG-CA 287
KX118420.1_Alternaria_alternata_strain_BHU-LMMT09          TTGAA--CG- --CA----CA TT-GCGCCCT TTGGTATTC- CAAAGGG-CA 287
KX118422.1_Alternaria_brassicicola_strain_BHU-LMMT11       TTGAA--CG- --CA----CA TT-GCGCCCT TTGGTATTC- CAAAGGG-CA 295
KX179478.1_Alternaria_alternata_strain_BHU-LMMT35          TTGAA--CG- --CA----CA TT-GCGCCCT TTGGTATTC- CAAAGGG-CA 288
KX118416.1_Alternaria_alternata_strain_BHU-LMMT05          TTGAA--CG- --CA----CA TT-GCGCCCT TTGGTATTC- CAAAGGG-CA 281
KX179483.1_Alternaria_alternata_strain_BHU-LMMT40          TTGAA--CG- --CA----CA TT-GCGCCCT TTGGTATTC- CAAAGGG-CA 297
KX179484.1_Alternaria_alternata_strain_BHU-LMMT41          TTGAA--CG- --CA----CA TT-GCGCCCT TTGGTATTC- CAAAGGG-CA 297
KX179485.1_Alternaria_alternata_strain_BHU-LMMT42          TTGAA--CG- --CA----CA TT-GCGCCCT TTGGTATTC- CAAAGGG-CA 300
KX179482.1_Alternaria_solani_strain_BHU-LMMT39             TTGAA--CG- --CA----CA TT-GCGCCCT TTGGTATTC- CAAAGGG-CA 295
KX139158.1_Alternaria_tenuissima_strain_BHU-LMMT25         TTGAA--CG- --CA----CA TT-GCGCCCT TTGGTATTC- CAAAGGG-CA 304
JQ070079.1                                                 TTGAA--CG- --CA----CA TT-GCGCCCT TTGGTATTC- CAAAGGG-CA 325
KX179480.1_Alternaria_alternata_strain_BHU-LMMT37          TTGAA--CG- --CA----CA TT-GCGCCCT TTGGTATTC- CAAAGGG-CA 323
KX179479.1_Alternaria_alternata_strain_BHU-LMMT36          TTGAA--CG- --CA----CA TT-GCGCCCT TTGGTATTC- CAAAGGG-CA 317
KX139153.1_Alternaria_brassicae_strain_BHU-LMMT20          TTGAA--CG- --CA----CA TT-GCGCCCT TTGGTATTC- CAAAGGG-CA 339
KX179489.1_Alternaria_alternata_strain_BHU-LMMT46          TTGAA--CG- --CA----CA TT-GCGCCCT TTGGTATTC- CAAAGGG-CA 336
KX139163.1_Alternaria_eichhorniae_strain_BHU-LMMT30        TTGAA--CG- --CA----CA TT-GCGCCCT TTGGTATTC- CAAAGGG-CA 312
KX179488.1_Alternaria_alternata_strain_BHU-LMMT45          TTGAA--CG- --CA----CA TT-GCGCCCT TTGGTATTC- CAAAGGG-CA 310
KX179481.1_Alternaria_solani_strain_BHU-LMMT38             TTGAA--CG- --CA----CA TT-GCGCCCT TTGGTATTC- CAAAGGG-CA 310
KX179487.1_Alternaria_sp._2_MM-2016                        TTGAA--CG- --CA----CA TT-GCGCCCT TTGGTATTC- CAAAGGG-CA 320
KX139164.1_Alternaria_eichhorniae_strain_BHU-LMMT31        TTGAA--CG- --CAAA--CA TT-GCGCCCT TTGGTATTC- CAAAGGG-CA 313
KX179490.1_Alternaria_sp._3_MM-2016                        TTGAA--CG- --CA----CA TT-GCGCCCT TTGGTATTC- CAAAGGG-CA 350
KX179477.1_Alternaria_alternata_strain_BHU-LMMT34          TTGAA--CG- --CA----CA TT-GCGCCCT TTGGTATTC- CAAAGGG-CA 324
KX179486.1_Alternaria_sp._1__MM-2016                       TTGAA--CG- --CA----CA TT-GCGCCCT TTGGTATTC- CAAAGGG-CA 320
KX179491.1_Alternaria_sp._4_MM-2016                        TTGAA--CG- --CA----CA TT-GCGCCCT TTGGTATTC- CAAAGGG-CA 357
KX139154.1_Alternaria_brassicae_strain_BHU-LMMT21          TTGAA--CG- --CA----CA TT-GCGCCCT TTGGTATTC- CAAAGGG-CA 364
KX139152.1_Alternaria_brassicae_strain_BHU-LMMT19          TTGAA--CG- --CA----CA TT-GCGCCCT TTGGTATTC- CAAAGGGGCA 284
KX139165.1_Alternaria_macrospora_strain_BHU-LMMT32         TTGAA--CG- --CA----CA TT-GCGCCCT TTGGTATTC- CAAAGGG-CA 317
KX139166.1_Alternaria_macrospora_strain_BHU-LMMT33         TTGAAAACG- --CA----CA TT-GCGCCCT TTGGTATTC- CAAAGGG-CA 320
KX139150.1_Alternaria_solani_strain_BHU-LMMT17             TTGAA--CG- --CA----CA TT-GCGCCCT TTGGTATTC- CAAAGGG-CA 297
KX139151.1_Alternaria_solani_strain_BHU-LMMT18             TTGAA--CG- --CA----CA TT-GCGCCCT TTGGTATTC- CAAAGGG-CA 380
KX139162.1_Alternaria_porri_strain_BHU-LMMT29              TTGAA--CG- --CA----CA TT-GCGCCCT TTGGTATTC- CAAAGGG-CA 372
KX139159.1_Alternaria_brassicicola_strain_BHU-LMMT26       TTGAA--CG- --CA----CA TT-GCGCCCT TTGGTATTC- CAAAGGG-CA 324
KX139160.1_Alternaria_brassicicola_strain_BHU-LMMT27       TTGAA--CG- --CA----CA TT-GCGCCCT TTGGTATTC- CAAAGGG-CA 330
KX139157.1_Alternaria_tenuissima_strain_BHU-LMMT24         TTGAA--CG- --CA----CA TTTGCGCCCT TTGGTATTC- CAAAGGG-CA 309
KX139161.1_Alternaria_porri_strain_BHU-LMMT28              TTGAACGGG- --CA----CA TT-GCGCCCC TTGGTATTC- CATGGGG-CA 316
KX139155.1_Alternaria_brassicae_strain_BHU-LMMT22          TTGGCAGCGG TCCAAGTTCC TTGGAACA-- --GGACGTCA CAGAGGGTGA 305
KX139156.1_Alternaria_brassicae_strain_BHU-LMMT23          TTGGCAGCGG TCCAAGTTCC TTGGAACA-- --GGACGTCA CAGAGGGTGA 179

Consensus                                                  TTGAA--CG- --CA----CA TT-GCGCCCT TTGGTATTC- CAAAGGG-CA
```

100%
Conservation
0%

Create PDF files without this message by purchasing novaPDF printer (http://www.novapdf.com)

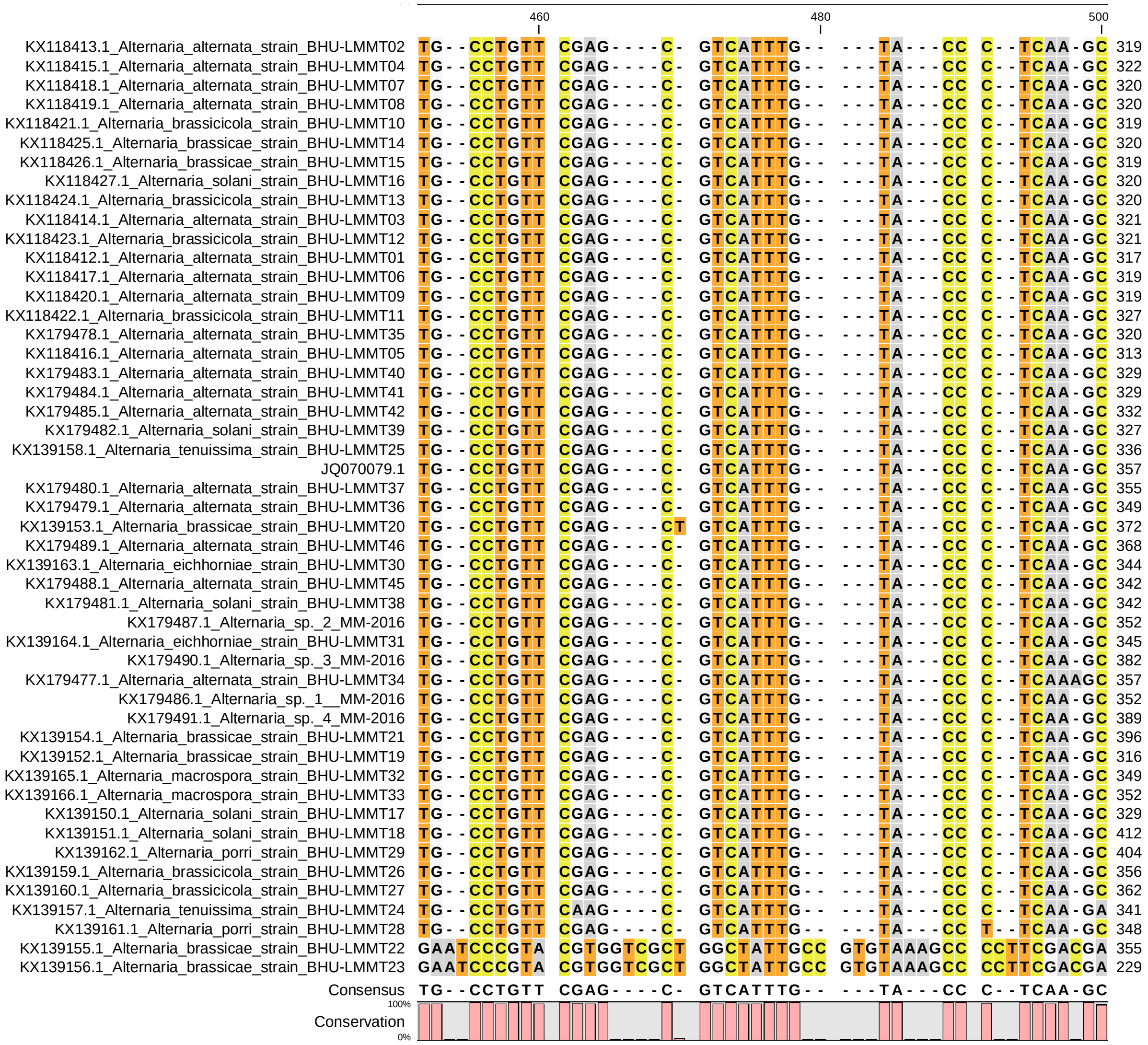

Create PDF files without this message by purchasing novaPDF printer (http://www.novapdf.com)

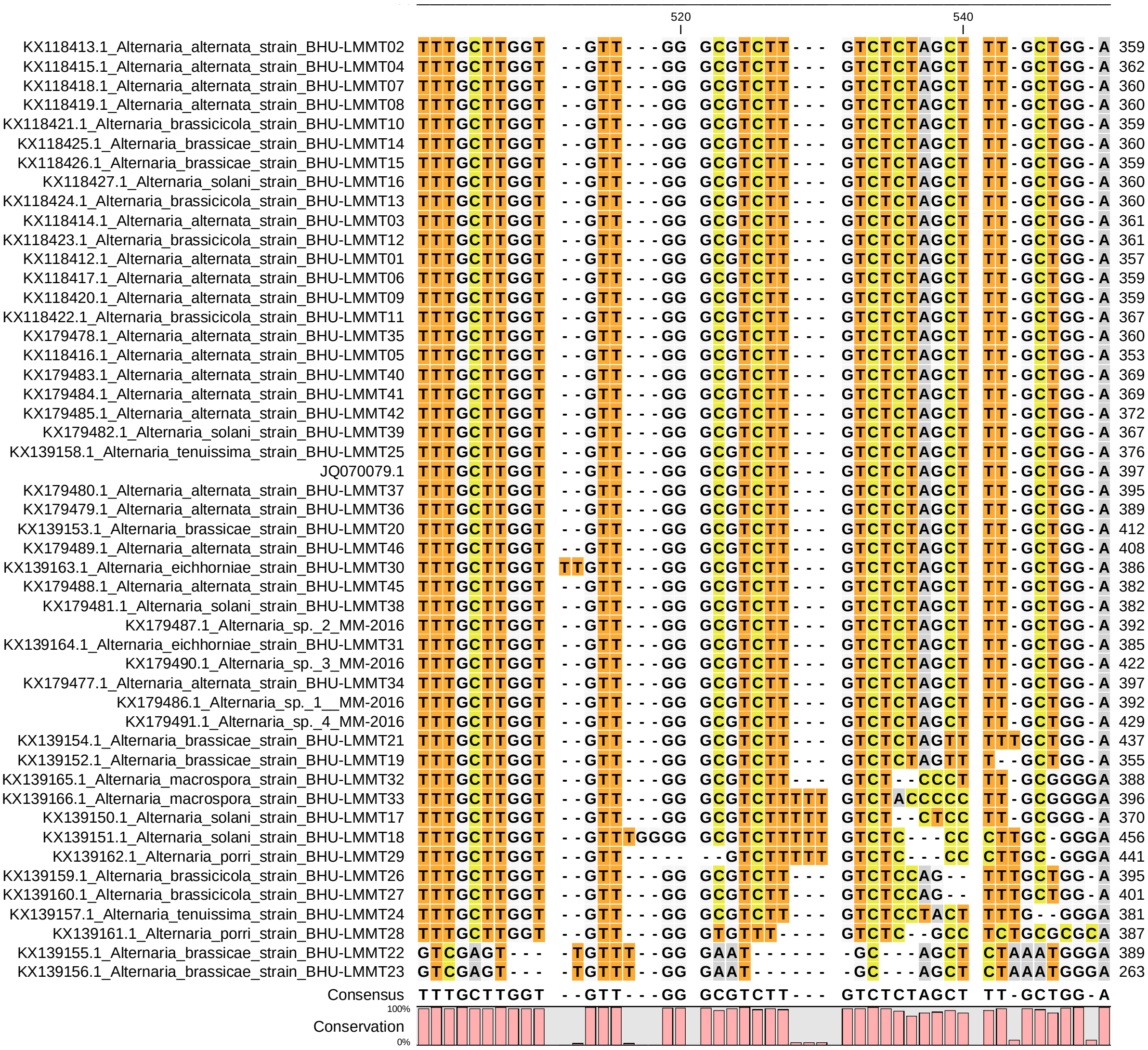

Create PDF files without this message by purchasing novaPDF printer (http://www.novapdf.com)

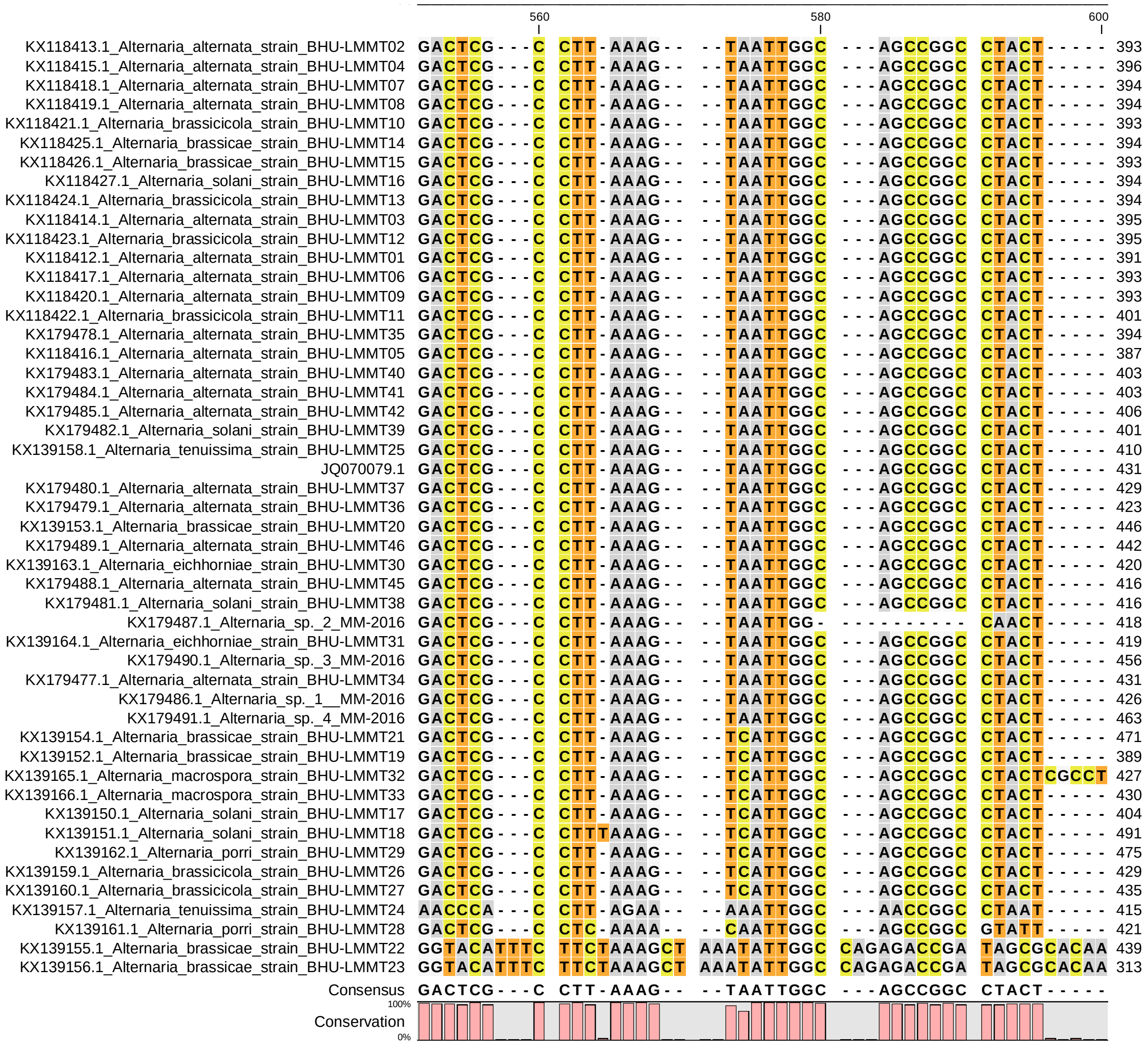

Create PDF files without this message by purchasing novaPDF printer (http://www.novapdf.com)

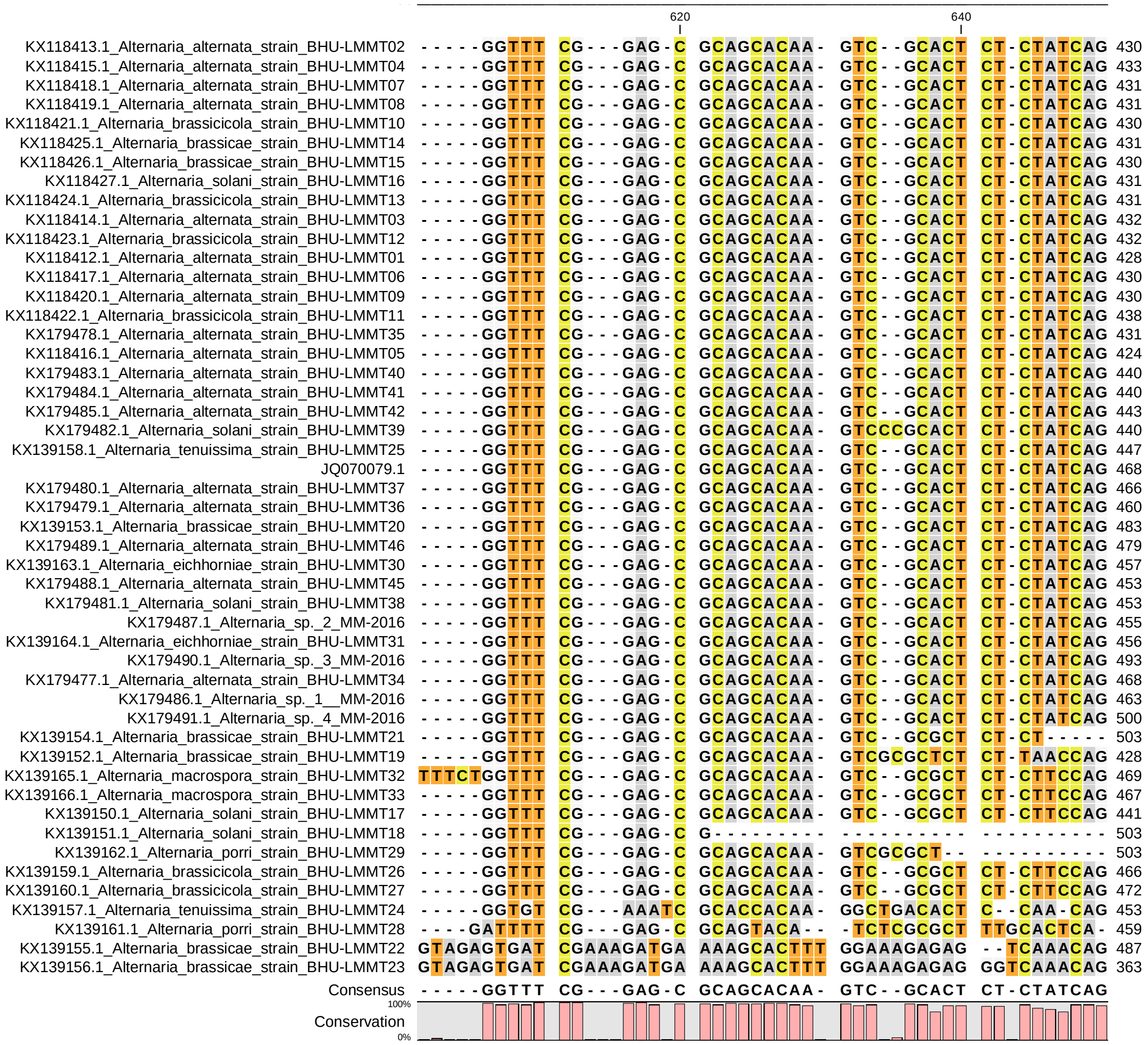

Create PDF files without this message by purchasing novaPDF printer (http://www.novapdf.com)

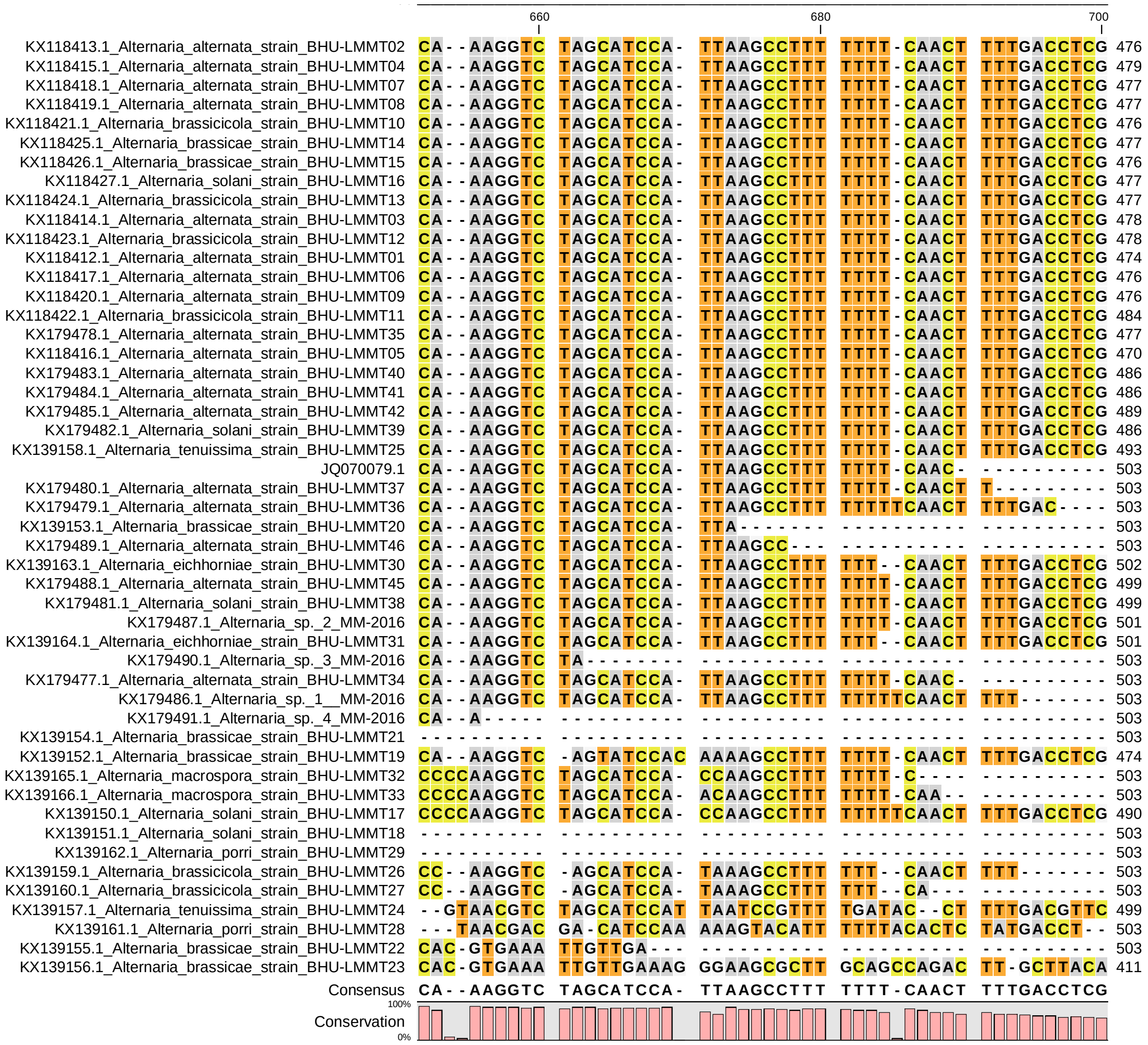

Create PDF files without this message by purchasing novaPDF printer (http://www.novapdf.com)

```
                                                                    720                  740
                                                                     |                    |
KX118413.1_Alternaria_alternata_strain_BHU-LMMT02      G---ATCAGG TAGGGATACC CGCTGAACTT ---------- ---------- 503
KX118415.1_Alternaria_alternata_strain_BHU-LMMT04      G---ATCAGG TAGGGATACC CGCTGAA--- ---------- ---------- 503
KX118418.1_Alternaria_alternata_strain_BHU-LMMT07      G---ATCAGG TAGGGATACC CGCTGAACT- ---------- ---------- 503
KX118419.1_Alternaria_alternata_strain_BHU-LMMT08      G---ATCAGG TAGGGATACC CGCTGAACT- ---------- ---------- 503
KX118421.1_Alternaria_brassicicola_strain_BHU-LMMT10   G---ATCAGG TAGGGATACC CGCTGAACTT ---------- ---------- 503
KX118425.1_Alternaria_brassicae_strain_BHU-LMMT14      G---ATCAGG TAGGGATACC CGCTGAACT- ---------- ---------- 503
KX118426.1_Alternaria_brassicae_strain_BHU-LMMT15      G---ATCAGG TAGGGATACC CGCTGAACTT ---------- ---------- 503
KX118427.1_Alternaria_solani_strain_BHU-LMMT16         G---ATCAGG TAGGGATACC CGCTGAACT- ---------- ---------- 503
KX118424.1_Alternaria_brassicicola_strain_BHU-LMMT13   G---ATCAGG TAGGGATACC CGCTGAACT- ---------- ---------- 503
KX118414.1_Alternaria_alternata_strain_BHU-LMMT03      G---ATCAGG TAGGGATACC CGCTGAAC-- ---------- ---------- 503
KX118423.1_Alternaria_brassicicola_strain_BHU-LMMT12   G---ATCAGG TAGGGATACC CGCTGAAC-- ---------- ---------- 503
KX118412.1_Alternaria_alternata_strain_BHU-LMMT01      G---ATCAGG TAGGGATACC CGCTGAACTT AA-------- ---------- 503
KX118417.1_Alternaria_alternata_strain_BHU-LMMT06      G---ATCAGG TAGGGATACC CGCTGAACTT ---------- ---------- 503
KX118420.1_Alternaria_alternata_strain_BHU-LMMT09      G---ATCAGG TAGGGATACC CGCTGAACTT ---------- ---------- 503
KX118422.1_Alternaria_brassicicola_strain_BHU-LMMT11   G---ATCAGG TAGGGATACC CG-------- ---------- ---------- 503
KX179478.1_Alternaria_alternata_strain_BHU-LMMT35      G---ATCAGG TAGGGATACC CGCTGAACT- ---------- ---------- 503
KX118416.1_Alternaria_alternata_strain_BHU-LMMT05      G---ATCAGG TAGGGATACC CGCTGAACTT AAGCAT---- ---------- 503
KX179483.1_Alternaria_alternata_strain_BHU-LMMT40      G---ATCAGG TAGGGATACC ---------- ---------- ---------- 503
KX179484.1_Alternaria_alternata_strain_BHU-LMMT41      G---ATCAGG TAGGGATACC ---------- ---------- ---------- 503
KX179485.1_Alternaria_alternata_strain_BHU-LMMT42      G---ATCAGG TAGGGAT--- ---------- ---------- ---------- 503
KX179482.1_Alternaria_solani_strain_BHU-LMMT39         G---ATCAGG TAGGGATACC ---------- ---------- ---------- 503
KX139158.1_Alternaria_tenuissima_strain_BHU-LMMT25     GGGGATCAGG ---------- ---------- ---------- ---------- 503
JQ070079.1                                             ---------- ---------- ---------- ---------- ---------- 503
KX179480.1_Alternaria_alternata_strain_BHU-LMMT37      ---------- ---------- ---------- ---------- ---------- 503
KX179479.1_Alternaria_alternata_strain_BHU-LMMT36      ---------- ---------- ---------- ---------- ---------- 503
KX139153.1_Alternaria_brassicae_strain_BHU-LMMT20      ---------- ---------- ---------- ---------- ---------- 503
KX179489.1_Alternaria_alternata_strain_BHU-LMMT46      ---------- ---------- ---------- ---------- ---------- 503
KX139163.1_Alternaria_eichhorniae_strain_BHU-LMMT30    G--------- ---------- ---------- ---------- ---------- 503
KX179488.1_Alternaria_alternata_strain_BHU-LMMT45      G---ATC--- ---------- ---------- ---------- ---------- 503
KX179481.1_Alternaria_solani_strain_BHU-LMMT38         G---ATC--- ---------- ---------- ---------- ---------- 503
KX179487.1_Alternaria_sp._2_MM-2016                    G---A----- ---------- ---------- ---------- ---------- 503
KX139164.1_Alternaria_eichhorniae_strain_BHU-LMMT31    G---A----- ---------- ---------- ---------- ---------- 503
KX179490.1_Alternaria_sp._3_MM-2016                    ---------- ---------- ---------- ---------- ---------- 503
KX179477.1_Alternaria_alternata_strain_BHU-LMMT34      ---------- ---------- ---------- ---------- ---------- 503
KX179486.1_Alternaria_sp._1__MM-2016                   ---------- ---------- ---------- ---------- ---------- 503
KX179491.1_Alternaria_sp._4_MM-2016                    ---------- ---------- ---------- ---------- ---------- 503
KX139154.1_Alternaria_brassicae_strain_BHU-LMMT21      ---------- ---------- ---------- ---------- ---------- 503
KX139152.1_Alternaria_brassicae_strain_BHU-LMMT19      G---ATCAGG TAGGGATACC CGCTGAACTT AA-------- ---------- 503
KX139165.1_Alternaria_macrospora_strain_BHU-LMMT32     ---------- ---------- ---------- ---------- ---------- 503
KX139166.1_Alternaria_macrospora_strain_BHU-LMMT33     ---------- ---------- ---------- ---------- ---------- 503
KX139150.1_Alternaria_solani_strain_BHU-LMMT17         G---ATCAGG TAGGGA---- ---------- ---------- ---------- 503
KX139151.1_Alternaria_solani_strain_BHU-LMMT18         ---------- ---------- ---------- ---------- ---------- 503
KX139162.1_Alternaria_porri_strain_BHU-LMMT29          ---------- ---------- ---------- ---------- ---------- 503
KX139159.1_Alternaria_brassicicola_strain_BHU-LMMT26   ---------- ---------- ---------- ---------- ---------- 503
KX139160.1_Alternaria_brassicicola_strain_BHU-LMMT27   ---------- ---------- ---------- ---------- ---------- 503
KX139157.1_Alternaria_tenuissima_strain_BHU-LMMT24     G---ATC--- ---------- ---------- ---------- ---------- 503
KX139161.1_Alternaria_porri_strain_BHU-LMMT28          ---------- ---------- ---------- ---------- ---------- 503
KX139155.1_Alternaria_brassicae_strain_BHU-LMMT22      ---------- ---------- ---------- ---------- ---------- 503
KX139156.1_Alternaria_brassicae_strain_BHU-LMMT23      GTTGCTCATC CGGGCTTTTG CCCGGTGCAC TCTTCTGTAG GCAGGCCAGC 461
Consensus                                              G---ATCAGG ---------- ---------- ---------- ----------
```

Conservation

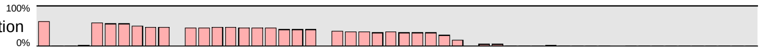

Create PDF files without this message by purchasing novaPDF printer (http://www.novapdf.com)

	760 780	
KX118413.1_Alternaria_alternata_strain_BHU-LMMT02	---------- ---------- ---------- ---------- --	503
KX118415.1_Alternaria_alternata_strain_BHU-LMMT04	---------- ---------- ---------- ---------- --	503
KX118418.1_Alternaria_alternata_strain_BHU-LMMT07	---------- ---------- ---------- ---------- --	503
KX118419.1_Alternaria_alternata_strain_BHU-LMMT08	---------- ---------- ---------- ---------- --	503
KX118421.1_Alternaria_brassicicola_strain_BHU-LMMT10	---------- ---------- ---------- ---------- --	503
KX118425.1_Alternaria_brassicae_strain_BHU-LMMT14	---------- ---------- ---------- ---------- --	503
KX118426.1_Alternaria_brassicae_strain_BHU-LMMT15	---------- ---------- ---------- ---------- --	503
KX118427.1_Alternaria_solani_strain_BHU-LMMT16	---------- ---------- ---------- ---------- --	503
KX118424.1_Alternaria_brassicicola_strain_BHU-LMMT13	---------- ---------- ---------- ---------- --	503
KX118414.1_Alternaria_alternata_strain_BHU-LMMT03	---------- ---------- ---------- ---------- --	503
KX118423.1_Alternaria_brassicicola_strain_BHU-LMMT12	---------- ---------- ---------- ---------- --	503
KX118412.1_Alternaria_alternata_strain_BHU-LMMT01	---------- ---------- ---------- ---------- --	503
KX118417.1_Alternaria_alternata_strain_BHU-LMMT06	---------- ---------- ---------- ---------- --	503
KX118420.1_Alternaria_alternata_strain_BHU-LMMT09	---------- ---------- ---------- ---------- --	503
KX118422.1_Alternaria_brassicicola_strain_BHU-LMMT11	---------- ---------- ---------- ---------- --	503
KX179478.1_Alternaria_alternata_strain_BHU-LMMT35	---------- ---------- ---------- ---------- --	503
KX118416.1_Alternaria_alternata_strain_BHU-LMMT05	---------- ---------- ---------- ---------- --	503
KX179483.1_Alternaria_alternata_strain_BHU-LMMT40	---------- ---------- ---------- ---------- --	503
KX179484.1_Alternaria_alternata_strain_BHU-LMMT41	---------- ---------- ---------- ---------- --	503
KX179485.1_Alternaria_alternata_strain_BHU-LMMT42	---------- ---------- ---------- ---------- --	503
KX179482.1_Alternaria_solani_strain_BHU-LMMT39	---------- ---------- ---------- ---------- --	503
KX139158.1_Alternaria_tenuissima_strain_BHU-LMMT25	---------- ---------- ---------- ---------- --	503
JQ070079.1	---------- ---------- ---------- ---------- --	503
KX179480.1_Alternaria_alternata_strain_BHU-LMMT37	---------- ---------- ---------- ---------- --	503
KX179479.1_Alternaria_alternata_strain_BHU-LMMT36	---------- ---------- ---------- ---------- --	503
KX139153.1_Alternaria_brassicae_strain_BHU-LMMT20	---------- ---------- ---------- ---------- --	503
KX179489.1_Alternaria_alternata_strain_BHU-LMMT46	---------- ---------- ---------- ---------- --	503
KX139163.1_Alternaria_eichhorniae_strain_BHU-LMMT30	---------- ---------- ---------- ---------- --	503
KX179488.1_Alternaria_alternata_strain_BHU-LMMT45	---------- ---------- ---------- ---------- --	503
KX179481.1_Alternaria_solani_strain_BHU-LMMT38	---------- ---------- ---------- ---------- --	503
KX179487.1_Alternaria_sp._2_MM-2016	---------- ---------- ---------- ---------- --	503
KX139164.1_Alternaria_eichhorniae_strain_BHU-LMMT31	---------- ---------- ---------- ---------- --	503
KX179490.1_Alternaria_sp._3_MM-2016	---------- ---------- ---------- ---------- --	503
KX179477.1_Alternaria_alternata_strain_BHU-LMMT34	---------- ---------- ---------- ---------- --	503
KX179486.1_Alternaria_sp._1__MM-2016	---------- ---------- ---------- ---------- --	503
KX179491.1_Alternaria_sp._4_MM-2016	---------- ---------- ---------- ---------- --	503
KX139154.1_Alternaria_brassicae_strain_BHU-LMMT21	---------- ---------- ---------- ---------- --	503
KX139152.1_Alternaria_brassicae_strain_BHU-LMMT19	---------- ---------- ---------- ---------- --	503
KX139165.1_Alternaria_macrospora_strain_BHU-LMMT32	---------- ---------- ---------- ---------- --	503
KX139166.1_Alternaria_macrospora_strain_BHU-LMMT33	---------- ---------- ---------- ---------- --	503
KX139150.1_Alternaria_solani_strain_BHU-LMMT17	---------- ---------- ---------- ---------- --	503
KX139151.1_Alternaria_solani_strain_BHU-LMMT18	---------- ---------- ---------- ---------- --	503
KX139162.1_Alternaria_porri_strain_BHU-LMMT29	---------- ---------- ---------- ---------- --	503
KX139159.1_Alternaria_brassicicola_strain_BHU-LMMT26	---------- ---------- ---------- ---------- --	503
KX139160.1_Alternaria_brassicicola_strain_BHU-LMMT27	---------- ---------- ---------- ---------- --	503
KX139157.1_Alternaria_tenuissima_strain_BHU-LMMT24	---------- ---------- ---------- ---------- --	503
KX139161.1_Alternaria_porri_strain_BHU-LMMT28	---------- ---------- ---------- ---------- --	503
KX139155.1_Alternaria_brassicae_strain_BHU-LMMT22	---------- ---------- ---------- ---------- --	503
KX139156.1_Alternaria_brassicae_strain_BHU-LMMT23	ATCAGTTTGG GCGGTAGGAT AAAGGTCTCT GTCACGTACC TC	503
Consensus	---------- ---------- ---------- ---------- --	
Conservation (100% / 0%)		

Create PDF files without this message by purchasing novaPDF printer (http://www.novapdf.com)
